# Current trends regarding types, properties, self-healing mechanisms, and therapeutic strategies for diabetic wounds addressed with polysaccharide-based self-repairing hydrogels: a review

**DOI:** 10.7150/thno.132479

**Published:** 2026-04-23

**Authors:** Hongkun Xue, Yuxin Bian, Ruoshi Zhang, Ziye Yuan, Haipeng Liu, Chuan Wang, Jiaqi Tan

**Affiliations:** 1 College of Traditional Chinese Medicine, Hebei University, No. 342 Yuhua East Road, Lianchi District, Baoding, 071000, China.; 2 School of Clinical Medicine, Hebei University, No. 342 Yuhua East Road, Lianchi District, Baoding, 071000, China.; 3 Department of Neurosurgery, Affiliated Hospital of Hebei University, No. 212 Yuhua East Road, Lianchi District, Baoding, 071000, China.; 4 Central Laboratory, Affiliated Hospital of Hebei University, No. 212 Yuhua East Road, Lianchi District, Baoding, 071000, China.

**Keywords:** polysaccharide, self-healing, hydrogel, therapeutic strategies, diabetic wound

## Abstract

As the global prevalence of diabetes continues to rise, approximately 19% to 34% of diabetes patients will develop chronic wounds or ulcers, which significantly impacts the quality of life of these patients and also imposes a heavy economic burden on the global healthcare system. The traditional treatment methods (debridement, antibiotics, regular dressing changes, hyperbaric oxygen therapy, etc.) are facing numerous challenges, including the increasing problem of bacterial resistance, high treatment costs, and poor long-term efficacy. Therefore, developing efficient and safe new treatment strategies for diabetic wounds has become a current research hotspot. Polysaccharide-based self-healing hydrogels (PSHs) have become one of the most promising wound dressings due to their excellent self-healing ability, good biodegradability, biocompatibility, and multiple biological activities. Unfortunately, there are still limitations in the research on PSHs in the field of diabetes wound treatment. Firstly, this review comprehensively introduces the types of polysaccharides with therapeutic effects on diabetic wounds and their excellent properties. Subsequently, this article systematically reviews the self-healing mechanisms of PSHs and the therapeutic strategies for diabetic wounds. Finally, this paper reviews the challenges and future prospects of PSHs in the clinical transformation of diabetic wounds. The results provide an important reference for the application of PSHs in the field of diabetes wound treatment.

## 1. Introduction

Diabetes mellitus is a metabolic disease characterized by hyperglycemia resulting from insufficient insulin secretion/insulin resistance, which is highly prevalent worldwide 1. According to the latest report from the International Diabetes Federation (IDF), it is estimated that the number of people with diabetes worldwide will increase to 643 million by 2030, and approximately 783 million by 2045 2. Diabetes can cause a series of serious complications. Approximately 19% to 34% of diabetes patients will develop wounds or ulcers, which impose a heavy economic burden on the global healthcare system 3. Compared with ordinary wounds, diabetic wounds have more complex pathological micro-environment. Diabetic wounds are often accompanied by high blood sugar and oxidative stress, which can easily cause serious problems, including continuous inflammatory stimulation, bacterial infection, and angiogenesis disorders around the wound surface, thus leading to diabetic wounds healing disorders 4. Long-term exposure of the wounds can lead to gradual deterioration and even amputation, which has a serious impact on the quality of life of patients 5.

Currently, the clinical methods for treating diabetic wounds mainly include debridement, application of antibiotics, wound dressings (hydrocolloid dressings, foam dressings, and silver ion dressings), and negative pressure therapy 6. Nevertheless, the aforementioned methods have numerous limitations in the management of diabetic wounds. In addition to causing pain and discomfort, surgical debridement also exposes deeper tissues, thereby causing secondary damage to the wounds. Long-term utilization of antibiotics can lead to an increase in the proportion of drug-resistant bacteria. For instance, the emergence of methicillin-resistant *Staphylococcus aureus* (MRSA) and *Pseudomonas aeruginosa* has made wound infection control increasingly difficult 7,8. Negative pressure therapy can effectively remove exudate, reduce edema, and promote the growth of granulation tissue. However, the negative pressure therapy equipment is expensive and requires patients to remain in bed for a long time, which significantly increases the economic burden and treatment discomfort 9. Wound dressings are an indispensable treatment for diabetic wounds. Currently, the mainstream wound dressings in clinical practice mainly include hydrocolloid dressings, foam dressings, and silver ion dressings. Notably, these dressings still face some challenges in actual treatment. The ability of hydrocolloid dressings to absorb exudate is limited, which can easily lead to frequent impregnation and replacement, thus damaging the newly formed granulation tissue. Notably, foam dressings have high absorbency. Unfortunately, foam dressings lack antibacterial activity. Moreover, the opacity of foam dressings hinders real-time observation of the wound 10. Silver ion dressings have higher antibacterial activity than foam dressings. However, the cytotoxic risk of silver ion dressings may delay tissue regeneration. Besides, the long-term application of silver ions may lead to accumulation in the liver and kidneys, thus causing systemic safety concerns 11. Hence, we urgently need to develop both biocompatibility, environmental adaptability, and multi-functionality of new wound dressings, thereby promoting diabetic wound healing and skin regeneration. In this context, polysaccharide-based self-healing hydrogels (PSHs) have attracted much attention in the field of diabetic wound treatment due to their excellent biocompatibility, degradability, and multiple biological activities (antibacterial, anti-inflammatory, antioxidant, etc.). Compared with the aforementioned treatment methods (debridement, negative pressure therapy, and traditional dressings), PSHs demonstrate significant clinical application advantages as follows 12: 1) Cost controllable: Natural polysaccharides have a wide source and relatively simple preparation process, which can reduce medical costs and overcome the limitation of heavy economic burden of negative pressure treatment; 2) Function integration: PSHs have multiple biological activities (antibacterial, anti-inflammatory, antioxidant, etc.), which make up for the single function of traditional dressings; 3) Self-repairing properties: PSHs can autonomously restore the network structure after being damaged, thus extending the service life and reducing the frequency of replacement; 4) Biocompatibility: PSHs possess excellent biocompatibility, which can reduce cytotoxicity and accumulation risks.

Polysaccharides, as natural macromolecular compounds, are ideal scaffold materials for constructing PSHs. Natural polysaccharides mainly include chitosan (CS), hyaluronic acid (HA), alginate, cellulose, etc. Polysaccharides possess excellent biocompatibility, degradability, and multiple biological activities (antibacterial, antioxidant, immunomodulatory, etc.). Additionally, polysaccharides have higher environmental stability than other natural polymers (such as proteins) 13,14. More importantly, the abundant active groups (hydroxyl, carboxyl, and amino groups) on the polysaccharide molecular chains are highly susceptible to chemical modification or composite modification, which can endow hydrogels with stimulus-responsive properties, drug loading capacity, and self-healing performance 15. The self-healing properties, as the key innovation point of PSHs, can be formed through dynamic and reversible chemical bonds (Schiff base bonds, borate ester bonds, disulfide bonds, etc.) or physical interactions (hydrogen bonds, metal coordination, hydrophobic interactions, ionic interactions, etc.). In addition, the self-healing properties can enable hydrogels to autonomously restore their functional and structural integrity after being damaged or broken, which contributes to them adapting to the dynamic changes of the wound surface, extending the service life of the dressing, and reducing the frequency of replacement and the risk of secondary injury 16,17. At present, PSHs have made significant progress in the field of diabetic wound treatment. For instance, Xuan *et al*. 18 prepared a novel injectable, self-healing, and multi-active polysaccharide hydrogel (PGHAA). The hydrogel exhibited excellent antibacterial activity, hemostatic properties, and immunomodulatory capabilities. Importantly, the hydrogel could achieve rapid wound healing in diabetes (within 11 d), which provided a feasible treatment strategy for diabetic wound management. Similarly, Qiao *et al*. 19 designed pH/ROS-responsive intelligent hydrogels. The hydrogels could respond to microenvironmental stimuli and intelligently regulate drug release, thereby improving the pathological microenvironment of diabetic wounds and accelerating the healing process (with a 96.2% healing rate after 14 d). Consequently, PSHs are gradually moving towards the direction of intelligent and multi-functional collaborative treatment.

Currently, there have been several reviews that have systematically summarized the research on hydrogels in the field of diabetic wounds. For example, Yan *et al*. 1 comprehensively summarized the various design strategies, therapeutic effects, and mechanisms of bio-enhanced hydrogels loaded with extracellular vesicles. This review was mainly focused on specific bioactive carriers (extracellular vesicles). We started from various therapeutic strategies (antibacterial, antioxidant, anti-inflammatory, immunomodulatory, etc.) and systematically expounded the application of various advantageous functional components (nanomaterials, polyphenolic compounds, growth factors, and photosynthetic biomaterials) in the repair of diabetic wounds. Chen *et al*. 2 summarized the latest advancements of intelligent responsive hydrogel dressings in the treatment of diabetic wounds and focused on the design of hydrogel structure, response principles, and degradation behavior. This review lacked the selection of raw materials for preparation and the summary of specific treatment strategies. Overall, most previous studies have focused on specific materials/single-function responsive hydrogels. Currently, there is no comprehensive review that systematically integrates and organizes the polysaccharide backbone materials, self-repair mechanisms, and diabetes wound treatment strategies of PSHs. To fill this gap, this paper reviews the polysaccharide backbone materials and functional properties of PSHs. Additionally, this paper systematically elaborates on the self-repair mechanisms of PSHs, including physical non-covalent bonds (hydrogen bonds, hydrophobic interactions, host-guest interactions, electrostatic interactions, and metal coordination) and chemical covalent bonds (imine bonds, amidoxime bonds, disulfide bonds, borate ester bonds, and DA reactions). Furthermore, this paper highlights the therapeutic strategies of PSHs (antibacterial, antioxidant, anti-inflammatory, and immunomodulatory, angiogenic promotion, hypoglycemic, and hypoxia alleviation), which effectively demonstrate the significant application potential of PSHs in the field of diabetes wound treatment. Finally, this paper summarizes and discusses the challenges and future prospects of PSHs in terms of large-scale production and production costs, sterilization methods and long-term storage, biological safety, clinical operational convenience, and regulatory approval. All in all, this paper aims to systematically review the entire chain of PSHs from raw materials to treatment strategies, which provide theoretical references and technical guidance for in-depth research and efficient application of PSHs in the field of diabetic wound treatment, thus promoting wound healing and improving patient prognosis.

## 2. Review method

This paper strictly follows the basic framework of a systematic literature review and conducted a comprehensive search and screening of the application of PSHs in the field of diabetes wound treatment. The search covers major academic databases, such as PubMed, Web of Science, Science Direct, and Google Scholar. The literature search was conducted with the time window set from 2018 to 2026, and the latest studies published between 2022 and 2026 are given priority. The search strategy adopted a combination of keywords for querying. The complete query string was retrieved through Boolean operators (AND, OR, NOT) as follows: “polysaccharide-based self-healing hydrogels”, “chitosan self-healing hydrogels”, “alginate self-healing hydrogels”, “hyaluronic acid self-healing hydrogels”, “cellulose self-healing hydrogels”, “diabetic wound healing”, “chronic wound management”, “self-healing mechanism”, and “stimuli-responsive hydrogels”. Inclusion criteria are as follows: 1) The research topic focuses on the application of PSHs in the treatment of diabetic wounds/chronic wounds; 2) The research types include original studies, reviews, and preclinical/clinical studies; 3) The language of publication is English; 4) The publication is in a peer-reviewed journal. Exclusion criteria include the following: 1) Conference abstracts, patents, editorials, and non-peer-reviewed literature; 2) Studies not directly related to diabetic wounds; 3) Studies with low relevance to the topic of PSHs; 4) Literature with incomplete data or unable to obtain full text. The literature screening process is mainly divided into three stages as follows: 1) The initial screening excludes irrelevant literature based on the title and abstract; 2) The re-screening assesses the studies that meet the inclusion criteria through full-text reading; 3) The final screening supplements potential omissions by tracing the references. This article aims to comprehensively and objectively review the research progress of PSHs in the field of diabetic wound treatment through the above search and screening strategies, which can provide a reliable literature basis for subsequent studies.

## 3. Wound healing mechanisms

### 3.1. Normal wounds

Wound healing consists of four consecutive stages, including hemostasis, inflammation, proliferation, and remodeling 20. Wounds possess unique cellular behaviors and molecular mechanisms at each stage of healing. These behaviors and mechanisms work in synergy, thus jointly promoting tissue repair and functional recovery 21,22. Hemostasis, as the initial stage of wound healing, occurs immediately after injury and rapidly seals the broken vessels, thereby preventing further blood loss 23. Vascular damage could disrupt the integrity of vascular endothelium, thus triggering platelet adhesion and activation 24. Firstly, blood vessels constrict, and then platelets bind to the damaged subcutaneous collagen through the GPIa-IIa receptor. Meanwhile, platelets bind to von Willebrand factor (vWF) by the GPIb-IX-V receptor, and then rapidly adhere to the damaged endothelium of the bleeding wound 25. Subsequently, platelets release mediators, including ADP, TXA2, and 5-HT, further recruiting and activating peripheral platelets to form platelet thrombi 25,26. Finally, endogenous and exogenous coagulation cascades are activated 27. The difference between the two coagulation pathways lies in their initiation mechanisms and the involved coagulation factors. Both coagulation pathways promote the formation of thrombin by activating factor X, which can lead to the conversion of fibrinogen into a fibrin network, forming a stable blood clot, and achieving hemostasis 28.

The inflammatory phase begins within a few hours of wound injury. This phase is jointly promoted by bacterial metabolic products, cytokines and chemokines secreted by immune cells, and platelet-derived mediators 29. Mast cells release enzymes, histamine, and 5-HT to promote vascular dilation after wound injury, thereby inducing neutrophils and monocytes to exudate into the wound surface through blood vessels 24,30. Neutrophils first reach the wound site and clear bacteria and cell debris via their phagocytosis 27,31. Besides, neutrophils release numerous ROS, proteases, and cationic peptides to degrade necrotic tissues and resist microbial infections 30. Monocytes migrate to the injured site approximately 3 d after injury and then differentiate into macrophages 26. Macrophages can engulf pathogens, clear tissue debris, and secrete a series of growth factors (IGF-1, VEGF, and PDGF) 25,32,33. These cells and growth factors promote the proliferation of fibroblasts and capillaries through synergistic action, thus facilitating wound healing and entering the proliferation stage 34.

The proliferative phase is the core stage of wound healing. This stage mainly includes angiogenesis, granulation tissue formation, and re-epithelialization 35. Angiogenesis is regulated by various factors. EGF and VEGF can stimulate endothelial cells to migrate to wounds at the molecular level, thereby promoting the formation of new vascular intima. New capillaries can not only provide more oxygen and nutrition for wounds, but also effectively remove metabolic waste and inflammatory mediators, which can provide a favorable environment for subsequent repair 36. Histologic features of granulation tissue formation include fibroblasts, new blood vessels, cutin cells, endothelial cells, etc. 37. Firstly, fibroblasts synthesize large amounts of type III collagen during the ECM remodeling process. Subsequently, fibroblasts gradually differentiated into myofibroblasts with contractile function under the regulation of TGF-β. This phenotypic transformation is crucial for wound contraction 22,37,38. Another important step in the proliferative phase is re-epithelialization. This step begins with the proliferation of keratinocytes at the wound edge 39. Keratinocytes can migrate along the surface of granulation tissue to the wound center by directional migration. Meanwhile, basal layer cells establish connections with keratinocytes from the wound edge through proliferation, thereby completing wound coverage 26,40.

The remodeling stage, as the final stage of wound healing, involves the structural reorganization and functional maturation of the newly formed matrix. The remodeling stage begins approximately 2-3 weeks after wound formation and sometimes lasts for years 41. The remodeling stage can occur a series of changes. Collagen fibers undergo remodeling (type III collagen is replaced by type I collagen) to enhance their mechanical properties, which is conducive to strengthening the tensile strength of wound repair or regenerated skin 42. In addition, matrix metalloproteinases (MMPs) degrade the excess ECM components 43. Notably, the remodeling process is accompanied by programmed cell death in most cells. Failure of programmed cell death in granulation tissue can cause hypertrophic scar formation 44.

### 3.2. Diabetic wound

**Figure 1:**
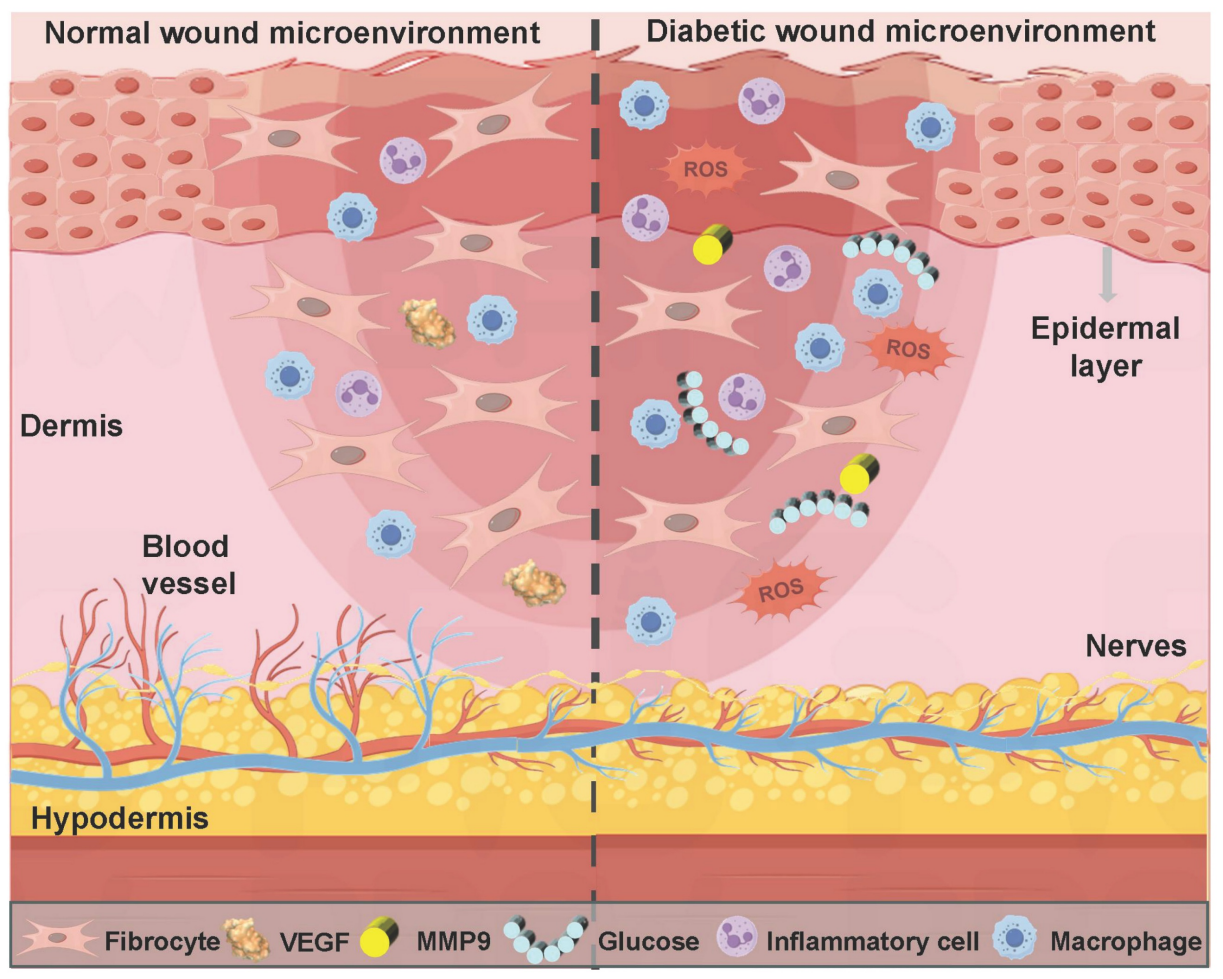
Comparison of the microenvironment of normal wounds and diabetic wounds.

The healing process of diabetic wounds deviates from the conventional steps. This process can be halted at any stage of normal wound healing and eventually develop into a chronic wound 45. Impaired platelet function hinders the coagulation pathway in the hemostasis stage, thus leading to wound bleeding. High blood sugar levels can cause the accumulation of ROS and the long-term secretion of inflammatory factors (TNF-α and IL-6) in the inflammatory stage, thereby leading to persistent inflammatory response symptoms at the wound site. Diabetic wound microenvironment leads to impaired endothelial cell and fibroblast function in the stage of proliferation and remodeling, thus causing tissue aplasia 46. In summary, compared with normal wound healing, diabetic wounds at different stages exhibit a more complex pathological microenvironment (Figure 1). The properties of diabetic wounds include high glucose levels, hypoxia, infection, abnormal pH values, ROS accumulation, persistent inflammation, and abnormal MMPs levels 47,48. Table 1 shows the comparison of normal wounds and diabetic wounds at different healing stages.

Firstly, the local high glucose microenvironment of the wounds can inhibit the TGF-β/Smad3 pathway in fibroblasts and reduce collagen deposition, which is crucial for skin epithelialization during wound healing 49,50. Secondly, hyperglycemia can activate the PKC-NOX pathway and the hexosamine metabolic pathway, deplete the NADPH of the polyol pathway, enhance non-enzymatic glycosylation, and disrupt mitochondrial electron transport, which leads to abnormal accumulation of ROS, thus forming a sustained oxidative stress response at the wound site 51. Additionally, hyperglycemia can lead to polarization imbalance of macrophages, promote the secretion of proinflammatory cytokines (IL-1β, IL-6, and TNF-α), and hinder the transformation of macrophages from M1 to M2, thereby inducing chronic inflammatory response 50. Furthermore, advanced glycation end products (AGEs) can be generated through REDOX reactions between glucose and amino groups. AGEs are regarded as the key factor in the pathological deterioration of diabetic wounds 52. Relevant study has confirmed that the mechanisms of AGEs impeding wound healing were as follows 53: 1) AGEs could aggravate chronic inflammation and oxidative stress, form a vicious cycle of inflammation and oxidative stress, and destroy the balance of wound microenvironment; 2) AGEs could directly interfere with the normal functions of keratinocytes (cell viability, migration, and differentiation ability), thereby inhibiting the formation of the epidermal barrier; 3) AGES-induced inflammatory keratinocytes could further affect other surrounding skin cells, and eventually form refractory wounds under high blood glucose environment.

O_2_, as a metabolic substrate and signaling molecule for various physiological processes, plays a crucial role in maintaining normal physiological activities. In particular, O_2_ plays an irreplaceable role in the wound repair process, including preventing wound infection, stimulating re-epithelialization, synthesizing collagen, and promoting angiogenesis 54. Nevertheless, microvascular lesions induced by diabetes can lead to lumen stenosis and microcirculation disorders, thereby hindering the delivery of O_2_ to the wound site 55. Moreover, the proliferation of anaerobic bacteria at the wound site can trigger an inflammatory response, thus activating the NADPH oxidase system and ultimately creating a hypoxic environment by consuming large amounts of O_2_. The hypoxic environment will intensify the proliferation of anaerobic bacteria, sustain inflammatory responses, and form a vicious cycle 56. Compared with acute wounds, diabetic wounds are in a chronic hypoxic state for a longer period of time (imbalance between high oxygen consumption and low oxygen supply), which poses a continuous challenge to wound healing 57. Hypoxia-inducible factor-1α (HIF-1α) can be stably expressed in a hypoxic environment. HIF-1α regulates the adaptive response of cells to hypoxia and restores cell metabolism by regulating the expression of genes related to cell survival, metabolism, and angiogenesis. In addition, HIF-1α can also promote endothelial cell proliferation and VEGF expression, thus stimulating angiogenesis 58. However, chronic hypoxia can inhibit HIF-1α and VEGF expression, restrain endothelial cell function, and aggravate vascular formation disorders and tissue ischemia, which seriously hinders the healing process of diabetic wounds 59,60. In addition, hypoxia can promote the production of ROS and induce the intensification of tissue damage mediated by oxidative stress, further deteriorating the wound microenvironment 56.

The risk of wound infection significantly increased due to the destruction of skin barrier integrity in diabetic wounds, which makes the wound site susceptible to colonization and invasion by opportunistic pathogens (*Staphylococcus aureus* and *Pseudomonas aeruginosa*) 61,62. Additionally, high glucose and a hypoxic microenvironment at the wound site provide ideal growth conditions for bacteria. After wound infection, tissue damage can be exacerbated through multiple mechanisms, including bacterial infection leading to endothelial cell dysfunction and abnormal fibroblast phenotypes, inducing inflammatory cell aggregation, and causing excessive accumulation of ROS. These pathological changes further lead to an imbalance in ECM degradation, over-expression of pro-inflammatory factors, and obstruction of angiogenesis 57,63. In addition, bacteria can attach to living or non-living contact surfaces through their secreted extracellular viscous substances (polysaccharide matrix, fibrin, lipoprotein, etc.) when stimulated by external environments, thereby forming biofilms. Biofilm formation is more clinically harmful than free-form microbial infection. More than 90% of microorganisms (*Staphylococcus aureus*, *Candida*, and *Pseudomonas aeruginosa*) tend to multiply by forming biofilms 64. Biofilms can increase the resistance of bacteria to conventional antibacterial agents by 10 to 1,000 times 61. Additionally, continuous stimulation of biofilms in chronic wounds can trigger excessive inflammatory responses, thereby hindering the healing of skin wounds 65.

pH on the surface of normal skin is weakly acidic, which can act as a natural barrier to protection. pH gradually becomes neutral with the increase of skin depth 66. Nevertheless, various factors (impaired blood glucose regulation, tissue hypoxia, accumulation of metabolic products, and bacterial infection) can lead to the pH of diabetic wounds usually remaining within the weakly alkaline range of 7.0 to 9.0 67. Alkaline pH has numerous effects on the wound microenvironment as follows 68: 1) Increase affinity of hemoglobin for O_2_ through the Bohr effect, thereby inhibiting O_2_ release; 2) Promote bacterial proliferation and cause repeated wound infections; 3) Induce the transformation of macrophages from M2 type to M1 type, which leads to a persistent inflammatory state; 4) Increase proteolytic enzyme activity, thus leading to degradation of ECM and growth factors, and hindering the process of wound healing. Interestingly, alkaline environments do not always inhibit wound healing. For instance, an important stage of wound healing involves the proliferation and migration of fibroblasts, which typically occurs in alkaline microenvironments 69. Consequently, appropriately adjusting pH at different stages of diabetic wound healing is beneficial for wound repair 70.

ROS plays a crucial role in regulating multiple stages of the wound healing process. Physiological ROS can resist the invasion of pathogenic microorganisms and mediate cell survival signal transduction 71. Nevertheless, hyperglycemia activates mitochondrial electron transport chains and oxidase enzymes in diabetic patients, thus leading to abnormal accumulation of ROS. Moreover, hyperglycemia impairs the antioxidant defense system, thereby decreasing the ROS scavenging capacity. Furthermore, immune cells (neutrophils) release a large amount of ROS after activation, eventually leading to excessive ROS accumulation 72,73. Insufficient clearance and continuous accumulation of ROS can contribute to oxidative stress and significantly hindering the wound healing process. ROS causes damage to biological macromolecules (proteins, DNA, lipids, and carbohydrates) at the molecular level and disrupts the integrity of cell membranes 74. Besides, ROS induces macrophage polarization to M1 type by activating the NLRP3 inflammasome pathway and aggravating the inflammatory response 75. Oxidative stress microenvironment significantly inhibits the migration and proliferation of endothelial cells, fibroblasts, and keratinocytes at the cellular level 76. Additionally, ROS up-regulates the expression and activity of MMPs, thus leading to abnormal degradation of ECM. In summary, the above-mentioned mechanisms work in synergy and hinder the healing of diabetic wounds 75.

Diabetic wounds are infiltrated by numerous inflammatory cells (neutrophils and macrophages). Macrophages, as the main inflammatory cells, play a vital role in driving the inflammatory response of wounds 77. M1-type macrophages are marked by specific proteins, such as CD86, iNOS, and TNF-α. M1-type macrophages can phagocytize microorganisms, stroma, and platelet cell fragments. In addition, M1-type macrophages can secrete pro-inflammatory mediators and chemokines, and recruit more circulating monocytes to participate in phagocytosis, angiogenesis, and re-epithelialization processes 78. M2-type macrophages are dominant in the regression stage and mainly secrete anti-inflammatory and growth factors. Additionally, compared with M1-type macrophages, M2-type macrophages possess higher phagocytic activity, which can effectively remove necrotic and damaged cells on the surface of trauma. Therefore, M2-type macrophages are crucial for wound repair 79. Nevertheless, high levels of oxidative stress and abnormally active energy metabolic bypass in diabetic patients can contribute to absolute dominance of M1 macrophages (proinflammatory) and minimal or complete absence of M2 macrophages (anti-inflammatory) 80,81. This imbalance can be further exacerbated through positive feedback. Substantial M1-type macrophages promote the production of IFN-γ by secreting IL-12 and IL-18, thereby facilitating the polarization of newly recruited monocytes at the inflammatory site towards the M1 phenotype. Extreme imbalance of M1/M2 phenotype can cause diabetic wounds to remain in the inflammatory phase for a long time, thereby failing to smoothly transition to the proliferative phase, which becomes a crucial factor hindering wound healing 82.

MMPs, as one of the main proteolytic enzymes expressed by tissue-forming cells and inflammatory cells, can participate in the entire process of wound healing by regulating ECM 83. MMPs are essential for wound repair. However, abnormal MMPs content has a negative impact on the wound healing process. For instance, the elevated MMPs activity can impair ECM integrity, impede cell proliferation and migration, and delay re-epithelialization and wound closure 84. Unfortunately, persistent inflammation and oxidative stress significantly elevate the levels of MMPs in diabetic wounds, particularly MMP-9 85. A relevant study has indicated that the average concentration of MMP-9 in skin biopsy tissues from diabetic ulcers was 14 times higher than that observed in non-diabetic patients 86. Notably, elevated MMP-9 levels could affect the viability, proliferation, and migration of fibroblasts, inhibit collagen secretion, and ultimately lead to apoptosis 87. Moreover, MMPs homeostasis was specifically regulated by tissue inhibitors of metalloproteinases (TIMP). Nevertheless, research has demonstrated that the down-regulation of TIMP expression in diabetic wounds could result in a significant disruption of the balance between MMPs and TIMP, which significantly hindered the physiological process of wound healing 88.

## 4. The type of PSHs

**Figure 2:**
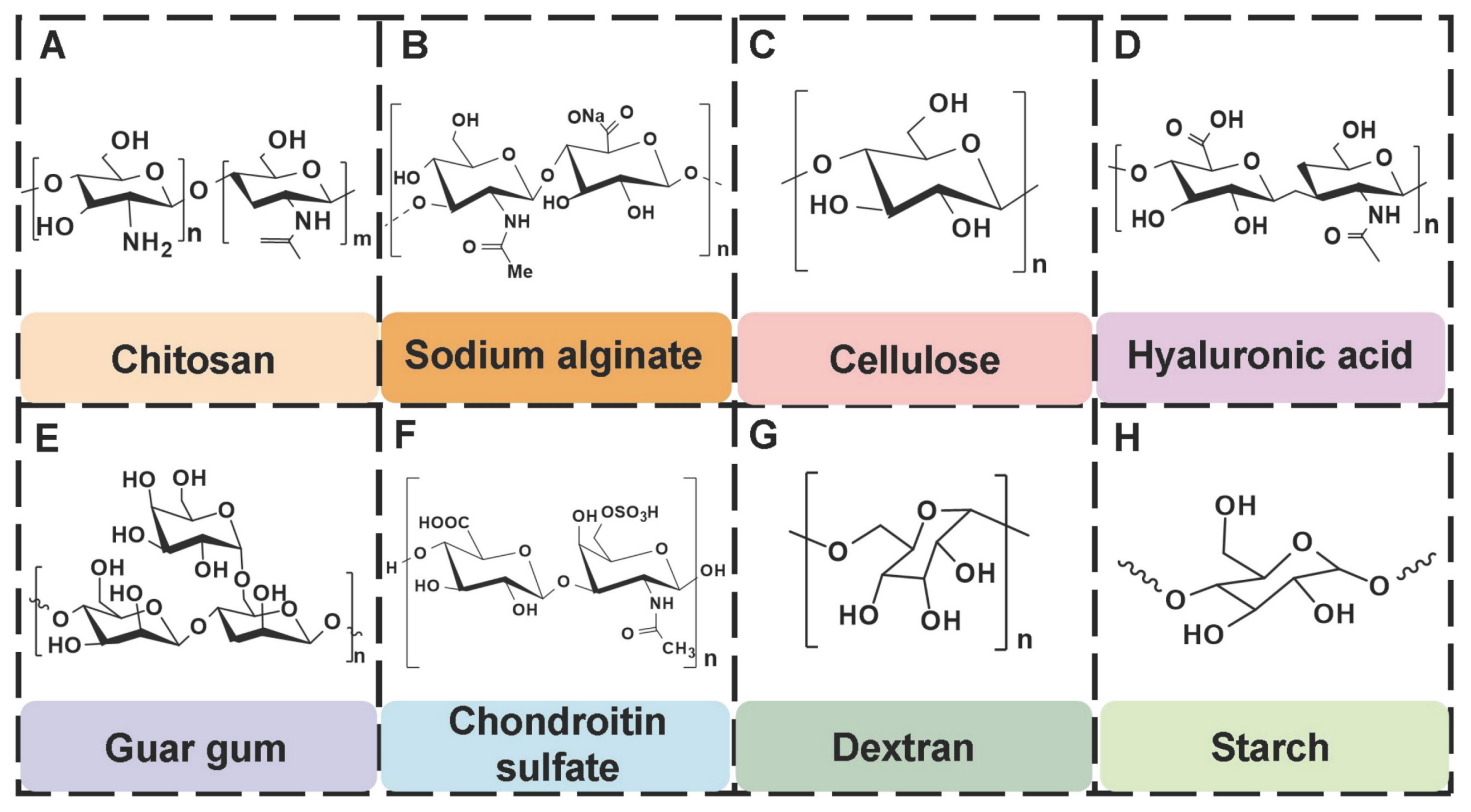
The chemical structures of various polysaccharides.

PSHs possess significant application value in diabetic wound healing. Common polysaccharide matrices include chitosan (CS), sodium alginate (SA), cellulose, hyaluronic acid (HA), etc. Additionally, chondroitin sulfate, starch (ST), and dextran (Dex) have also been widely utilized. Figure 2 represents the chemical structures of various polysaccharides. These polysaccharide materials have attracted much attention due to their excellent biocompatibility and modifiable properties. Polysaccharides can form a three-dimensional network structure through dynamic bonding, thereby endowing hydrogels with self-healing properties. Different types of PSHs possess their characteristics in terms of mechanical properties, biological activities, and functional regulation. In addition, PSHs, as carriers, can also integrate various excellent properties, which lay a solid foundation for their application in the field of diabetic wounds. Different polysaccharides are suitable for specific healing stages and wound types due to their unique structural characteristics in the treatment of diabetic wounds. For example, ST can rapidly concentrate blood, enrich coagulation factors, and accelerate clot formation, thus being suitable for wound hemostasis. CS can effectively control diabetic wound infections and bleeding due to its excellent antibacterial and hemostatic properties, which are beneficial for CS to exert effects during the inflammatory stage of the wound. SA exhibits excellent high hygroscopicity and mild gel-forming properties, which are suitable for managing exudate from moderately to severely exudative diabetic wounds. HA can regulate inflammatory responses, promote angiogenesis, and support cell migration and proliferation, which is attributed to HA's good biocompatibility and non-immunogenicity. Therefore, HA plays a crucial role in the proliferation and remodeling stages of diabetic wounds. Cellulose has good mechanical properties and wound-supporting capabilities, which provide mechanical stability during the wound remodeling period of diabetes 89,90. Hence, different polysaccharide types can be selected based on the type of wound for treatment. This section focuses on discussing the different types and excellent properties of PSHs, thereby demonstrating their applicability in the field of diabetic wounds.

### 4.1. Chitosan

CS, as a multifunctional natural polymer, possesses hydrophilicity, cationicity, and reactivity, which are conducive to the application of CS in various processes, such as functional modification, biomolecular encapsulation, etc. 91. Compared with other natural polymers (SA, HA, cellulose, gelatin, and collagen), CS has more prominent intrinsic biological activity. For instance, SA, HA, cellulose, gelatin, etc. usually lack inherent antibacterial activity, and their hemostatic/regulatory inflammatory functions are relatively limited. Consequently, these polymers require additional chemical modification or the loading of active drugs. CS possesses several inherent advantages, including broad-spectrum antibacterial activity, strong hemostatic effect, and mucosal adhesion properties. Hence, CS is often regarded as an ideal matrix material for preparing functional hydrogels 92. Numerous studies have confirmed that CS had multiple inherent advantages as follows 93,94: 1) Antibacterial performance: CS shows excellent antibacterial properties, which are attributed to the rich amino groups in its molecular chain. These amino groups can undergo protonation reactions under physiological conditions, thereby forming positively charged active sites on the material surface. Active sites can generate intense electrostatic interactions with inherently negatively charged groups on microbial cell membranes, thus leading to the destruction of cell membrane structure and triggering the death of microorganisms; 2) Tissue repair: CS has good biocompatibility, which promotes cell proliferation and regeneration during the tissue repair process; 3) Hemostatic effect: The cationic properties of CS can adsorb negatively charged platelets and activate the coagulation cascade reaction by aggregating red blood cells, thereby significantly enhancing the hemostatic efficacy. Notably, CS has poorer water solubility than other natural polymers (SA and HA), which limits CS's direct application in neutral/alkaline physiological environments. To address this limitation, we can prepare CS derivatives through chemical modification (carboxylation, acylation, quaternization, etc.) to enhance solubility 95. For instance, introducing a quaternary ammonium group at position C-6 could endow CS derivatives with excellent solubility (within the pH range of 3-13), which was mainly attributed to the strong hydrophilicity of the quaternary ammonium group 96. In addition to introducing the quaternary ammonium group, carboxymethyl chitosan (CMC), as a typical hydrophilic derivative, could maintain the biological activity of CS and significantly enhance water solubility 97. Currently, numerous studies are focusing on exploring the therapeutic effects of CS and its derivatives in diabetic wounds. For example, Deng *et al*. 98 prepared self-healing hydrogels by using quaternized chitosan (HACC) and dialdehyde-modified bacterial cellulose (DABC). The hydrogel possessed excellent biocompatibility and antibacterial properties (inhibiting the proliferation of *Escherichia coli* and *Staphylococcus aureus*), which could be applied to wound infection and repair. In another research, hydroxypropyl chitosan (HPC), caffeic acid functionalized chitosan (CCS), and oxidized dextran (ODex) were used as raw materials to prepare CS-based hydrogels (HPC/CCS/ ODEX-IGF1) with self-healing properties through dynamic imine bond cross-linking 99. The antibacterial rates of HPC/CCS/ODex-IGF1 against *Escherichia coli* and *Staphylococcus aureus* both exceeded 94%. Additionally, HPC/CCS/ ODEse-IGF1 also possessed anti-inflammatory and angiogenic-promoting properties, thereby promoting tissue regeneration and healing at the wound site (with a healing rate of approximately 98.4% on the 11th day), further suggesting that HPC/CCS/ Odese-IGF1 shows great application potential in the field of wound dressings. More importantly, the biological functions can be further enhanced by loading specific active molecules into CS hydrogels. Previous study has shown that the CS-based hydrogel loaded with basic fibroblast growth factor (bFGF) (BP/CS-bFGF) could significantly enhance cell migration and angiogenic activity 100. Besides, BP/CS-bFGF could up-regulate Ki67 expression and promote full-thickness wound healing in diabetic patients. Consequently, CS and its derivatives have demonstrated significant application value in the management of diabetic wounds. Nevertheless, the antibacterial activity of CS hydrogel is closely related to the pH of the wound environment. The pH of diabetic wounds is slightly alkaline, which leads to a decrease in the degree of amino acid protonation and a decline/loss of antibacterial activity. Additionally, a decrease in the degree of protonation may cause damage to the structural stability of the hydrogel in the hydrogel cross-linking systems involving amino groups (such as hydrogen bonds and electrostatic interactions). Interestingly, the structure and basic functions of SA and cellulose hydrogel are relatively stable in an alkaline environment. Therefore, we can utilize CS and SA/cellulose to construct a composite hydrogel system. This not only retains the inherent biological advantages of CS, but also enhances the stability of the hydrogel. Besides, the CS hydrogel system loaded with nanomaterials can achieve pH-independent and efficient antibacterial effects, which significantly improve the wide applicability of CS hydrogels in complex wound environments 101,102. In the future, we can further optimize the comprehensive performance of CS hydrogels, which will provide more effective solutions for the clinical treatment of diabetic wounds.

### 4.2. Sodium alginate

Sodium alginate (SA), as a natural anionic polysaccharide rich in hydroxyl (-OH) and carboxyl (-COOH) groups, is extracted from brown algae and bacteria. SA has been widely applied in the biomedical field due to its excellent biocompatibility and biodegradability 103. The basic structure of SA is composed of β-(1,4)-D-mannuronic acid (M unit) and α-L-glucuronic acid (G unit) arranged in different sequences 104. The physicochemical properties and biological activities of SA are related to the ratio of M units to G units (M/G) and the sequence distribution of its molecular chain. Numerous studies have demonstrated that SA with a high M content showed significant advantages in chronic wound healing, which was attributed to the fact that M unit could activate human monocytes and promote the secretion of pro-healing cytokines, thereby accelerating the wound repair process, implying that SA can become an ideal candidate material for chronic wound dressings 105,106. Notably, SA hydrogels have higher hygroscopicity and moisturizing capacity than other types of hydrogels. For instance, SA hydrogels can absorb a physiological saline solution equivalent to 15-17 times their weight. The data are significantly higher than those of CS hydrogels and cellulose hydrogels, which obviously reduce the risk of diabetic wound exudation. Additionally, SA hydrogels possess good moisturizing capabilities. Unlike the passive water retention of HA, the Ca^2+^ in the SA hydrogels can undergo ion exchange with the Na^+^ in the wound exudate and form a gel layer on the wound surface, thus maintaining a moist environment and promoting cell migration 107. More importantly, compared with CS, SA has milder gelation conditions and a faster gelation rate, which is conducive to SA demonstrating unique advantages in cell delivery and drug sustained-release 108. SA can interact with divalent cations (Ca^2+^ and Mg^2+^) through abundant carboxyl groups in the molecular chain, forming a stable cross-linking network with “egg box” properties, thus demonstrating its outstanding gel-forming ability 109. Besides, the -OH and -COOH groups present in the SA molecules can jointly form an environmentally-responsive three-dimensional network structure through dynamic covalent bonds (such as imine bonds) and various non-covalent interactions (hydrogen bonds, hydrophobic interactions, etc.) 110. In particular, -OH can be converted into active aldehyde groups, which can promote the formation of imine bonds and enhance the interfacial interaction with functional groups on the tissue surface 111. All in all, multiple cross-linking mechanisms can provide SA hydrogels with excellent self-healing ability and endow them with unique dynamic response properties, suggesting that SA hydrogels possess broad application prospects in the field of wound dressings.

SA hydrogels are mainly widely used in wound healing due to their moisturizing and promoting tissue regeneration functions. In recent years, numerous studies have focused on enhancing the degradation and biological functions (antibacterial, anti-inflammatory) of SA hydrogels via various cross-linking techniques and the introduction of bioactive compounds. Moreover, the development of stimulus-responsive and intelligent monitoring hydrogels has further expanded the application scenarios of SA hydrogels 112. For example, Yang *et al*. 113 designed a multifunctional hydrogel based on oxidized alginate (IA-PEI/OSA@CQD) that could be intelligently monitored. IA-PEI/OSA@CQD exhibited excellent swelling properties, antibacterial activity, and anti-inflammatory activity, which provided a favorable environment for diabetic wounds and promoted rapid wound healing. Additionally, IA-PEI/OSA@CQD could accurately measure the pH of diabetic wounds, which enabled intelligent monitoring of the healing process of diabetic wounds. In another study, the ingenious combination of pH/glucose dual-responsive hydrogels and Janus membranes enabled dynamic drug release and demonstrated good antibacterial properties (>98%), antioxidant properties, and hemostatic properties 114. Moreover, the Janus membrane could enhance the wound tear resistance of the hydrogel, which provided a new idea for the design of SA hydrogels with both precise drug release and high mechanical toughness. Notably, the SA hydrogels still have inherent limitations in practical applications. For example, single-component SA hydrogels usually rely on physical cross-linking, which results in insufficient mechanical strength and difficulty in regulating the degradation rate. Consequently, single-component SA hydrogels are prone to structural collapse in the dynamic environment of diabetic wounds and have difficulty maintaining long-term repair stability. To address this bottleneck, numerous studies have shifted from single-network optimization to multi-level structure design 115. A relevant study has revealed that alginate was molecularly modified with β-cyclodextrin (β-CD) and adamandane (Ad) to construct the ALG-CD: ALG-Ad host-guest cross-linked network, and Ca²⁺ was introduced for cross-linking to form a secondary network 116. The dual-network synergistic effect significantly enhanced the mechanical properties of the alginate gel. To effectively ameliorate the degradability of SA hydrogels, sodium iodate (NaIO₄) was used to oxidize the hydroxyl groups at C-2 and C-3 positions of the SA uronic acid unit and obtain oxidized sodium alginate (OSA) with a controllable oxidation degree 117. This chemical modification could maintain the inherent advantages of SA, including water solubility, biocompatibility, and low toxicity. Moreover, this modification could optimize the biodegradation performance and molecular flexibility of SA hydrogels, thus facilitating alignment of the hydrogel degradation behavior with the healing process of diabetic wounds. Therefore, the precise chemical modification and multi-level structure design can effectively overcome the inherent limitations of SA hydrogels, further suggesting that SA hydrogels have broad application prospects in the field of diabetes wound treatment.

### 4.3. Cellulose

Cellulose is a linear polysaccharide composed of D-glucose units linked by β-(1→4) glycosidic bonds. Cellulose, as the most abundant biopolymer in nature, can be efficiently extracted from various biomass resources (plant cell walls, extracellular secretions of microorganisms, etc.) 118,119,120. Compared with HA and CS cellulose has a wider source, lower cost, and more renewable, which is beneficial to the outstanding potential of cellulose in sustainability and scale application. Additionally, the cellulose molecular chain is rich in active hydroxyl groups (C2, C3, and C6 positions), which is conducive to the chemical modification of cellulose and the introduction of functional groups 121. The abundant hydroxyl groups can provide ideal cross-linking sites for the construction of hydrogels. These active groups can form physical cross-linking networks through intermolecular hydrogen bonds and covalently bind with functional cross-linking agents, thereby constructing stable three-dimensional hydrogel structures. Moreover, cellulose also has many inherent advantages, including excellent biocompatibility, controllable degradability, and environmental friendliness, thus demonstrating significant value in the selection of base materials for hydrogel preparation 122. Cellulose-based self-healing hydrogel (SHCH), as a new type of wound healing material, possesses some advantages as follows 123,124: 1) The surface tension and capillary action of SHCH can maintain its high water content, thereby creating moist environments conducive to cell proliferation and migration; 2) The inherent self-healing properties of SHCH is conducive to its spontaneous restoration of structural integrity after mechanical damage; 3) SHCH has excellent biocompatibility, tissue compliance, and breathability, which can effectively enhance the wound repair effect. These multifunctional SHCH systems offer innovative solutions to ameliorate clinical treatment outcomes and patient prognosis. Nevertheless, SHCH also has obvious limitations. For instance, natural cellulose is difficult to dissolve in water and most conventional solvents, which is attributed to highly crystalline molecular structure and strong hydrogen bond network. Hence, cellulose has a higher processing complexity than highly water-soluble polysaccharides (HA, SA, and starch). Additionally, the inherent rigidity of cellulose limits the mechanical properties of hydrogels, which particularly restricts the application of hydrogels with high flexibility in dynamic environments 125. To further optimize the performance of SHCH, we can functionalize the cellulose molecules through various chemical modifications. For instance, carboxymethyl cellulose (CMC) is a derivative obtained through carboxymethylation modification. CMC has water solubility, degradability, and biosafety, which is attributed to the abundant carboxyl groups on its molecular chains. Additionally, CMC can obviously shorten the gelation time and enhance the mechanical strength of hydrogels, thereby overcoming the shortcomings of slow curing and insufficient mechanical properties of traditional hydrogels. The rapid curing properties are conducive to the formation of stable hemostatic barriers by the hydrogels in a short time, thus significantly improving their hemostatic efficiency 126,127. Hydroxyethyl cellulose (HEC) is another important water-soluble cellulose derivative. HEC possesses high water solubility, biocompatibility, biodegradability, and non-immunogenicity. HEC can be further oxidized to form oxidized hydroxyethyl cellulose (OHEC) by using NaIO₄. The oxidized polymer hydrogel has a controllable gelation time, an ideal swelling rate, and excellent mechanical stability. Besides, OHEC hydrogel possesses a high water retention capacity, which can maintain moist environments on the wound surface for a long time. Furthermore, OHEC hydrogel exhibits low hemolysis rates, which is attributed to its favorable blood compatibility. These characteristics are conducive to OHEC hydrogel becoming a new type of material with both healing promotion and hemostatic functions, which can provide new ideas for the development of high-performance wound dressings 128. Currently, how to further optimize the mechanical performance of SHCH remains an important issue that needs to be urgently addressed 111,129. In addition to cellulose modification, optimization of cross-linking methods and introduction of dual network structures/nanocelluloses have attracted much attention, which can be attributed to their advantages, including high specific surface area and mechanical strength, and excellent biocompatibility 130. Nanocelluloses typically include cellulose nanofibers (CNF), cellulose nanocrystals (CNC), and bacterial nanocellulose (BCN) 131. Zhang *et al*. 132 investigated the mechanical properties and functional characteristics of CNF-based hydrogels. The introduction of CNF significantly enhanced the mechanical and compressive properties of the cellulose-based hydrogels. Additionally, CNF possessed high pointed surfaces and multiple hydroxyl groups, thus effectively improving the self-healing properties, degradability, and biocompatibility of hydrogels, which was crucial to promote wound healing. In another study, the mechanical strength of the self-healing hydrogel was increased by six times after the introduction of Ag hybrid bacterial cellulose nanofibers (Ag-BCN), which was attributed to the assembly of the three-dimensional network of BCN and hydrogel and the formation of intermolecular hydrogen bonds 133. Moreover, Ag-BCN could enhance the antibacterial activity of hydrogels. Accordingly, the mechanical properties and biological functions of SHCH could be optimized by introducing nanocellulose. Currently, SHCH still faces several bottlenecks in the application of diabetic wounds as follows: 1) SHCH lacks hemostatic function and angiogenic activity. Consequently, SHCH usually needs to be modified in combination/loaded with active factors (VEGF and bFGF) to enhance biological function; 2) The metabolic pathway of nanocellulose in the body is still unclear, which increases the risk of long-term biological safety assessment of hydrogels and restricts the clinical transformation process; 3) There is a mechanical mismatch between the rigid structure of SHCH and the dynamic microenvironment of diabetic wounds. An excessively high elastic modulus can limit the adhesion of hydrogels to irregular wounds, disrupt the stability of interface contact, and hinder wound healing. Future studies should focus on constructing a multifunctional synergy system to balance mechanical properties, biological activity, and degradation behavior, thereby meeting the complex requirements of diabetic wound healing.

### 4.4. Hyaluronic acid

HA is composed of repeating disaccharide units of D-glucuronic acid and N-acetyl-D-glucosamine. These units are linked by β-1,3 and β-1,4 glycosidic bonds. HA, as the main component of ECM, is widely present in human connective tissues and body fluids. Numerous studies have shown that HA has many advantages in the treatment of diabetic wounds as follows 111,134,135: 1) HA has good biocompatibility and moisturizing properties, which is conducive to covering irregular wound surfaces, maintaining a moist environment at the wound site, promoting cell proliferation and migration, and tissue regeneration; 2) HA can bind to the CD44 receptor on M1 macrophages through its own structural characteristics, promoting the polarization of macrophages from M1 type to M2 type, and alleviating the continuous inflammatory response at the wound site; 3) HA can interact with various cell surface receptors and HA-binding proteins, thus mediating cell behavior and regulating ECM function; 4) HA has high hygroscopicity, which is mainly attributed to the presence of numerous hydrophilic groups (such as hydroxyl and carboxyl groups) in its chemical structure. The hygroscopic properties can maintain a moist environment for diabetic wounds, prevent scab formation, and promote the exchange of nutrients and oxygen, thereby creating favorable conditions for wound healing. Moreover, the hygroscopic properties of HA can also remove necrotic tissues and exudates on the wound surface, keep the wound clean, and reduce the risk of infection. Consequently, HA is widely used in various tissue repair and wound dressings due to its extensive biological effects and multiple excellent properties 136,137. However, HA can be rapidly degraded through enzymatic reactions under physiological conditions. Hydrogels formed by natural HA usually have poor mechanical properties and a short residence time* in vivo*. Additionally, the high molecular weight of HA has limited solubility, which further restricts the application of HA in the medical field. Therefore, the modification of HA is crucial for enhancing the biological activity and mechanical properties of hydrogels 138. Previous study has shown that we can improve the functional properties of HA through the following specific approaches 139: 1) Sulfuration modification can enhance the mucosal adhesion of HA and form a disulfide bond cross-linking network, thereby significantly improving the enzymatic resistance and cell barrier penetration ability; 2) The modification of hydroxyl groups or the combination with nanomaterials can effectively enhance the mechanical properties of HA hydrogels; 3) We can introduce hydrophobic groups (cholesterol, fatty acids) to construct amphiphilic HA derivatives, which enhances the drug delivery efficiency and targeted delivery ability of HA hydrogels for hydrophobic drugs; 4) We can utilize active functional groups to structurally modify and cross-link HA, which is conducive to expanding their application in fields, such as skin repair and tissue engineering. At present, numerous studies are focusing on exploring the therapeutic effects of HA-based self-healing hydrogels in the field of diabetic wounds. For example, Chen *et al*. 140 prepared novel dynamic HA-based self-healing hydrogels by combining borates and coordination chemistry. HA-based self-healing hydrogels possessed multiple response characteristics. HA-based self-healing hydrogels displayed reactive degradation of diabetic wounds and controllable H_2_S release. Additionally, HA-based self-healing hydrogels could effectively regulate macrophage polarization (from M1 to M2 type) through the synergistic effect of ROS clearance, H₂S release, and Zn²⁺ regulation. Furthermore, HA-based self-healing hydrogels could simultaneously regulate angiogenesis signaling pathways and promote the formation of mature blood vessels, epithelial regeneration, and collagen deposition, further suggesting that HA-based self-healing hydrogels have broad applications in the field of diabetic wound repair. In another study, a multifunctional HA hydrogel (ODF-Met) was successfully prepared using oxidized hyaluronic acid as the raw material through dopamine grafting, metformin loading, and Fe^3+^ coordination cross-linking 141. The introduction of dopamine overcame the poor adhesion defect of natural HA. Additionally, the phenolic-metal coordination network endowed ODF-Met with photothermal antibacterial activity. Furthermore, metformin was combined through dynamic imine bonds, which facilitated the controlled release of metformin in diabetic wounds. Hence, dynamic cross-linking and multifunctional HA hydrogels provide an innovative treatment approach for diabetic wound repair, further suggesting that HA hydrogels have strong potential for clinical application. Notably, HA hydrogels still encounter some challenges in practical applications as follows 142,143: 1) The structural stability of HA hydrogels is poor in acidic environments, which leads to an overly rapid drug release rate; 2) Various enzymes in wound exudates can degrade the HA hydrogel network structure, which shortens the duration of the hydrogel's effect; 3) Species differences between experimental animals and human patients may result in poor gel formation time, drug release behavior, and degradation rate, thereby affecting the clinical treatment outcome. In the future, we should focus on the following improvement strategies: 1) Design intelligent responsive HA hydrogels using dynamic covalent bonds to achieve drug release that adapts to the microenvironment; 2) Explore low-cost green extraction and modification processes to promote clinical translation; 3) Build humanized organoid models to accurately evaluate the* in vivo* behavior of HA hydrogels, and realize personalized and large-scale applications of HA hydrogels in diabetic wounds.

### 4.5. Other polysaccharides

In addition to CS, SA, cellulose, and HA, polysaccharides, including chondroitin sulfate, guar gum (GG), dextran (Dex), and starch (ST), have demonstrated significant application value in the field of wound dressings due to their unique biological characteristics and functional diversity.

Chondroitin sulfate, as an important component of ECM, can be an ideal material for preventing wound infection and promoting wound healing, which is attributed to its excellent antibacterial, anti-inflammatory, and antioxidant activities 144,145. Unfortunately, chondroitin sulfate has lower molecular weights than other polysaccharides, thus leading to its poor mechanical properties and faster degradation rates 146. For this reason, researchers can optimize the mechanical properties and degradation rate of chondroitin sulfate hydrogels through strategies, such as chemical modification or construction of composite hydrogels. In terms of biological functions, chondroitin sulfate can significantly up-regulate the proliferation activity of fibroblasts, thus activating the cascade reaction of wound healing. In addition, chondroitin sulfate has also demonstrated the ability to promote tissue regeneration in various wound models (diabetic wounds, partial thickness, and full-thickness skin defects). Furthermore, chondroitin sulfate can interact with various growth factors (cytokines, TGF-β, and adhesion molecules), chemokines, and lipoproteins, thereby regulating the wound healing process 147.

GG, as a naturally occurring nonionic polymer, possesses excellent hydrophilicity, biocompatibility, and degradability 148. Relevant research has shown that mannose residues in GG could induce macrophages to transform from M1 type to M2 type, thereby reducing inflammatory responses and improving the microenvironment of the wound surface 149. Besides, GG is rich in hydroxyl groups, which can form hydrogen bond networks between/within molecules, thus endowing GG with excellent biocompatibility and gel-forming properties 150. Nevertheless, GG hydrogels lack antimicrobial activity, which limits their application in the treatment of wound infections 151. To overcome this limitation, GG/ its derivatives are usually combined with other polysaccharides/materials with antibacterial activity to enhance the antibacterial activity of GG hydrogels. For instance, CG-Cu composite hydrogels were prepared by taking advantage of the broad-spectrum antibacterial activity of Cu^2+^ by combining Cu^2+^ with CG 152. CG-Cu composite hydrogels showed excellent antibacterial activity. Moreover, the CG-Cu composite hydrogel could form a protective barrier highly adapted to the morphology of the wound through in situ injection. This not only effectively reduced the risk of wound infection, but also was conducive to promoting the regeneration and repair of the full-thickness skin tissue.

Dex, as a natural polysaccharide, is widely present in plants and microorganisms. Dex has outstanding biocompatibility, degradability, and clinical safety 153. Dex can effectively promote wound healing through multiple pathways as follows 129: 1) Dex can remove exudate and metabolic waste from the wound surface, thereby improving the microenvironment for wound repair; 2) Dex can promote angiogenesis, thus preventing tissue ischemic injury; 3) Dex can promote the formation of granulation tissue and accelerate the wound healing process; 4) Dex can stimulate collagen deposition, thereby influencing the tissue remodeling process. These characteristics are conducive to Dex playing an important role in wound repair. Currently, Dex hydrogels have become research hotspots due to their unique swelling properties and mechanical strength. Dex hydrogels prepared by the physical cross-linking method possessed excellent drug loading and controlled release capabilities. Additionally, Dex hydrogels exhibit various swelling behaviors and crystallization characteristics under different pH conditions, which enable the controllable release of bioactive substances, further confirming that Dex hydrogels show promising application prospects in the field of wound dressings 154.

ST shows good biodegradability, cost-effectiveness, and biosafety. Accordingly, ST displays broad application prospects in the biomedical field (tissue engineering, wound dressings, and drug delivery) 155. ST can be used as an ideal matrix material for developing hydrogels with high swelling properties, which are attributed to its excellent hydrophilicity 156. ST hydrogels can achieve rapid wound hemostasis by relying on their high water absorption, strong swelling, and adhesion in the stage of wound hemostasis. In addition, proteins and trace elements (potassium and magnesium) contained in ST provide favorable conditions for cell metabolism and proliferation 13. Nevertheless, low biocompatibility and mechanical properties of ST limit its application in wound healing. To overcome these deficiencies, currently, many strategies, such as oxidation modification, loading nanoparticles, and mixing with other polymers, are mainly adopted to optimize the biological properties and mechanical characteristics of ST hydrogels, which are conducive to expanding their application in the field of wound repair 157,158. Table 2 shows the chemical structures, sources, and wound healing mechanisms of different polysaccharides.

## 5. The design strategy of PSHs

Self-healing hydrogels have significant value in the field of wound healing, which is attributed to their ability to automatically restore structural and functional integrity after damage, thereby maintaining the stability of the wound microenvironment and promoting tissue regeneration. In recent years, PSHs have attracted much attention in the treatment of chronic and complex wounds due to their outstanding biocompatibility and structural designability. PSHs can effectively prevent irreversible damage during the application process and extend the service life of the materials. Currently, the design strategies of PSHs mainly rely on two types of dynamic interactions. Physical non-covalent interactions include hydrogen bonds, hydrophobic interactions, host-guest interactions, electrostatic interactions, and metal coordination. These reversible physical cross-linking points endow hydrogels with the ability to self-healing rapidly. In addition, dynamic chemical covalent bonds include imine bonds, amidoxime bonds, borate ester bonds, and disulfide bonds. PSHs achieve network reconfiguration through the reversible cleavage and rearrangement of these dynamic chemical bonds. Importantly, the synergy of these two mechanisms can optimize the mechanical properties, self-healing efficiency, and microenvironment responsiveness of the hydrogel. Consequently, a deep understanding of the construction principles of the two types of dynamic interactions is crucial for designing high-performance PSHs. In this section, we focus on the design strategies for PSHs based on physical non-covalent interactions and dynamic chemical covalent bonds.

### 5.1. Physical non-covalent interactions

#### 5.1.1. Hydrogen bonds

**Figure 3:**
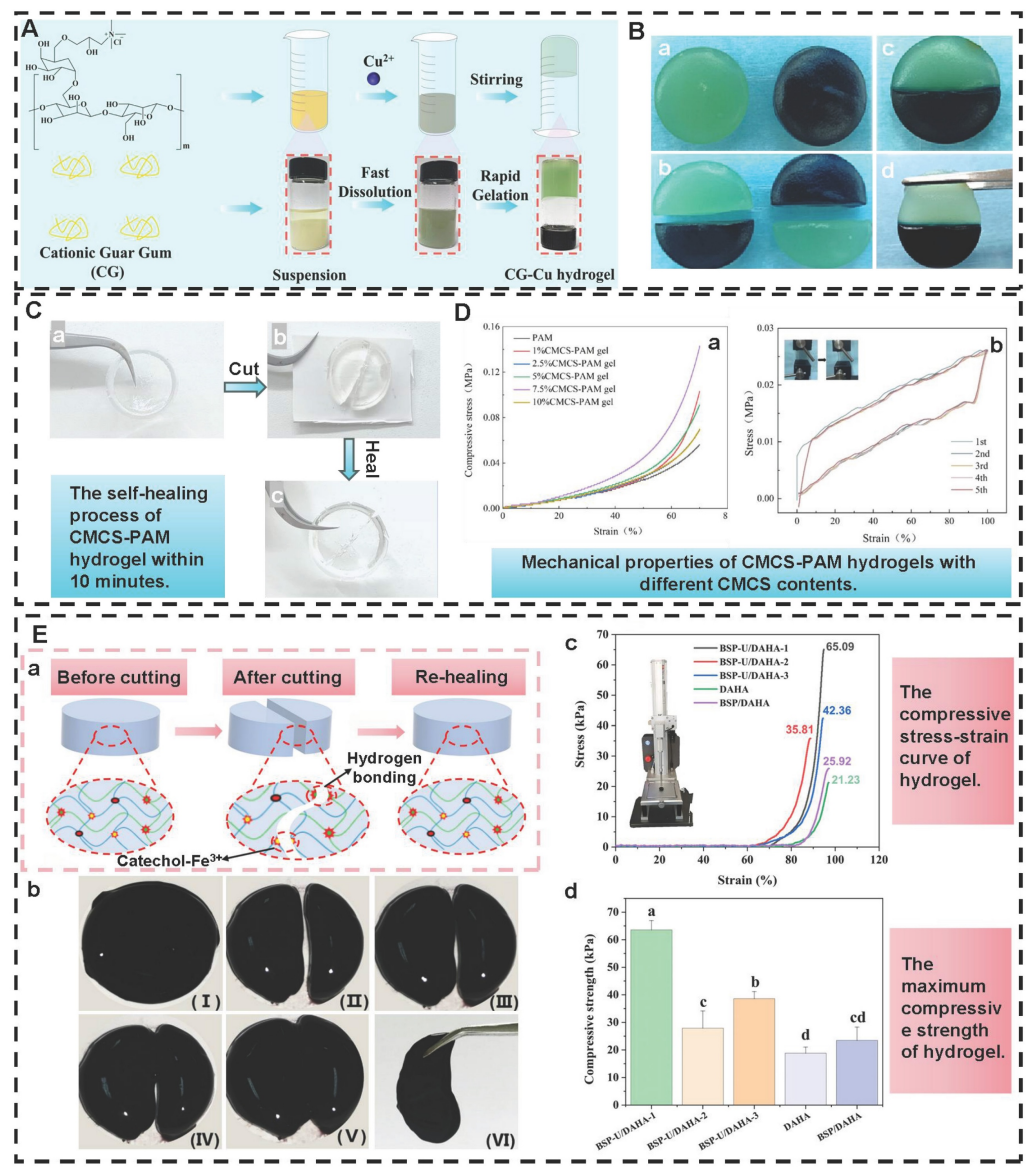
PSHs formed by hydrogen bonds. (A) The preparation process of CG-Cu hydrogel. (B): Figure (a) to (d) show the self-healing behavior of CG-Cu1 hydrogel. Adapted with permission from 152, copyright 2023 Elsevier. (C): Figure (a) to (c) show the self-healing process of CMCS-PAM hydrogel. (D): (a) Compressive stress-strain curves of CMCS-PAM hydrogels with various CMCS contents. (b) Five cycles of continuous load-and-unload tensile tests were conducted on 5wt% CMCS-PAM hydrogel at 100% strain. Adapted with permission from 166, copyright 2024 Elsevier. (E): (a) and (b) present the macroscopic self-healing schematic diagram and the actual situation schematic diagram of the BSP-U/DAHA-1 hydrogel, respectively. (c) and (d) represent the compressive stress-strain curve and the maximum compressive strength of the hydrogel, respectively. Adapted with permission from 167, copyright 2023 Elsevier.

Hydrogen bonds, as typical physical interactions, have weaker bond energy than covalent bonds. Nevertheless, coordinated hydrogen bond interactions can enhance the bonding strength, thereby promoting the formation of hydrogels 159. The H atoms exhibit partial positive charge characteristics when strongly electronegative atoms (N, O, and F) combine with H atoms, while the electronegative atoms carry partial negative charges 160. This charge separation effect is conducive to the formation of electrostatic interactions between H donors and H acceptors. Intermolecular hydrogen bonds can be directionally attracted by hydrogen donors and electronegative atom acceptors, thereby establishing dynamic, reversible, and cross-linked networks between different polymer chains 161. Additionally, the formation and splitting of hydrogen bonds occur rapidly, which is conducive to the quick healing of hydrogels cross-linked by hydrogen bonds after damage 34. Natural polysaccharides (HA, CS, SA, etc.) are rich in hydrogen bond donors and acceptors (hydroxyl, carboxyl, amino, etc.), which are conducive to the formation of self-healing hydrogels through the synergistic effect of multiple hydrogen bonds 162. Wang *et al*. 152 investigated the self-healing properties of CG-based hydrogel with hydrogen bond cross-linking (Figure 3A). The cut hydrogel material reformed into a complete gel within 1 h, further demonstrating that this hydrogel has excellent self-healing properties (Figure 3B). Moreover, the repaired hydrogel could still maintain structural integrity when tensile strain exceeded 200%, further suggesting that the hydrogel constructed by a hydrogen bond cross-linking mechanism display great application potential in the field of wound dressings. Nevertheless, the stability of hydrogen bonds in complex diabetic wounds still faces many challenges. For instance, a weakly alkaline environment can disrupt the proton balance and interfere with hydrogen bond formation. Additionally, high-concentration glucose can compete with the hydrogel network for hydrogen bond binding sites due to its multi-hydroxyl structure, which leads to a decrease in cross-linking density. Furthermore, high concentrations of ROS can directly destroy hydrogen bond donors/receivers via oxidizing the functional groups (hydroxyl and amino) of polysaccharide chains 163. Consequently, hydrogels solely relying on hydrogen bond cross-linking have limitations, such as unstable network structure and reduced self-repair efficiency. To this end, the current researches mainly enhance the mechanical strength of the hydrogel by constructing a dual-network (DN) structure or introducing multiple hydrogen bond cross-linking 164,165. For example, Ma *et al*. 166 demonstrated that the DN hydrogel (CMCS/PAM) prepared from carboxymethyl chitosan (CMCS) and polyacrylamide (PAM) had higher mechanical properties than the single-network PAM hydrogel, which was attributed to the DN structure's ability to facilitate dispersion and transfer of external forces across the dual-network architecture, thereby significantly enhancing the hydrogel's mechanical strength (Figure 3D). Additionally, CMCS/PAM hydrogels showed excellent self-healing performance (Figure 3C). Similarly, the physical DN hydrogels of natural polysaccharides (BSP-U/DAHA-1) had a maximum compressive stress (63.57% ± 3.38%) (Figure 3E) 167. This was attributed to the fact that BSP-U/DAHA-1 significantly enhanced its mechanical strength through stable physical DN cross-linking. Besides, urea-pyrimidinone, as a supramolecular self-assembly motif, could significantly improve the mechanical properties of BSP-U/DAHA-1 by forming quadruple hydrogen bonds for cross-linking. Therefore, DN structures and multiple hydrogen bond cross-linking provide effective solutions for optimizing the mechanical properties of PSHs.

Notably, increasing the hydrogen bond density/introducing strong interactions may limit the mobility of polymer chains and the rate of dynamic chain exchange, thus leading to a decline in self-healing efficiency. Current researches mainly employ hydrogen bonds and other dynamic covalent bonds (disulfide bonds and borate ester bonds) to combine and construct hybrid cross-linking networks, thereby balancing the mechanical strength and self-healing efficiency of the hydrogel 168. For instance, previous study has prepared dual-cross-linked nanocellulose hydrogels using a gradient temperature control strategy 169. The hydrogel showed excellent mechanical strength (breakage strain > 1000%) and rapid self-healing performance (recovery within 8 s), which was attributed to the energy dissipation effect of hydrogen bonds and the dynamic reorganization of the borate ester bond network. Hence, the hybrid cross-linking network provides an important strategy for balancing the mechanical strength and self-healing efficiency of hydrogels. However, this strategy still has certain limitations as follows: 1) The proportion of dynamic bonds is difficult to be precisely controlled; 2) The differential response of hybrid bonds in the diabetic microenvironment may lead to the instability of the network structure. Therefore, future studies should focus on the following: 1) Constructing precise control methods for dynamic bond ratios to achieve the synergistic optimization of mechanical properties and self-healing efficiency; 2) Developing intelligent hydrogen bond networks with environmental responsiveness to improve the adaptability of PSHs in the dynamic microenvironment of diabetic wounds.

#### 5.1.2. Hydrophobic interactions

**Figure 4:**
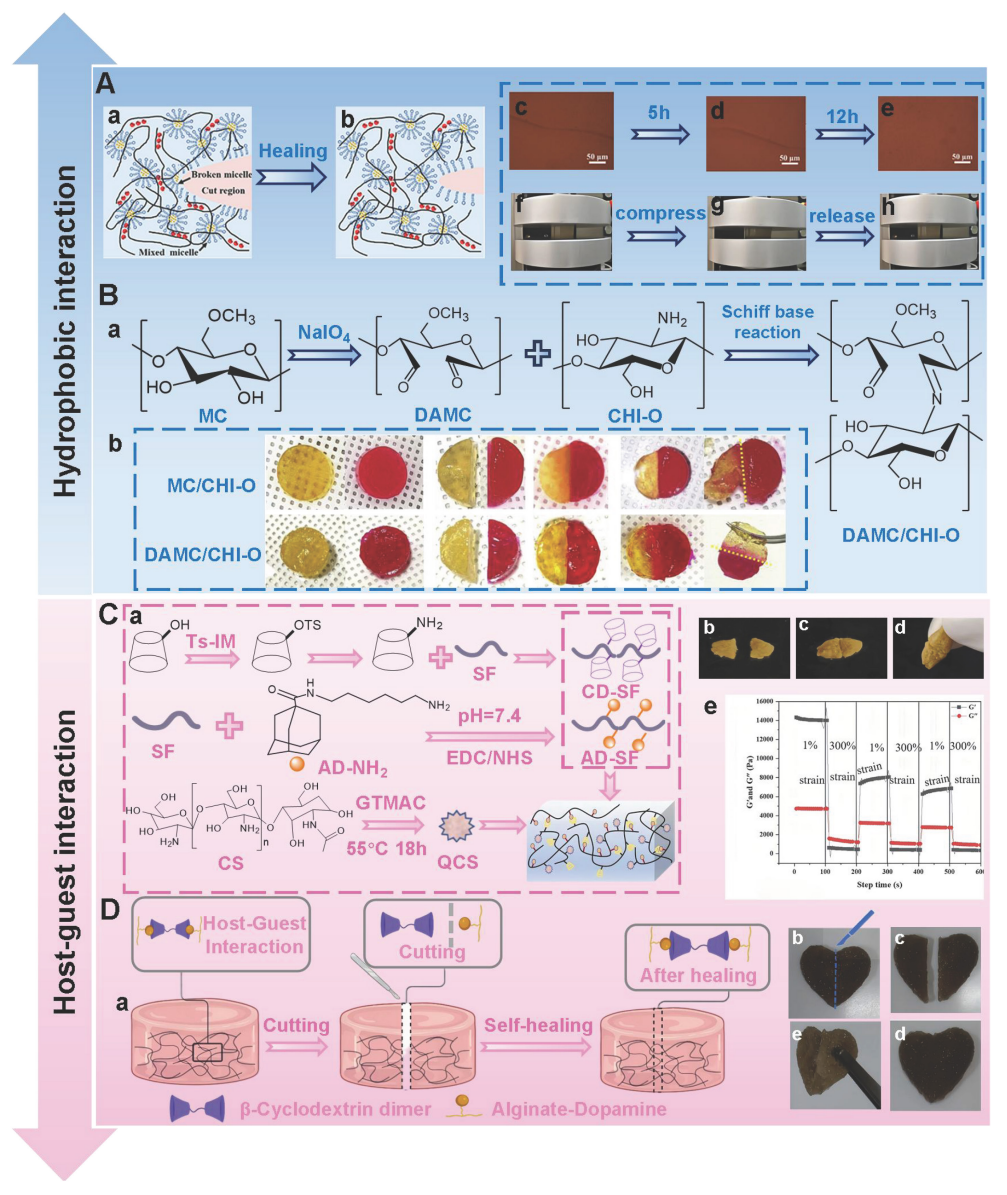
PSHs formed through hydrophobic interactions or host-guest interactions. (A): (a) and (b) represent the schematic diagrams of the gel regions of SA-RFS-C18M-30 HA hydrogel before and after healing. (c), (d), and (e) are the optical images of the self-healing process of SA-RFS-C18M-30 HA hydrogel. (f), (g), and (h) indicate that the hydrogel rapidly returns to its original shape after undergoing a series of compressive deformations and subsequent force release. Adapted with permission from 175, copyright 2020 ACS Publications. (B): (a) The preparation process of DAMC-CHI-O hydrogel. (b) The self-healing processes of MC/CHI-O and DAMC/CHI-O. Adapted with permission from 178, copyright 2021 Elsevier. (C): (a) Schematic diagram of QCA hydrogel preparation. (b), (c), and (d) represent the self-healing process of the sample QCA20 hydrogel. (e) The self-healing performance of the hydrogel was tested by a rheometer (QCA20 hydrogel was subjected to alternating strain ranging from 1% to 300%). Adapted with permission from 188, copyright 2024 Elsevier. (D): (a) to (e) exhibit the self-healing ability of AMDB-4 HG hydrogel. Adapted with permission from 190, copyright 2024 Elsevier.

Hydrophobic interactions refer to the phenomenon where hydrophobic groups aggregate to avoid the aqueous phase 170. Hydrophobic interactions occur between amphiphilic polymer chains that possess both hydrophilic and hydrophobic groups, which can be fine-tuned by altering the shape of the hydrophobic region and the number of hydrophobic groups 170,171. Compared with short-range interactions (hydrogen bonds and ionic bonds), hydrophobic interactions have more significant long-range characteristics, which is attributed to the fact that the proximity of hydrophobic molecules can trigger the rearrangement of the hydrogen bond network of surrounding water molecules, thereby generating synergistic effects 172. This unique mechanism of action not only extends the effective interaction distance of hydrophobic interactions, but also significantly improves the tensile and mechanical properties of hydrogels through dynamic, reversible, and cross-linked networks. Polysaccharide molecules can introduce hydrophobic groups onto their molecular chains through chemical modification. These hydrophobic groups spontaneously aggregate and form dynamic and reversible three-dimensional network structures in water environments, thereby endowing hydrogels with good self-healing properties 173. For instance, Fredrick *et al*. 174 grafted hydrophobic dodecanamine onto carboxymethyl cellulose to prepare hydrogels. The hydrogels were able to maintain structural integrity and achieve rapid self-healing even under a 200% strain condition. Similarly, Meng *et al*. 175 introduced hydrophobic monomer stearyl methacrylate (C18M) and amphiphilic regenerated silk fibroin (RSF) solution into the alginate ion network to prepare self-healing hydrogels. The dynamic modulus of the hydrogels could be restored after each cycle, and the fractured hydrogels almost completely healed within 12 h, further suggesting that the hydrogels have good self-healing ability (Figure 4A). Additionally, the mechanical properties of the hydrogels were positively correlated with the concentration of C18M within a certain concentration range (0-30 mM). Nevertheless, the concentration of C18M exceeding 30 mM/L could lead to a decline in the mechanical properties of the hydrogels. Therefore, adding appropriate concentrations of hydrophobic monomers provides an important reference for the performance regulation of hydrophobic association networks. In addition to mechanical reinforcement, hydrophobic interactions can also effectively improve the swelling resistance of hydrogels 176. Previous study has shown that non-swelling PTL-CS hydrogels could be constructed by grafting short hydrophobic alkyl chains (pentenyl groups) onto CS molecular chains through N-acylation reactions 177. The pentenyl groups in PTL-CS hydrogels could be cross-linked by ultraviolet light to form hydrophobic chains, thereby enhancing the hydrophobic effect to keep hydrogels in a non-swollen state. This not only avoided the damage to surrounding tissues and secondary injuries caused by the expansion of hydrogels, but also further improved the biocompatibility and safety of hydrogels. Nonetheless, single hydrophobic interactions face challenges of decreased network stability and reduced self-healing efficiency in the diabetic wound micro-environment (weakly alkaline, high glucose, and high ROS). For example, high concentrations of ROS may oxidize the hydrophobic groups (unsaturated alkyl chains)/amphiphilic polymers' connection bonds, which lead to the disruption of the hydrophobic region structure. Consequently, single hydrophobic interactions are difficult to form hydrogel networks with a stable structure 102,164. To effectively address the issue, Yeo *et al*. 178 prepared double-cross-linked self-healing hydrogels (DAMC/CHI-O) based on hydrophobic interactions and dynamic imine bonds using methylcellulose (MC) and chitosan oligomers (CHI-O) as raw materials. DAMC/CHI-O hydrogels had better self-healing performance than MC/CHI-O hydrogels (involving only hydrophobic interactions) (Figure 4B). In another study, DN hydrogels based on hydrophobic interactions and ionic cross-linking had outstanding self-healing properties and stability 179. The hydrogels exhibited more excellent mechanical properties than other groups (fracture stress of 1160.60 kPa, fracture strain of 2604%, elastic modulus of 71.79 kPa, and toughness of 14.20 MJ m^-3^). The above-mentioned researches provide new ideas for enhancing the stability of self-healing hydrogels based on hydrophobic interactions. Previous report has also found that the existence and intensity of hydrophobic interactions could affect drug release behavior 180. Hydrophobic drugs can form hydrophobic interactions with hydrophobic regions in the hydrogel matrix, thereby achieving controllable and sustainable drug release. Hydrophobic interactions can effectively inhibit the burst release of drugs, which provides favorable conditions for the construction of sustained-release systems, thus maximizing therapeutic efficiency and reducing the toxicity of drugs to healthy tissues 181.

Notably, hydrophobic interaction hydrogels still have bottlenecks in terms of equilibrium strength and self-healing properties as follows: 1) There is a lack of quantitative standards for “moderate hydrophobic modification”. Too low hydrophobicity leads to a fragile hydrogel network, while too high hydrophobicity may cause restricted chain segment movement and reduced self-healing efficiency; 2) The synergistic mechanisms of hydrophobic interactions and other dynamic bonds (imine bonds and ionic bonds) in the hybrid network are still unclear. In the future, we should establish a quantitative structure-activity relationship between the degree of hydrophobic modification and gel properties, and determine the optimal hydrophobicity for specific application scenarios (diabetic wounds). Additionally, we need to precisely control the ratios and spatial distributions of hydrophobic interactions and other dynamic bonds, which can establish a hybrid hydrogel system with a stable structure and appropriate functionality.

#### 5.1.3. Host-guest interactions

Host-guest interactions refer to the supramolecular assembly process based on molecular recognition mechanisms. The essence of host-guest interactions is that guest molecules specifically bind to macrocyclic hosts through non-covalent bonds (hydrogen bonds, van der Waals forces, hydrophobic interactions, and electrostatic interactions). These structurally complementary interactions can form supramolecular complexes with specific physicochemical properties and functions 182,183. Polymer networks based on host-guest interactions exhibit excellent self-healing performance, which is attributed to their dynamic reversibility 182. Crown ether, cyclodextrin (CD), and cucurbituride are the main host molecular compounds. In addition, adamantane (AD), cholic acid, ferrocene, and azobenzene are typical guest molecules 183,184. CD is the most widely studied supramolecular host in the biomedical field 185. Natural CDs include α-CD, β-CD, and γ-CD. CD possesses a unique cavity, which is conducive to the formation of an inclusion complex with various hydrophobic guest molecules. Among them, the cavity size of β-CD is more suitable for inclusion body complexation of various guest molecules with biological and pharmacological properties 186,187. Compared with other guest molecules, AD has a higher binding constant (Ka) with β-CD, thus enabling the formation of more stable inclusion complexes 116. In recent years, PSHs constructed based on the host-guest interactions of β-CD and AD have become a research hotspot. For example, Guo *et al*. 188 prepared self-healing hydrogels (QCA) via quaternary ammonium chitosan (QCS), β-CD-modified silk fibroin (CD-SF), and AD-modified silk fibroin (AD-SF). The fractured QCA hydrogels possessed favorable self-healing performance, which was attributed to the existence of host-guest interactions in the hydrogels (Figure 4C). Moreover, the cross-linking density between AD-SF and CD-SF was positively correlated with the self-healing performance of QCA. Consequently, this study provides design ideas for the preparation of PSHs that promote wound healing. Notably, the cross-linking ability of CD still has obvious limitations, which pose a significant challenge for the development of high-strength and highly self-healing hydrogels based on host-guest interactions 189. To overcome these limitations, numerous studies have introduced poly-cyclodextrin (PCD), β-cyclodextrin dimers, and β-cyclodextrin trimers to increase the host-guest interaction sites and enhance the cross-linking density 190. Previous study has shown that the introduction of PCD significantly enhanced the self-healing ability and mechanical properties of the hydrogel, which was attributed to the fact that PCD could provide more host sites and binding opportunities, thus forming a stable network structure 191. Similarly, the alginate-based hydrogels incorporating β-cyclodextrin dimers (β-CDsD) had excellent self-healing properties and mechanical properties 190. The fractured hydrogels completely healed within 5 min, confirming their excellent self-healing capacity (Figure 4D). Additionally, the hydrogels possessed outstanding mechanical properties, which was attributed to the fact that the β-CDsD core cross-linking agent could form more host-guest interactions with the polymer chains. In addition to the regulation of self-healing performance and mechanical properties, the intelligent drug delivery system based on host-guest interactions provides a new strategy for the treatment of diabetic wounds. We can utilize *in vitro* physical stimuli (light and temperature) and stimuli from the microenvironment of diabetic wounds (oxidative stress, pH, and enzymes) to exert the dynamic response properties of the host-guest interactions 192,193. For example, Zhao *et al*. 194 prepared photoresponsive supramolecular polysaccharide hydrogels through the host-guest interactions between azobenzenes and HA-coupled β-CD groups. The hydrogels could achieve rapid release of EGF under ultraviolet irradiation. Additionally, EGF in the hydrogels was released slowly under visible light irradiation. Accordingly, the release rate of EGF in hydrogels could be easily regulated by alternating ultraviolet light and ambient visible light irradiation. In another study, HA-based hydrogels based on host-guest interactions had ROS response characteristics 195. The hydrogels could adjust their internal cross-linked structure and achieve partial dissociation under oxidative stimulation, which was conducive to the exposure and delivery of self-assembled emodin. Besides, the diabetic wounds treated with the hydrogels successfully transitioned from the inflammatory phase to the proliferative phase, thus promoting the entry of chronic wounds into the normal repair stage. Similarly, HA-based hydrogels modified via cyclodextrin (HA-CD) and adamantane (Ad-HA) exhibited dual response characteristics of MMP/ROS 196. The hydrogel could trigger the controlled release of antimicrobial peptides after being simultaneously exposed to the MMP and ROS environment, which enhanced the sustained-release properties and biological safety of the drug. Hence, the dynamic response properties of host-guest interactions lay a crucial foundation for the construction of microenvironment-adapted diabetic wound dressings. Nevertheless, PSHs based on host-guest interactions still face many challenges in the treatment of diabetic wounds: 1) The influence mechanism of the pathological microenvironment on the affinity of host-guest binding remains unclear; 2) The spatial distribution and binding site density of multi-level host networks are difficult to precisely control, which may lead to an imbalance in mechanical properties and self-healing efficiency. Consequently, future research should focus on: 1) Revealing the influence laws of diabetic pathological factors on the host-guest binding constant; 2) Developing novel host-guest pairs with adjustable affinity to achieve coordinated optimization of cross-linking density and dynamic response.

#### 5.1.4. Electrostatic interactions

**Figure 5:**
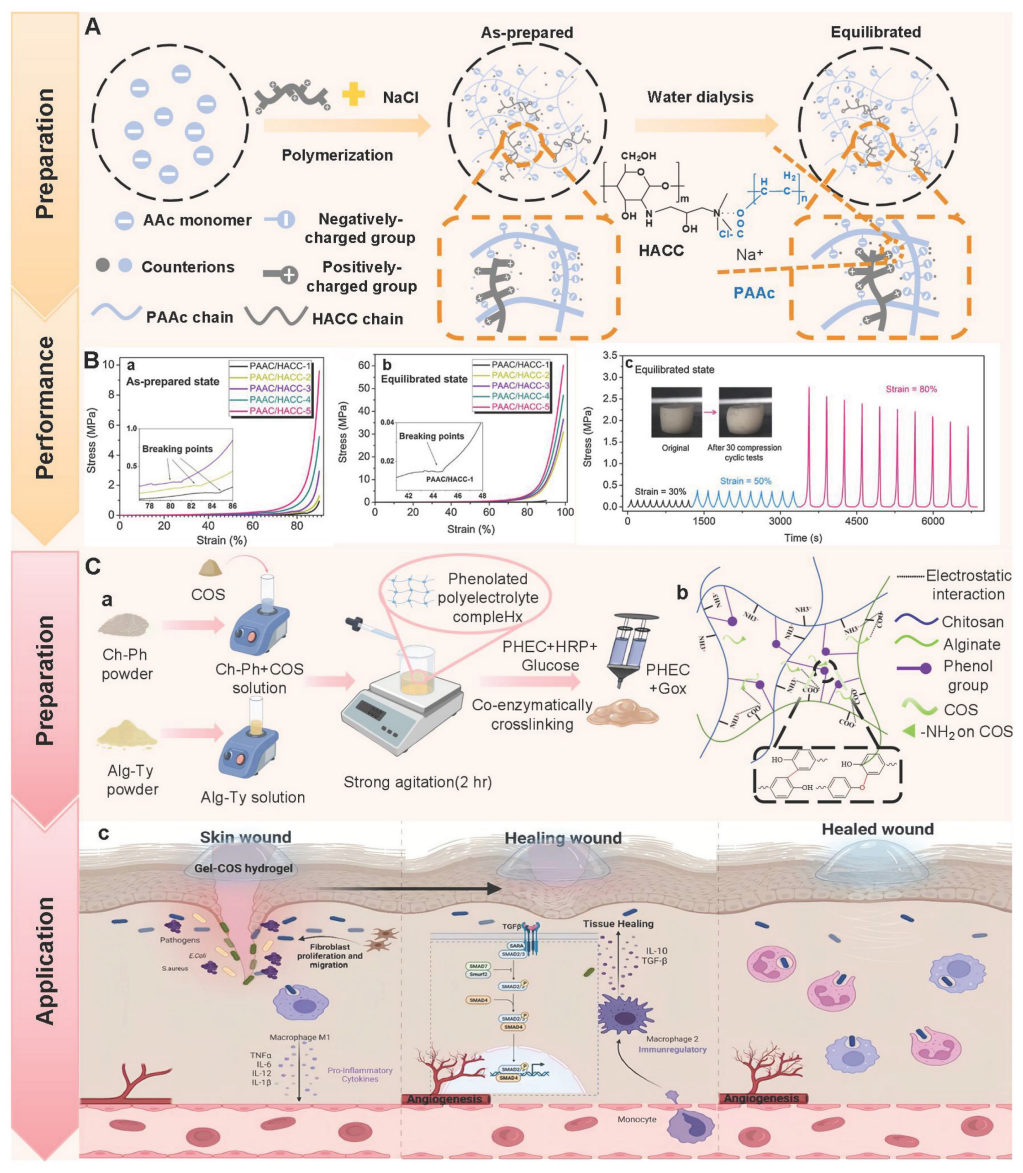
PSH based on electrostatic interactions and their functional properties. (A) Schematic diagram of the preparation method and network structure of PAAc/HACC hydrogel. (B): Schematic diagram of mechanical performance evaluation for PAAc/HACC. (a) and (b) are the compressive stress-strain curves of PAAc/HACC in the prepared and equilibrium states, respectively. (c) Schematic diagram of the stress-time compression results of PAAc/HACC-4 hydrogel in equilibrium state. Adapted with permission from 205, copyright 2018 Elsevier. (C): (a) Schematic diagram of the Gel-COS preparation process. (b) Interactions generated during the preparation of the Gel-COS hydrogel. (c) Mechanism of Gel-COS hydrogel in promoting wound healing. Adapted with permission from 202, copyright 2022 Elsevier.

Electrostatic interactions stem from the electrostatic attraction between monomers or polymers with opposite charges, which provides the main driving force for the formation of hydrogels 197,198. In the three-dimensional network structure of hydrogels, the migration behavior of uncross-linked parts and the dynamic movement of free ions jointly endow the electrostatic interaction with significant reversible characteristics, which largely determine the self-healing performance of hydrogel systems 102. Additionally, hydrogels prepared based on electrostatic interactions can alleviate the residue contamination and cytotoxicity problems caused by chemical cross-linking, thereby enhancing the safety and biocompatibility of hydrogels 199. Currently, electrostatic interactions are mainly involved in the gelation process of alginate, CS, and low-methoxy pectin (Lm-pectin) 200. Song *et al*. 201 prepared self-healing hydrogels through a one-step “freeze-thaw” method using cordycepin (CY) and CS as raw materials. The hydrogels had favorable self-healing behavior and shear-thinning characteristics. Moreover, the electrostatic interactions between CS and CY could promote the rapid initial release of drugs. The release mechanisms significantly improved the bioavailability of drugs, which contributed to strengthening their antibacterial effect, further indicating that the hydrogels are suitable for the treatment of infected wounds. Similarly, the hydrogel (Gel-COS) prepared from chitosan oligosaccharides, phenolic chitosan, and phenolic alginate exhibited excellent self-healing properties and mechanical performance (Figure 5C) 202. The Gel-COS could recover its deformation after four 300% strains, proving that the Gel-COS possesses a favorable self-healing capacity. In addition, there were high-density electrostatic interactions within the Gel-COS polymer chains, which were conducive to maintaining high toughness and flexibility of the hydrogels, further demonstrating that the Gel-COS displays great application potential for the regeneration of irregularly shaped wounds.

Ionic bonds are formed through electrostatic attraction between ionic groups with opposite charges 203. The essence of ionic bonds is electrostatic interactions. The formation of ionic bonds involves adding divalent or trivalent counterions (Ca^2+^, Fe^3+^, and Zn^2+^) to ionic polymer solutions, thereby promoting cross-linking between polymer chains 171. Notably, the ionic cross-linking strength can be regulated according to the type and concentration of metal ions. For example, Wahid *et al*. 204 prepared self-healing hydrogels (CMCH-Zn) by using carboxymethyl chitosan (CMCh) and Zn^2+^. CMCh-Zn possessed good self-healing capacity. More importantly, the mechanical strength of CMCh-Zn was positively correlated with the concentration of Zn^2+^. As a result, hydrogels prepared by regulating the concentration of metal ions can match the mechanical requirements in different application scenarios, which provides novel ideas for the development of intelligent wound dressings. Nonetheless, self-healing hydrogels constructed solely relying on ionic bonds are difficult to simultaneously achieve excellent mechanical properties and long-term structural stability. To address the issue, Yuan *et al*. 205 prepared self-healing hydrogels (PAAc/HACC) via acrylic acid (AAc) and 2-hydroxypropyl trimethylammonium chloride chitosan (HACC) (Figure 5A). AAc polymerized in situ in HACC solution under the partial shielding effect of NaCl salt ions, thereby forming PAAc/HACC hydrogels with high-density dynamic ionic bond characteristics. PAAc/HACC hydrogels had excellent fracture stress (3.31 MPa) and Young modulus (2.53 MPa). Additionally, PAAc/HACC hydrogels could still maintain their original shape after 30 loading and unloading cycles, further indicating that the hydrogels have outstanding fatigue resistance and toughness (Figure 5B). Therefore, this strategy provides a new idea for the design of tough hydrogels. Nevertheless, the spatial distribution of high-density ionic bonds is difficult to precisely control Excessive local charge density may form rigid microregions and inhibit the dynamic reversibility of the hydrogel. Besides, the electrolyte imbalance caused by diabetes may disrupt the accuracy of the salt ion shielding strategy in regulating the density of ionic bonds. Hence, future research should focus on the following aspects: 1) Developing precise control technologies for the spatial distribution of ionic bonds to prevent the formation of rigid microregions caused by excessive local charges; 2) Constructing hybrid networks of ionic bonds with other dynamic bonds (hydrogen bonds and coordination bonds) to achieve the coordinated optimization of the structural stability, self-healing efficiency, and pathological adaptability of hydrogels.

#### 5.1.5. Metal coordination

**Figure 6:**
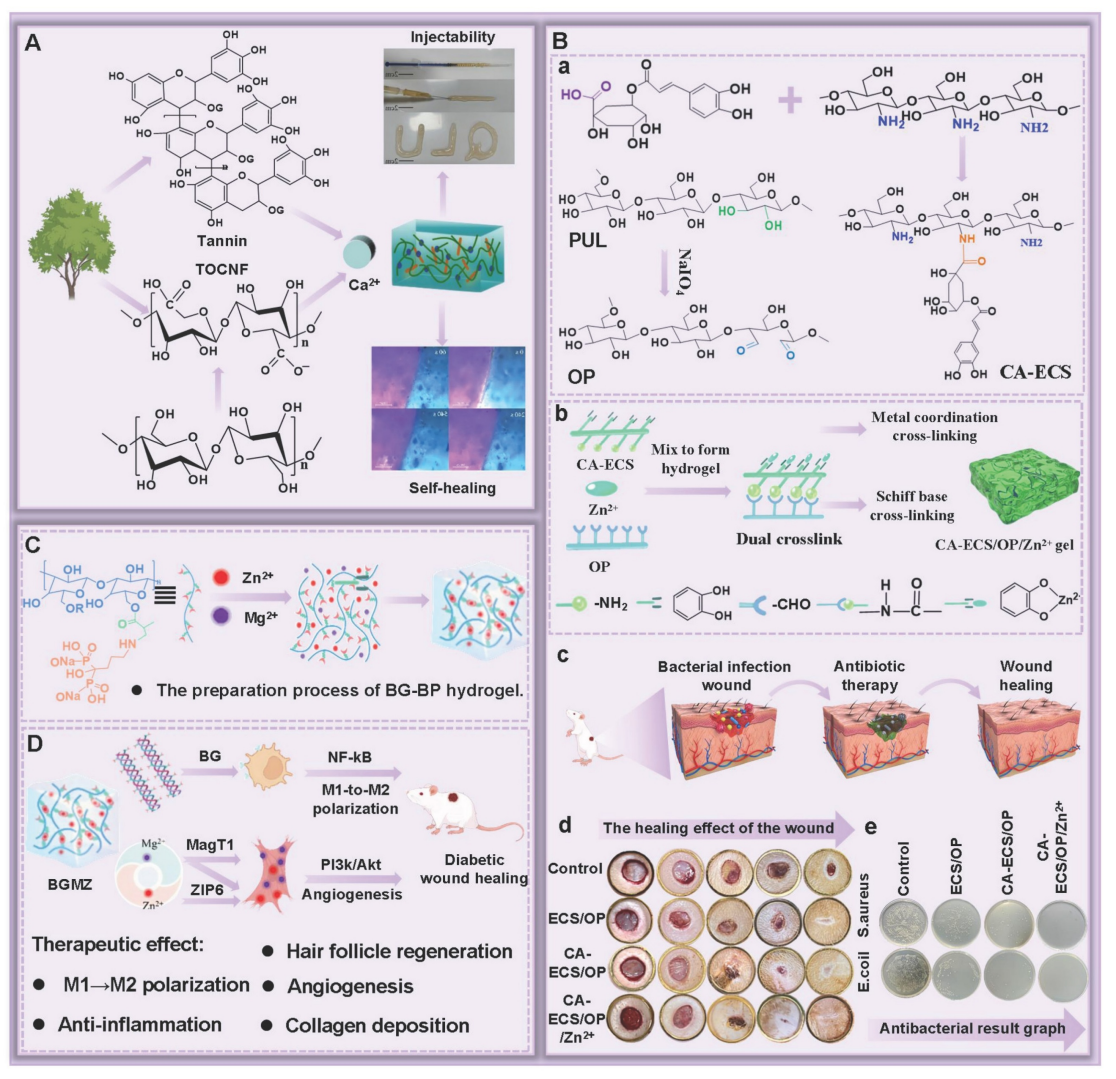
PSHs formed through metal coordination. (A) The preparation process of TOCNF-Ca^2+^-T hydrogel. TOCNF-Ca^2+^-T hydrogel exhibits excellent self-healing ability and injectability. Adapted with permission from 207, copyright 2024 Elsevier. (B) CA-ECS/OP/Zn^2+^ hydrogel design strategy. (a) Synthetic methods of CA-ECS and OP. (b) Synthesis method of CA-ECS/OP/Zn^2+^ hydrogel. (c) Application of CA-ECS/OP/Zn^2+^ hydrogel in wound repair caused by bacterial Infection. (d) The repair process of ECS/OP, CA-ECS/OP, and CA-ECS/OP/Zn^2+^ hydrogel after treating Staphylococcus aureus wound infection at different time points (0 d, 1 d, 5 d, 9 d, 13 d). (e) Antibacterial results of ECS/OP, CA-ECS/OP, and CA-ECS/OP/Zn^2+^ hydrogels. Adapted with permission from 210, copyright 2024 Elsevier. (C) The preparation process of BG-BP hydrogel. (D) The mechanism of BGMZ promoting wound healing in diabetes. Adapted with permission from 214, copyright 2025 Elsevier.

Metal coordination, as a dynamic non-covalent bond, can form reversible bonds with the main chain of polysaccharides, thus effectively promoting the self-healing properties of hydrogels 120. Metal-coordinated hydrogels are physical cross-linked network systems constructed by ligand-functionalized polymers and metal ions (Fe^3+^, Ca^2+^, Zn^2+^, etc.) through dynamic coordination 206. Presently, commonly utilized chelating ligands include low-molecular-weight bisphosphonates, histidine, catechol, thiolates, and carboxylates. These ligands can provide a pair of electrons to metal ions (Fe^3+^, Ca^2+^, Zn^2+^), thereby forming coordination bonds 102. In a research, TEMPO oxidized cellulose nanofibers (TOCNF) and tannin were used as raw materials, and Ca^2+^ was used as the coordination cross-linking agent to design self-healing hydrogels (TOCNF-Ca^2+^-T) (Figure 6A) 207. The TOCNF-Ca^2+^-40%T completely healed within 1 min, thereby demonstrating its rapid self-healing characteristics. Notably, the self-healing ability of metal coordination hydrogels depends on the equilibrium constant (*Keq*) of the coordination bond. Too low a *Keq* value is not conducive to the formation of stable hydrogels, while too high a *Keq* value will show similar covalent bond stability, thus losing the dynamic self-healing ability. Accordingly, an appropriate* Keq* value is the pivotal mechanism for achieving rapid self-healing of hydrogels. However, the stability of metal coordination hydrogels in the diabetic wound microenvironment still faces challenges. For instance, ROS can oxidize ligand groups, such as catechols and thiolates, which may lead to the inactivation of coordination sites. Additionally, the abundant phosphate/carbonate ions (derived from cell breakdown and metabolic products) in the wound can compete with the ligands for metal ions, triggering ion exchange reactions at the coordination sites and causing the reconfiguration of the hydrogel network. Consequently, enhancing the structural stability of metal coordination hydrogels in the pathological microenvironment has become an urgent and important issue to be addressed. Currently, the incorporation of metal coordination into the double network (DN) structure has become a research hotspot in this field 208. For example, Kang *et al*. 209 investigated the self-healing properties of DN hydrogels (CS-PAA-Fe(III)) based on the synergistic effect of Fe^3+^ coordination and hydrogen bonds. The CS-PAA-Fe(III) hydrogels exhibited an excellent self-healing efficiency (93.8%), which was attributed to the rapid reorganization ability of hydrogen bonds and the dynamic reversibility characteristic of metal coordination bonds. Similarly, the synergistic strategy of dynamic covalent bonds (Schiff bases) and metal coordination was also employed to construct high-performance DN hydrogels. The multifunctional DN hydrogels (CA-ECS/OP/Zn²⁺) were prepared via grafting chlorogenic acid onto chitosan (CA-ECS), oxidized branched starch (OP), and Zn^2+^ (Figure 6B) 210. CA-ECS/OP/Zn²⁺ hydrogels had outstanding self-healing ability, injectability, and adhesiveness, which was attributed to the synergistic mechanism between the dynamic reversibility of the Schiff base bond and the structural stability of metal coordination. Besides, compared with other groups, CA-ECS/OP/Zn^2+^ hydrogels had better rigidity and flexibility (33.66 ± 0.59) kPa. Hence, the DN structures with the synergy of metal coordination and dynamic bonds have laid a vital foundation for the construction of high-performance PSHs. For metal-coordinated hydrogels, the introduction of metal ions not only endows hydrogels with self-healing properties, but also with antibacterial, antioxidant, and immunomodulatory activities 211,212. For instance, HA-based self-healing hydrogels (HA-His-Zn) could exert remarkable antibacterial properties (with an antibacterial rate exceeding 99%) through the synergistic coordination of the histidine imidazole groups and Zn^2+^ 213. Additionally, Zn²⁺ could induce macrophages to polarize to the M2 type, thereby regulating the immune response, further demonstrating that HA-His-Zn hydrogels show broad application prospects in the fields of infection and chronic inflammatory wounds. Similarly, the dextran-based hydrogels containing bimetal ions (Zn^2+^ and Mg^2+^) showed outstanding antioxidant activity, which was attributed to the synergistic scavenging effect of the bimetal ions on ROS (Figure 6C) 214. In addition, Mg^2+^ and Zn^2+^ could promote cell proliferation and angiogenesis via up-regulating the PI3K/Akt signaling pathway (Figure 6D). Consequently, this study provides a new design concept for the comprehensive treatment of diabetic wounds. Notably, the long-term release of metal ions may generate cytotoxicity and systemic accumulation 215. In the future, intelligent responsive metal ion controlled-release systems should be developed to achieve on-demand release, thus balancing therapeutic efficacy and biosafety. Moreover, we ought to continuously optimize the synergistic coordination strategy between multi-chelating ligands and metal ions, and improve the various properties of self-healing hydrogels, which are conducive to expanding the application fields of metal coordination hydrogels.

### 5.2. Dynamic chemical covalent bonds

#### 5.2.1. Imine bonds

**Figure 7:**
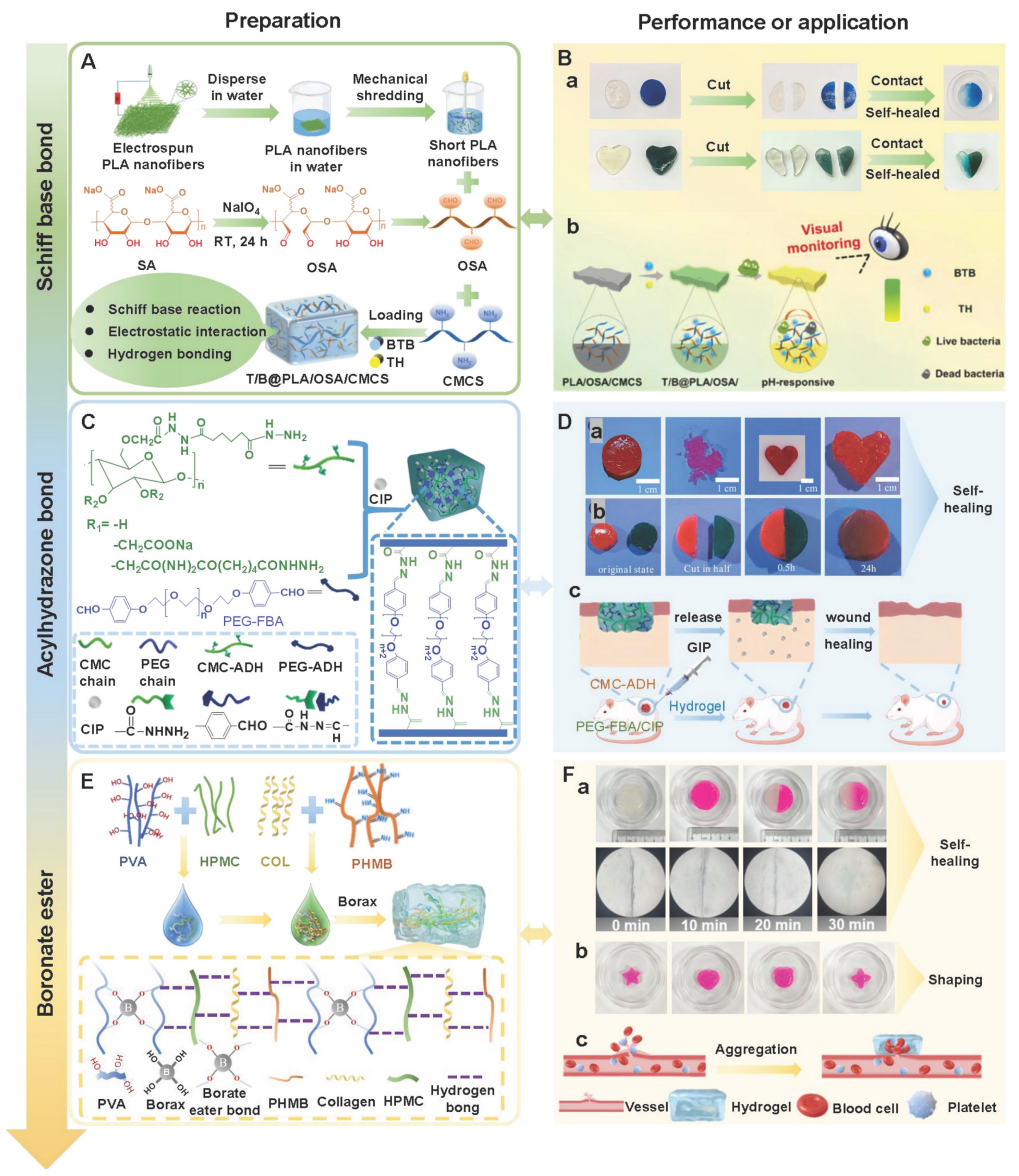
PSHs based on dynamic chemical covalent bonds and their functional properties. (A) Preparation process of T/B@PLA/OSA/CMCS hydrogel. (B): (a) Self-healing process of T/B@PLA/OSA/CMCS hydrogel. (b) Schematic diagram of T/B@PLA/OSA/CMCS hydrogel for in situ visual monitoring and dynamic controllable treatment of bacterial infection wounds. Adapted with permission from 227, copyright 2024 Elsevier. (C) Schematic diagram of CMC-ADH/PEG-FBA hydrogel preparation and gelation mechanism. (D): (a) and (b) demonstrate the self-healing ability of CMC-ADH/PEG-FBA hydrogels. (c) Schematic diagram of CMC-ADH/PEG-FBA hydrogel promoting wound healing. Adapted with permission from 232, copyright 2023 Elsevier. (E) Schematic diagram of the preparation and cross-linking mechanism of CPH hydrogel. (F): (a) The self-healing process of CPH-3 hydrogel at both macroscopic and microscopic levels. (b) Shape adaptability of CPH-3 hydrogel. (c) Hemostasis process of CPH-3 hydrogel. Adapted with permission from 241, copyright 2023 Elsevier.

Imine bonds are dynamic covalent bonds (Schiff bases) formed by the condensation reaction of aldehyde groups and amine groups 216,217. Compared with acyl hydrazone bonds and disulfide bonds, imine bonds have higher bond energy. Additionally, imine bonds possess some advantages, including fast reaction speed and mild reaction conditions, which are more suitable for tissue engineering 218,219. In recent years, polysaccharide skeletons have been rich in functional groups that can participate in Schiff base reactions, thus becoming perfect materials for constructing self-healing hydrogels containing imine bonds 220. For instance, amine-rich polymers, such as CS and polyacrylamide, can be cross-linked with aldehyde-functionalized polymers like oxidized alginic acid and HA, thereby constructing self-healing hydrogels 221. PSHs prepared based on imine bonds have excellent self-healing performance, injectability, and biocompatibility. For example, Li *et al*. 222 prepared self-healing hydrogels (DCMC/CS-DA) through Schiff base reaction using dialdehyde modified carboxymethyl cellulose (DCMC) and dopamine-modified carboxymethyl chitosan (CS-DA) as raw materials. DCMC/CS-DA hydrogels possessed superb self-healing performance. In addition, the equilibrium of imine bonds could be disrupted via amino acid solutions, thus enabling on-demand separation of hydrogels, which was conducive to reducing secondary damage to skin tissues during dressing changes. Accordingly, the outstanding self-healing performance and on-demand separation characteristics of DCMC/CS-DA hydrogels helped them to be candidate materials for ideal wound dressings. Notably, the composite network design provides an effective strategy for optimizing the comprehensive performance of Schiff base hydrogels. In a study, three self-healing hydrogels were designed based on CS, including a single Schiff base network hydrogel (OH), a double Schiff base bond network hydrogel (OHD), and a borate ester bond/Schiff base bond composite network hydrogel (OHPB) 223. The OHPB hydrogel exhibited higher self-healing performance than other groups (the self-healing times of OH, OHD, and OHPB were 3.5 h, 1.5 h, and 0.7 s, respectively). Besides, OHPB hydrogel possessed excellent adhesion and mechanical properties. Hence, developing a composite hydrogel network based on a single Schiff base network is conducive to achieving a win-win situation of the mechanical properties and self-healing performance of hydrogels, which can provide an innovative solution for the preparation of high-performance PSHs. Interestingly, the dynamic covalent properties endow the Schiff base hydrogel with unique pH-responsive behavior 224,225. Diabetic wound infections exhibit a weakly acidic pH. The Schiff base bonds can undergo specific hydrolysis in an acidic microenvironment, which leads to the disintegration of the hydrogel network and the release of drugs, thus enabling autonomous response to pathological signals 226. For example, Jin *et al*. 227 constructed pH-responsive hydrogels using carboxymethyl chitosan (CMCS), sodium oxyalginate (OSA), and tetracycline hydrochloride (TH) as raw materials (Figure 7A). The results exhibit that an acidic environment was more conducive to the release of TH. In addition, TH could reach a dynamic equilibrium 24 h after release, and TH showed long-term and sustained antibacterial activity. Furthermore, BTB was used as a pH indicator to monitor wound infection through visual color changes (Figure 7B). Simultaneously, pH-responsive self-healing hydrogels composed of carboxymethyl chitosan (CMCS) and oxidized hyaluronic acid (OHA) were constructed through Schiff base reaction and loaded with taurine with anti-inflammatory activity 228. The reactive release of taurine molecules accelerated their transfer to the wound site in the acidic environment of diabetic wounds, thereby exerting outstanding anti-inflammatory activity. Notably, the Schiff base bonds can maintain good stability in a neutral pH environment. Therefore, the hydrogel can reversibly release drugs in an acidic infection microenvironment to promote rapid healing, while the hydrogel can maintain structural stability in a neutral physiological condition to support long-term application. In addition to pH responsiveness, the introduction of Schiff base bonds can also significantly enhance the biological functional integration of hydrogels. For instance, the ROS-responsive paeonialoside encapsulated micelles were immobilized to the polysaccharide hydrogel skeletons through Schiff base bonds, thus endowing the hydrogel with anti-inflammatory and pro-angiogenic activities 229. Nevertheless, the stability of Schiff base hydrogel in diabetic wounds still faces several challenges as follows: 1) High glucose concentration may undergo non-specific reactions with amino groups, competitively interfering with the formation and dynamic exchange of Schiff base bonds; 2) The mismatch between the hydrolysis rate and release kinetics can lead to premature drug release/response lag; 3) The reversibility and structural recovery ability of Schiff base bonds under repeated pH fluctuations have not been evaluated. Consequently, future research should focus on the following aspects: 1) Revealing the interference mechanism of high-sugar environment on the formation and exchange kinetics of Schiff base bonds; 2) Regulating the hydrolysis rate of Schiff base bonds to achieve temporal and spatial matching with drug release kinetics; 3) Exploring the reversible behavior and self-healing ability of Schiff base bonds under dynamic pH fluctuations, which provide a theoretical basis for the design of reusable intelligent dressings.

#### 5.2.2. Acylhydrazone bonds

Acylhydrazone bonds are formed through the condensation reaction between aldehydes/ketones and hydrazine. Similar to imine bonds, acylhydrazone bonds can spontaneously form under physiological conditions 230. Notably, acylhydrazone bonds are more stable than amine bonds due to the conjugation effect. The stability of acylhydrazone bonds can be directly translated into advantages in material properties, which is conducive to acylhydrazone bond hydrogels demonstrating superior mechanical strength 231. Li *et al*. 232 prepared self-healing hydrogels (CMC-ADH/PEG-FBA) through grafting carboxymethyl cellulose onto adipic hydrazine (CMC-ADH) and 4-formylbenzoic acid-terminated polyethylene glycol (PEG-FBA) (Figure 7C). The CMC-ADH/PEG-FBA exhibited a favorable self-healing capacity, which was attributed to the reversible break-reassemble mechanism of dynamic acylhydrazone bonds, thereby facilitating the rapid reconstruction of hydrogel network structures (Figure 7D). Importantly, the dominant repairing effect of acylhydrazone bonds can be fully demonstrated in the collaborative network with other dynamic bonds. For instance, Cheng *et al*. 233 designed self-healing hydrogels (PBDS) with a dual cross-linked network via borate ester bonds and amidoxime bonds. Compared with other hydrogels (containing only borate ester bonds), the introduction of dynamic acylhydrazone bonds more significantly improved the self-healing efficiency and flexibility of PBDS hydrogels. Self-healing efficiency has increased from 84.6% to 92.7%, and the elongation has risen from 480% to 1440%. Additionally, the longer the self-healing time, the more obvious the self-healing effect dominated by acylhydrazone bonds. This indicates that acylhydrazone bonds not only exhibit good dynamic reversibility, but also enhance the energy dissipation capacity of the network through cooperative cross-linking design, thus endowing hydrogels with outstanding self-healing efficiency and mechanical stability. More importantly, the multifunctional properties of acylhydrazone bond hydrogels can be further endowed through reasonable molecular structure design. Previous study has introduced the naphthalene-based fluorescent structure into the acylhydrazone-based hydrogel network (CMC-CHO/PEO_23_) 234. CMC-CHO/PEO_23_ hydrogels possessed favorable self-healing performance and mechanical properties. In addition, CMC-CHO/PEO_23_ hydrogels had luminescent properties due to the presence of the naphthalene structure. This was conducive to tracking the degradation behavior of hydrogels, detecting *in vivo* biological toxicity, and evaluating the potential impact of hydrogels on biological cells and tissues, implying that CMC-CHO/PEO_23_ hydrogels show great application prospects in the field of wound dressings.

Acylhydrazone bonds are sensitive to changes in external pH value. Study has shown that acylhydrazone bonds exhibit kinetic inertness under neutral and alkaline conditions, while showing good reversibility in slightly acidic environments (pH 4.0-pH 6.0) 235. Accordingly, the hydrogels prepared by acylhydrazone bonds only have favorable self-healing ability in slightly acidic environments, while reducing their self-healing efficiency in physiologically neutral or alkaline environments 34,236. Notably, imine bonds possess excellent dynamic reversibility in a neutral pH environment, which enables rapid self-healing under physiological conditions. Hence, combining the rapid self-healing ability of imine bonds with the structural stability of acylhydrazone bonds becomes an effective strategy for balancing the self-healing efficiency and mechanical properties of hydrogels. Currently, numerous studies are focusing on constructing synergistic hybrid cross-linking networks based on acylhydrazone bonds and imine bonds. For instance, Zhao *et al*. 237 developed chitosan-based hydrogels based on imine bonds and amidoxime bonds. The hydrogels possessed excellent self-healing efficiency (about 95%) at neutral pH, which was attributed to the fact that imine bonds played a dominant role at neutral pH. Moreover, the self-healing efficiency of the hydrogels could be further enhanced by increasing the temperature and prolonging the healing time. Simultaneously, dextran-based hydrogels based on imine bonds and acylhydrazone bonds had outstanding self-healing properties and mechanical stability (at pH7.4), further suggesting that the construction of an imine bond/acylhydrazone bond synergistic cross-linking network can effectively solve the problem of limited self-healing of acylhydrazone bond hydrogels in a neutral pH environment, thus expanding its application in the field of wound repair 238. Notably, there are still many challenges in the application of acylhydrazone bond hydrogels as follows: 1) The difference in response rates of the two dynamic bonds (acylhydrazone bond and imine bond) in the cooperative network may lead to an uneven cross-linking structure and affect the long-term cycling stability; 2) The study on intelligent-responsive acylhydrazone bond hydrogels (the dual-responsive system of light/enzyme) is not yet complete, which limits the application of acylhydrazone bond hydrogels in complex wounds. In the future, we need to conduct in-depth research on the interaction mechanisms of different dynamic bonds in the collaborative network. Additionally, we also need to explore the intelligent responsive acylhydrazone bond system, thereby expanding the application prospects of acylhydrazone bond hydrogels in the field of wound dressings.

#### 5.2.3. Borate ester bonds

Borate ester bonds, as dynamic covalent bonds, are formed by the condensation reaction of boric acid and diols 239. Compared with other dynamic bonds (hydrogen bonds: 8-29 kJ/mol), borate ester bonds possess higher bond energy (76.7 kJ/mol), which provides a novel strategy for constructing hydrogels with both self-healing performance and high mechanical strength 240. For instance, Ding *et al*. 241 prepared a hydroxypropyl methylcellulose (HPMC) self-healing hydrogel (CPH hydrogel) based on collagen/polyvinyl alcohol (COL/PVA) via polyhexamethylene guanidine (PHMB) as an antibacterial agent and borax as a cross-linking agent (Figure 7E). The CPH hydrogel could achieve complete self-healing within 15 min, further proving that CPH hydrogel shows excellent self-healing capacity (Figure 7F). Besides, the mechanical properties of CPH hydrogel were directly proportional to the number of borate ester bonds. Similarly, the sodium alginate-based hydrogels (PGOP) constructed through the synergistic combination of borate ester bonds and GMA double bonds also possessed outstanding self-healing properties (with a self-healing efficiency of 94.4% ± 2.5%) 242. Additionally, PGOP hydrogels exhibited excellent compressive performance, which was attributed to the dynamic borate ester bond networks that endowed the hydrogels with the ability to deform, implying that borate ester bonds demonstrate great potential in the construction of PSHs. Notably, the stability of borate ester bonds relies on the pH of the environment and the concentration of glucose. Previous study has shown that borate ester bonds could competitively bind with glucose, which led to the dissociation of the hydrogel network structure 203. For instance, Liao *et al*. 243 constructed a CS-based self-healing hydrogel (CBT) by utilizing the glucose-responsive properties of borate ester bonds. The degradation rate of CBT was positively correlated with the glucose concentration in the environment. Moreover, curcumin (Cur) was used as a model drug to research the drug loading and release capacity of CBT. It was found that Cur could be sustainably released from this hydrogel for more than 168 h. In addition, the release rate of Cur was positively correlated with the concentration of glucose in the release buffer. Consequently, the degradation behavior dependent on glucose concentration endows the hydrogel with the ability to release drugs on demand, which is particularly suitable for the treatment of abnormal glucose metabolism diseases (such as diabetic wounds). Except for glucose, the stability of borate ester bonds is highly dependent on the environmental pH 244. It is reported that borate ester bonds could only form when pH was greater than or equal to the pKa value (approximately 8) of phenylboronic acid (PBA), which limited their application in physiological environments (pH 7.4) 245. The imine bonds are dynamically reversible under neutral pH conditions, and the acylhydrazone bonds are reversible under slightly acidic conditions, while the pH-responsive range of the borate ester bonds are significantly biased towards alkaline. These differences provide a basis for the selection of dynamic bonds at different pH stages of diabetic wounds. To overcome the pH application limitations of borate ester bonds, previous study has developed low-pKa value PBA derivatives/highly reactive new diols through molecular structure design, which could facilitate the dynamic cross-linking of borate ester bonds at physiological pH 246. For example, Figueiredo *et al*. 247 prepared self-healing hydrogels using 3,4-dihydro-2H-benzo [e] 1,2-oxaborinin-2-ol (1,2-aborin) and 1-amino-1-deoxyd-fructose functionalized HA as raw materials. The hydrogels possessed excellent self-healing behavior and injectability at physiological pH. Similarly, another research designed a phenylboronic acid/salicylic acid modified HA system had good self-healing ability (repairing within a few seconds) under neutral conditions (pH 7.3) 248. Hence, the molecular structure design can effectively expand the physiological application scope of borate ester bonds. Unfortunately, compared with Schiff base bond and amide bond systems, borate ester bond hydrogels have more complex preparation techniques and extreme reaction conditions, which seriously restrict their clinical transformation 102. Moreover, the balance mechanism between glucose responsiveness and structural stability still lacks systematic research. Hence, we should conduct in-depth research on the quantitative correlation between the density of borate ester bonds and the sensitivity of glucose response, which is necessary to achieve a balance between on-demand drug release and network stability. Additionally, we need to optimize the preparation process of borate ester hydrogels and develop efficient cross-linking strategies under mild conditions, thus facilitating the clinical translation process.

#### 5.2.4. Other dynamic covalent bonds

Disulfide bonds (-S-S-) are composed of cysteine residues or other thiol-containing molecules, and can spontaneously form under physiologically compatible oxidative conditions. Disulfide bonds can be reversibly broken and reorganized through dynamic thiol/disulfide exchange reactions 115. Hence, disulfide bonds possess significant application value in the preparation of PSHs. Nonetheless, disulfide bonds can interact non-selectively with biomolecules (proteins), thereby leading to changes in their natural conformation and impaired biological functions 249. In addition, disulfide bonds are susceptible to REDOX environments and pH changes 250. Human tissues are rich in reducing agent glutathione (GSH), which can affect the stability of disulfide bonds, thereby limiting its application in wound dressings 115. Accordingly, only a few studies have reported the application of disulfide bond hydrogels in wound repair.

Diels-Alder (DA) reaction, as a typical click chemistry reaction, shows significant advantages, including high selectivity and yield, and no by-product generation 184. The reversibility and selectivity of DA reaction are conducive to the construction of PSHs. For example, the cross-linking system of furan-modified hyaluronic acid and maleimide polyethylene glycol is regarded as a typical representative of the DA reaction 251. Nevertheless, the reversible properties of the DA reaction usually require high-temperature conditions (>100 ℃) to be triggered, which limits the ability of the hydrogel to achieve dynamic self-healing at physiological temperatures. Consequently, we can optimize the structure of reactants through molecular design and reduce the reversible temperature threshold of DA reaction, which is conducive to the adaptation of the DA reaction hydrogels to the human physiological environment, thereby expanding their application range. Table 3 summarizes different PSHs and their self-healing characteristics.

## 6. Therapeutic strategies of PSHs in diabetic wounds

**Figure 8:**
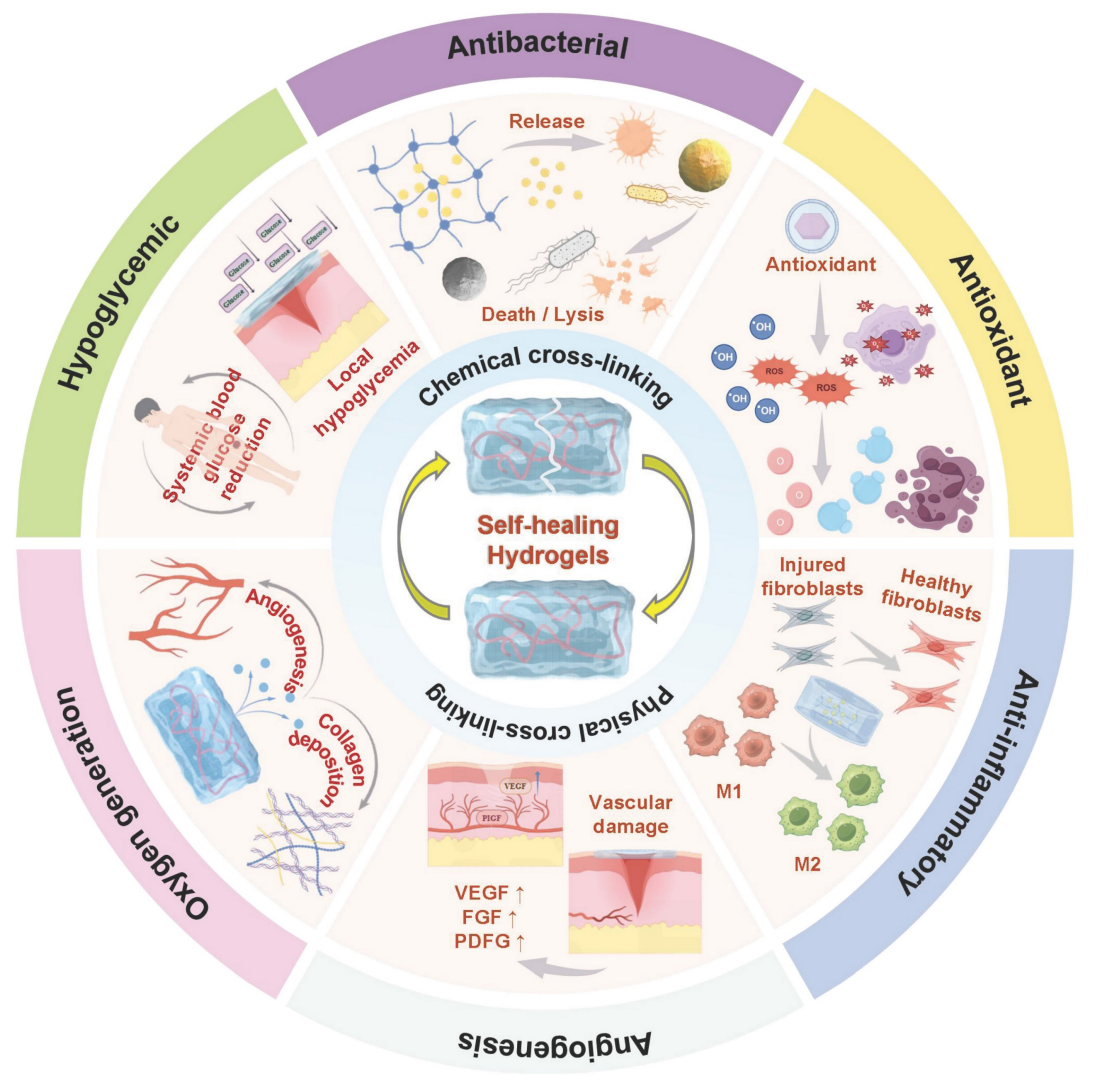
Therapeutic strategies of PSHs in diabetic wounds.

Chronic diabetic wounds have complex pathological microenvironment characteristics, including persistent hyperglycemic state, oxidative stress, chronic inflammatory response, impaired angiogenesis, and susceptibility to bacterial infection, etc. However, traditional hydrogel dressings have a single function and usually only provide basic physical barrier protection, thus failing to meet the needs of the entire diabetic wound healing process. With the advancement of science and technology, new PSHs have been widely used in the treatment of diabetic wounds. New PSHs can integrate multiple biological functions (antibacterial, antioxidant, anti-inflammatory, immunomodulatory, angiogenic promotion, etc.) and also possess intelligent characteristics (stimulus-responsive and controlled-release), which provide an innovative solution for diabetic wound management. Figure 8 represents the therapeutic strategy of PSHs in diabetic wounds. In this section, we focus on discussing the various therapeutic strategies and the latest research progress of PSHs for chronic diabetic wounds. Table 4 shows the therapeutic strategies of PSHs in diabetic wounds.

### 6.1. Antibacterial

Diabetic wounds are highly susceptible to numerous bacterial invasions (*Streptococcus pyogenes*, *Staphylococcus aureus*,* Escherichia coli*, and *Pseudomonas aeruginosa*) due to the harsh environment on the wound surface 252. Bacterial infections are widespread and intractable issues in the process of diabetic wound healing. Bacterial infections can not only cause persistent inflammatory responses, but also lead to wound deterioration, thereby prolonging the healing time 102. Additionally, the high glucose levels in the wound microenvironment provide more nutritional resources for bacterial growth and proliferation, significantly intensifying bacterial infiltration, and ultimately leading to severe infection and even inducing sepsis and amputation 253,254. Traditional hydrogel dressings usually have limitations. For instance, polyvinyl alcohol hydrogels are composed only of hydrophilic polymer networks and bound water. The dressing lacks biological activities and antibacterial function, and the dressing is even prone to bacterial growth 255. Consequently, traditional dressings cannot effectively inhibit or improve bacterial infections in diabetic wounds. Notably, antibiotics are often used in clinical treatment, which accelerates the emergence of drug-resistant strains. Hence, the development of safe, reliable, and antibacterial drug delivery systems holds significant research value. PSHs not only have outstanding air permeability, moisture absorption, and exudate absorption capacity, but also can effectively avoid secondary damage caused by frequent dressing changes. More importantly, PSHs can efficiently inhibit bacterial proliferation, significantly reduce the incidence of infection, and accelerate the wound repair process 254,256. Relevant studies have shown that PSHs were typically classified into hydrogels with intrinsic antibacterial activity and hydrogels loaded with antibacterial substances (antibiotics, antimicrobial peptides, metal nanoparticles, and photodynamic drugs) 255,257.

**Figure 9:**
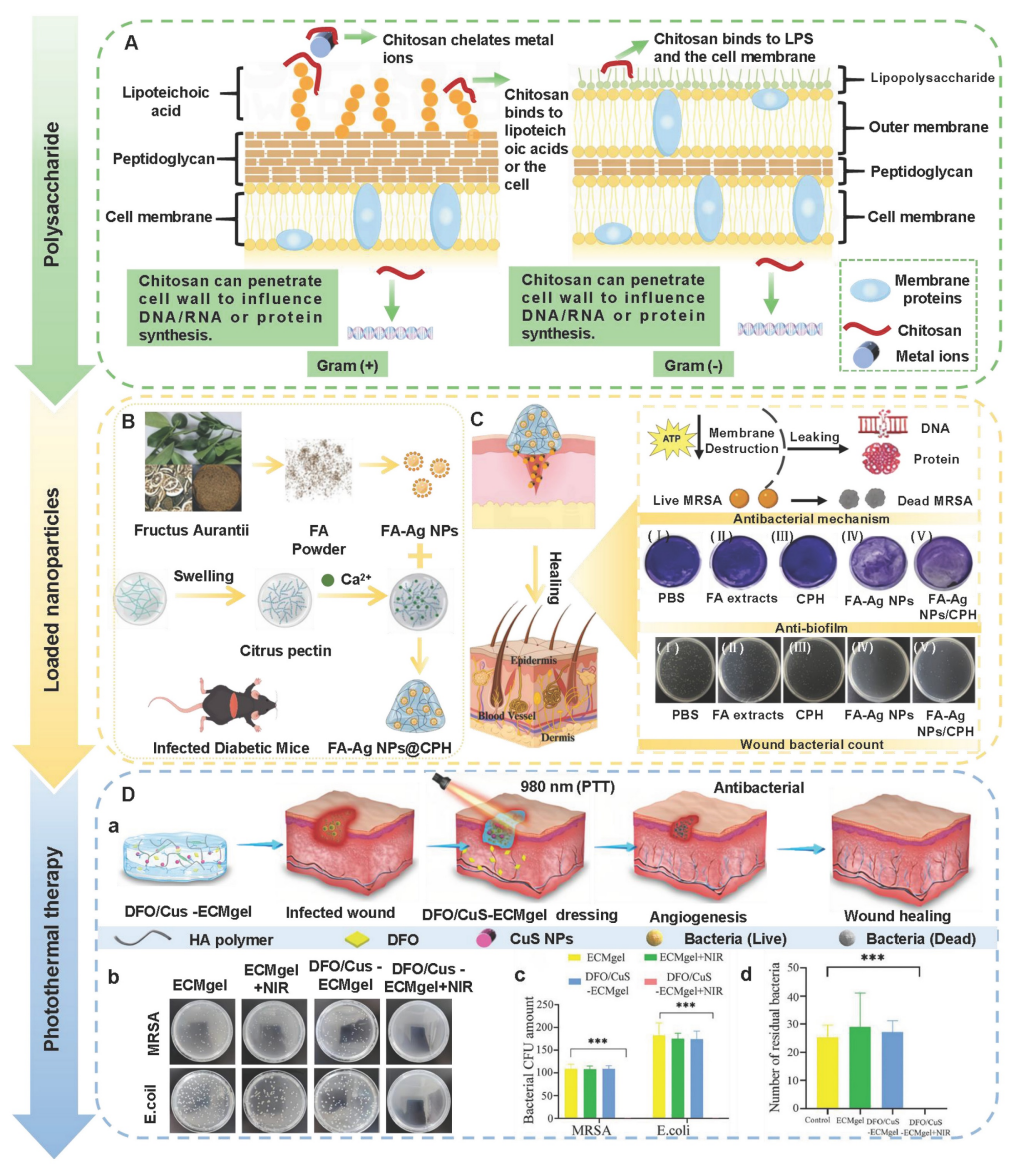
The antibacterial mechanisms and effects of PSHs. (A) Schematic diagram of the antibacterial mechanism of CS. Adapted with permission from 28, copyright 2025 Elsevier. (B) Preparation process of FA-Ag NPs/CPH. (C) Diagram of antibacterial activity of FA-Ag NPs/CPH. (Ⅰ) to (Ⅴ) are bacterial counts in biofilms and wounds treated with PBS, FA extracts, CPH, FA-Ag NPs, and FA-Ag NPs/CPH. Adapted with permission from 261, copyright 2025 Elsevier. (D): (a) DFO/Cus-ECMgel hydrogel combined with photothermal therapy promotes the wound healing process of diabetes. (b) Schematic diagrams of *E. coli* and *MRSA* colonies formed after various treatments. (c) The corresponding CFU counts of *E. coli* and *MRSA* treated in various ways. (d) Quantitative analysis of residual bacteria at wound sites. Adapted with permission from 262, copyright 2024 Wiley.

Polysaccharide organisms are typical antibacterial cationic polymers. For instance, the antibacterial properties of CS stem from its positively charged amino groups. CS can disrupt the cellular structure of microorganisms through charge interactions, increasing the permeability of biofilms, and inhibiting the formation of biofilms in acidic environments (pH < 6.5). Additionally, CS can bind to microbial DNA, thereby inhibiting the synthesis of mRNA and protein (Figure 9A) 28,102. CS has natural antibacterial properties. Hence, there is no need to introduce additional antibacterial agents during the preparation of hydrogels. Regrettably, these hydrogels still have some limitations, including a narrow antibacterial spectrum, the influence of pH value and microenvironment at the injury site, and poor mechanical strength. To address the above limitations, we can optimize the performance of hydrogels by introducing various antibacterial materials (metal ions, antibacterial peptides, and natural antibacterial agents) 258. In recent years, metal ions have emerged as one of the most widely used antibacterial agents due to their strong antibacterial activity 98,257. Currently, nanomaterials have become a research hotspot, which is attributed to their unique and controllable antibacterial properties 259,260. Among them, silver nanoparticles (AgNPs) possess broad-spectrum antibacterial activity and cytocompatibility, and AgNPs are often introduced into polysaccharide hydrogels to exert a synergistic antibacterial effect. Li *et al*. 261 introduced Fructus Aurantii (FA)-derived green synthetic nanoparticles (FA-AgNPs) into citrus pectin hydrogel (CPH) and evaluated their antibacterial properties (Figure 9B). The hydrogel showed good antibacterial activity, and its antibacterial rate against *MRSA* was 97.66% ± 1.10%. In addition, the hydrogel demonstrated effective anti-biofilm activity, which provided a strategy for the management of *MRSA* infection in diabetic wounds (Figure 9C). More importantly, the utilization of FA to reduce Ag^+^ to Ag effectively avoided the potential toxicity of AgNPs, which could promote the clinical transformation of polysaccharide hydrogels loaded with AgNPs. Nanomaterials possess the ability of photothermal conversion. PSHs prepared by combining nanoparticles with photothermal therapy exhibit superior antibacterial activity. Previous study has shown that the synergistic effect of HA hydrogel loaded with copper sulfide nanoparticles and photothermal therapy was conducive to enhancing the antibacterial effect of the hydrogel (with antibacterial rates of over 99% against *E. coli* and *MRSA*), further suggesting that the combination of nanomaterials and photothermal therapy provides an effective solution for diabetic infection wounds (Figure 9D) 262. Notably, there are still many bottlenecks in the application of nanomaterials as follows 263: 1) The nanoscale of nanomaterials endows them with high reactivity and transmembrane ability, which can induce intracellular oxidative stress, inflammatory responses, and DNA damage, thereby increasing cytotoxicity; 2) The long-term accumulation of some non-degradable nanomaterials in the body may trigger chronic inflammation or tissue fibrosis; 3) The metabolic pathways of nanomaterials in the body (renal excretion and liver-biliary system clearance) are unclear, which increases the difficulty of biological safety assessment; 4) The complex synthesis process of nanomaterials leads to a significant increase in the cost of hydrogel preparation, which limits large-scale clinical application. Consequently, future research should focus on the following aspects: 1) Developing biodegradable green nanomaterials with low long-term accumulation risks; 2) Establishing a standardized nanotoxicology evaluation system, combining *in vitro* models and long-term animal experiments, and assessing the safety of nanomaterials in the wound microenvironment; 3) Optimizing the synthesis process of nanomaterials, exploring green and low-cost large-scale preparation methods to promote clinical translation; 4) Constructing intelligent responsive nanocomposite hydrogels to achieve a dynamic balance between antibacterial activity and wound healing promotion functions.

### 6.2. Antioxidant

Pathological levels of ROS (H_2_O_2_, ·OH, and O^2-^) can interfere with the wound healing process in diabetes through various pathways as follows 262,264: 1) ROS can directly oxidize and damage lipids, proteins, and DNA, which lead to dysfunction of endothelial cells and fibroblasts; 2) ROS promotes the release of inflammatory factors and exacerbates inflammatory responses via activating the NF-κB signaling pathway. Notably, the inflammatory response can recruit more inflammatory cells and generate additional ROS, which further amplify oxidative damage. Hence, antioxidation becomes a crucial approach for promoting wound repair in diabetes. Among various therapeutic strategies, PSHs stand out for their excellent biocompatibility and rich biological functions. PSHs with antioxidant properties can eliminate excessive ROS, alleviate oxidative stress responses, improve the microenvironment of wounds, and ultimately achieve rapid wound repair 265. Numerous studies have confirmed that the antioxidant mechanisms of PSHs mainly consisted of two aspects as follows 253,255: 1) Directly eliminating ROS by utilizing the inherent antioxidant groups of polysaccharide molecules; 2) Loading ROS scavengers or constructing ROS-responsive structures in the hydrogel network, thus endowing hydrogels with antioxidant properties.

**Figure 10:**
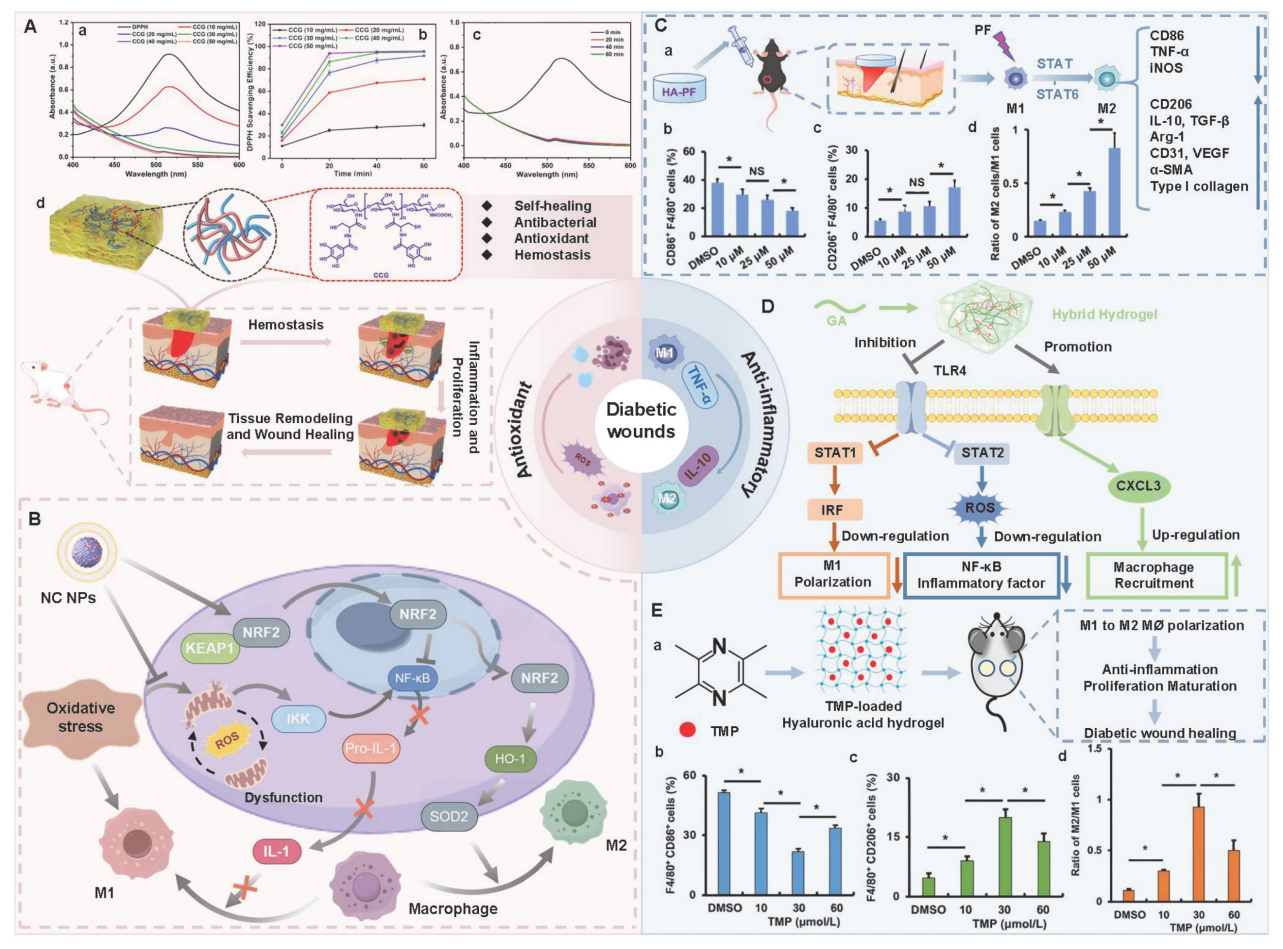
The antioxidant and anti-inflammatory mechanisms and effects of PSHs. (A): (a) Ultraviolet-visible spectra of the reaction between the CCG of different concentrations and DPPH. (b) The DPPH removal efficiency of CCG with different concentrations. (c) Ultraviolet-visible spectra of the reaction between CCG (50 mg/mL) and DPPH at different time intervals. (d) Schematic diagram of CCG promoting skin wound healing. Adapted with permission from 271, copyright 2024 Elsevier. (B): The antioxidant and anti-inflammatory mechanisms of NC NPs. Adapted with permission from 272, copyright 2025 Elsevier. (C): (a) HA-based hydrogels loaded with PF regulate the phenotypic transformation of macrophages. (b) CD86 and F4/80 cell populations (%) of macrophages treated with dimethyl sulfoxide (DMSO) and PF (10 μM, 25 μM, and 50 μM). (c) CD206 and F4/80 cell populations (%) of macrophages treated with dimethyl sulfoxide (DMSO) and PF (10 μM, 25 μM, and 50 μM). (d) Ratio of M2 cells (CD206 and F4/80) to M1 cells (CD86 and F4/80) after macrophages were treated with dimethyl sulfoxide (DMSO) and PF (50 μM). Adapted with permission from 282, copyright 2021 Elsevier. (D) Hybrid hydrogels containing GA inhibit the expression of M1 macrophages. Adapted with permission from 279, copyright 2022 Wiley. (E): (a) HA hydrogels loaded with TMP regulate macrophage polarization. (b) The number of M1 cells (%) after treatment with DMSO and TMP. (c) The number of M2 cells (%) after treatment with DMSO and TMP. (d) M1/M2 macrophage ratio. Adapted with permission from 284, copyright 2023 Elsevier.

First of all, natural polysaccharides have a certain ability to scavenge free radicals. In addition, natural polysaccharides can up-regulate the activity of antioxidant enzymes or synergistically down-regulate the oxidative stress response mediated by oxidase, thereby achieving multi-pathway antioxidant regulation 258. PSHs with inherent ROS scavenging activity can simplify the preparation process and avoid the adverse effects of drug loading 266. For instance, the uronic acid groups in pectin or HA are important molecules with antioxidant activity. A relevant report has shown that compared with polysaccharides without uronic acid, polysaccharides with a high content of uronic acid exhibited stronger free radical scavenging capacity 267. Apart from pectin and HA, CS and its derivatives also possess good antioxidant activity, which is attributed to the fact that their molecular structures are rich in active -OH and -NH_2_, thus acting as electron donors to neutralize O^2-^. Numerous studies have illustrated that the free radical scavenging ability of CS was related to the degree of deacetylation 268,269. CS with a high deacetylation rate (> 98%) exhibited a superior ROS clearance effect. Moreover, the radical scavenging rate of CS was also related to its molecular weight. Low-molecular-weight CS could inhibit the formation of intramolecular/intermolecular hydrogen bonds and generate more -OH and -NH_2_, which was conducive to eliminating free radicals. Nonetheless, relying solely on the inherent antioxidant capacity of polysaccharides is insufficient to cope with the intense oxidative stress response in diabetic wounds. Fortunately, the addition of ROS scavengers significantly enhances the antioxidant capacity of PSHs. In recent years, polyphenolic compounds (gallic acid, quercetin, tannic acid, catechin, curcumin) have attracted much attention due to their outstanding antioxidant properties 10,270. The gallic acid molecule contains three phenolic hydroxyl groups, which can consume free radicals by providing hydrogen atoms or electrons, thereby becoming an ideal natural antioxidant. Yang *et al*. 271 investigated the antioxidant activity of CS-based hydrogels loaded with gallic acid (CCG). The DPPH removal efficiency of CCG hydrogel was directly proportional to its concentration. The removal efficiency of DPPH by CCG hydrogel (50 mg/mL) reached 95.83%, further suggesting that CCG hydrogel possesses strong antioxidant performance (Figure 10A). Previous study has shown that the self-assembled herbal nanoparticles (NC NPs) composed of curcumin and naringenin had excellent antioxidant properties (Figure 10B) 272. Compared with the control group, the hydrogel group loaded with NC NPs demonstrated higher ROS clearance activity (92.67%). Moreover, NC NPs could improve mitochondrial function through multiple pathways as follows: 1) Removing mitochondrial ROS to alleviate damage caused by oxidative stress; 2) Regulating mitochondrial calcium ion levels to maintain ionic homeostasis; 3) Enhancing the mitochondrial antioxidant defense system and increasing the expression of antioxidant enzymes (SOD and CAT). Restoring mitochondrial homeostasis was crucial for reversing oxidative stress damage. Apart from gallic acid and curcumin, quercetin and tannic acid have also become current research hotspots due to their excellent antioxidant properties. For instance, Gong *et al*. 273 prepared a carboxymethyl chitosan-based hydrogel loaded with quercetin and tannic acid. The hydrogel group exhibited the highest free radical scavenging activity, which was mainly attributed to the synergistic antioxidant effect of quercetin and tannic acid. Additionally, the hydrogel could effectively shorten the wound healing time of type II diabetic rats and reduce scar formation, further suggesting that the hydrogel has potential application value in the management of diabetic wounds. Hence, the construction of PSHs with antioxidant activity has become a crucial strategy for alleviating oxidative stress and promoting wound healing. Nevertheless, there are certain limitations in the practical application of antioxidants as follows: 1) A single antioxidant can only remove a limited number of ROS types; 2) High-dose application may trigger pro-oxidative effects and cause cell damage; 3) The antioxidant activity is easily affected by environmental factors (pH and temperature), which lead to a decrease in stability; 4) The release behavior of loaded antioxidants is difficult to be precisely controlled. In the future, we can utilize nanoscale encapsulation technology to enhance the stability and bioavailability of antioxidants. Additionally, we should continuously explore the synergistic mechanisms of various antioxidants to achieve broad-spectrum ROS clearance.

### 6.3. Anti-inflammatory and immune regulation

The disorder of inflammation resolution is the core feature of diabetic wounds. The essence of inflammatory resolution disorder lies in the obstruction of the transformation of macrophages from pro-inflammatory M1-type to pro-regenerative M2-type 274,275. The accumulation of M1-type macrophages can stimulate the excessive secretion of pro-inflammatory factors (TNF-α, IL-6, and IL-1β) 276,277. Additionally, the levels of pro-healing factors (IL-10, TGF-β, and VEGF-A) secreted by M2-type macrophages significantly decrease 278. The aforementioned imbalance in transformation can slow down the tissue regeneration process and exacerbate the difficulty in healing diabetic wounds. Currently, antibiotics, growth factors, and anti-inflammatory drugs are mainly used in clinical practice to alleviate chronic inflammation. Unfortunately, the above-mentioned drugs are confronted with challenges, such as easy inactivation, drug resistance, and other side effects 252. Regulating the inflammatory microenvironment via adjusting the phenotype and quantity of immune cells is a reliable alternative approach. It has been reported that disrupting the ROS inflammatory cycle and promoting the transformation of macrophages from M1 type to M2 type could significantly enhance the therapeutic effect on dorsal skin and foot wounds in diabetic rats 277. Consequently, the application of hydrogels to regulate the macrophage phenotype has become a promising therapeutic strategy for alleviating excessive inflammatory stimulation and immune dysfunction in diabetic wounds 279. Numerous studies have confirmed that the immunomodulatory and anti-inflammatory mechanisms of PSHs are as follows 252,278: 1) Some PSHs have inherent immunomodulatory and anti-inflammatory activities due to their specific physical structure or chemical composition; 2) PSHs can enhance their anti-inflammatory and immunomodulatory activities via loading anti-inflammatory molecules (curcumin, paeoniflorin, and glycyrrhizic acid), cytokines and antibodies (anti-TNF-α antibody and VEGF), antimicrobial peptides, nucleic acids (DNA and RNA), and other substances.

PSHs (HA and CS) have been proven to possess inherent immunomodulatory and anti-inflammatory activities. For instance, high molecular weight HA can activate downstream anti-inflammatory signaling pathways by binding to CD44 receptors and promoting the transformation of macrophages from M1 type to M2 type 280. Besides, HA can interact with hyaluronic acid adhesive to further exert anti-inflammatory effects. Hydrogels rich in HA can bind to TSG-6, inhibit the migration of neutrophils and plasmin activity through negative feedback mechanisms, and reduce the release of inflammatory mediators, thereby demonstrating favorable anti-inflammatory properties 172,281. On this basis, introducing substances with immune-regulating functions and anti-inflammatory activities into hydrogels is an effective method to improve their immune-regulating and anti-inflammatory effects. In recent years, many active compounds derived from traditional herbs, such as paeoniflorin (PF) and glycyrrhizic acid (GA), have been proven to regulate macrophage polarization, thus displaying potential therapeutic effects on diabetic wounds 281. The number of cells expressing CD86 and F4/80 (two commonly used phenotypic markers of M1 macrophages) decreased significantly (*p* < 0.05) after PF treatment of macrophages, while increasing the number of cells expressing CD206 and F4/80 (two commonly used phenotypic markers of M2 macrophages) (Figure 10C). In addition, PF can regulate the function of macrophages, inhibit the release of pro-inflammatory factors, and increase the secretion of anti-inflammatory factors 282. GA is a natural compound with intrinsic immune regulatory activity. Similar to PF, GA can promote the transformation of macrophages from M1 type to M2 type (Figure 10D). Moreover, GA can significantly reduce neutrophil infiltration and the expression of pro-inflammatory factors and promote the transition of diabetic wounds from the inflammatory phase to the proliferative phase 279. Tetramethylpyrazine (TMP) isolated from traditional herbs possesses anti-inflammatory, anti-apoptotic, and antioxidant properties, and TMP can be used as a potential drug for the treatment of type Ⅱ diabetes 283. Hence, we can introduce them into PSHs to prepare hydrogel dressings with immunomodulatory and anti-inflammatory functions, which has become an effective therapeutic strategy for promoting wound healing in diabetes. Chu *et al*. 284 prepared HA hydrogel loaded with TMP (TMP-HA). TMP-HA showed better anti-inflammatory activity than the control group, and TMP-HA could significantly promote the polarization of macrophages from M1 type to M2 type (Figure 10E). Additionally, TMP-HA effectively regulated the balance between STAT1/iNOS and STAT6/Arg-1, and TMP-HA could obviously reduce the recruitment of inflammatory cells in the wounds of diabetic mice. Furthermore, TMP-HA could also promote epidermal hyperplasia, angiogenesis, and collagen deposition at the wound site, further suggesting that TMP can alleviate the inflammatory damage of diabetic wounds, thereby demonstrating the application prospects of PSHs loaded with traditional herbal medicines in the treatment of diabetic wounds.

All in all, PSHs possess excellent immunomodulatory properties and drug-loading capabilities, which provide an effective solution for breaking the vicious cycle of inflammation. Currently, PSHs that integrate traditional herbal active ingredients (PF, GA, TMP) have attracted much attention. These hydrogels can significantly promote the polarization of macrophages (from M1 type to M2 type), achieving a harmonious balance between the inhibition of inflammation in diabetic wounds and tissue regeneration. In the future, we can focus on nanocarriers to enhance the continuous release of anti-inflammatory molecules at the wound site, thereby improving the targeting of drugs. In addition, exploring more stable and safe anti-inflammatory agents remains a vital research direction in the field of diabetic wound treatment.

### 6.4. Angiogenesis

The core issue of wound healing in diabetes remains insufficient blood supply, which is attributed to impaired angiogenesis, especially microangiogenesis. Microangiogenesis is crucial for transporting oxygen, nutrients, and growth factors in the early stage of wound healing 285. The proliferation, migration, and differentiation of cells and the formation of extracellular matrix largely depend on the oxygen and nutrients carried and provided by the blood. Insufficient angiogenesis can lead to the accumulation of a large amount of glucose at the wound site, thereby causing ischemia and tissue necrosis 286,287. Nevertheless, the high glucose microenvironment can further induce the defect of HIF-1α transcriptional activation. HIF-1α is not only a pivotal regulatory factor for angiogenesis, but also a transcription factor that regulates the expression of VEGF. Defects in the expression of HIF-1α will further impede neovascularization, thereby forming vicious cycles 288. Accordingly, we should break through the passive protection mode of traditional dressings and effectively promote angiogenesis in the early stage of diabetic wounds, thereby preventing deterioration of the wounds and promoting wound healing. At present, the strategies for promoting angiogenesis by using PSHs are as follows: 1) Cell therapy; 2) Vascular growth factors and angiogenesis-related active substances.

**Figure 11:**
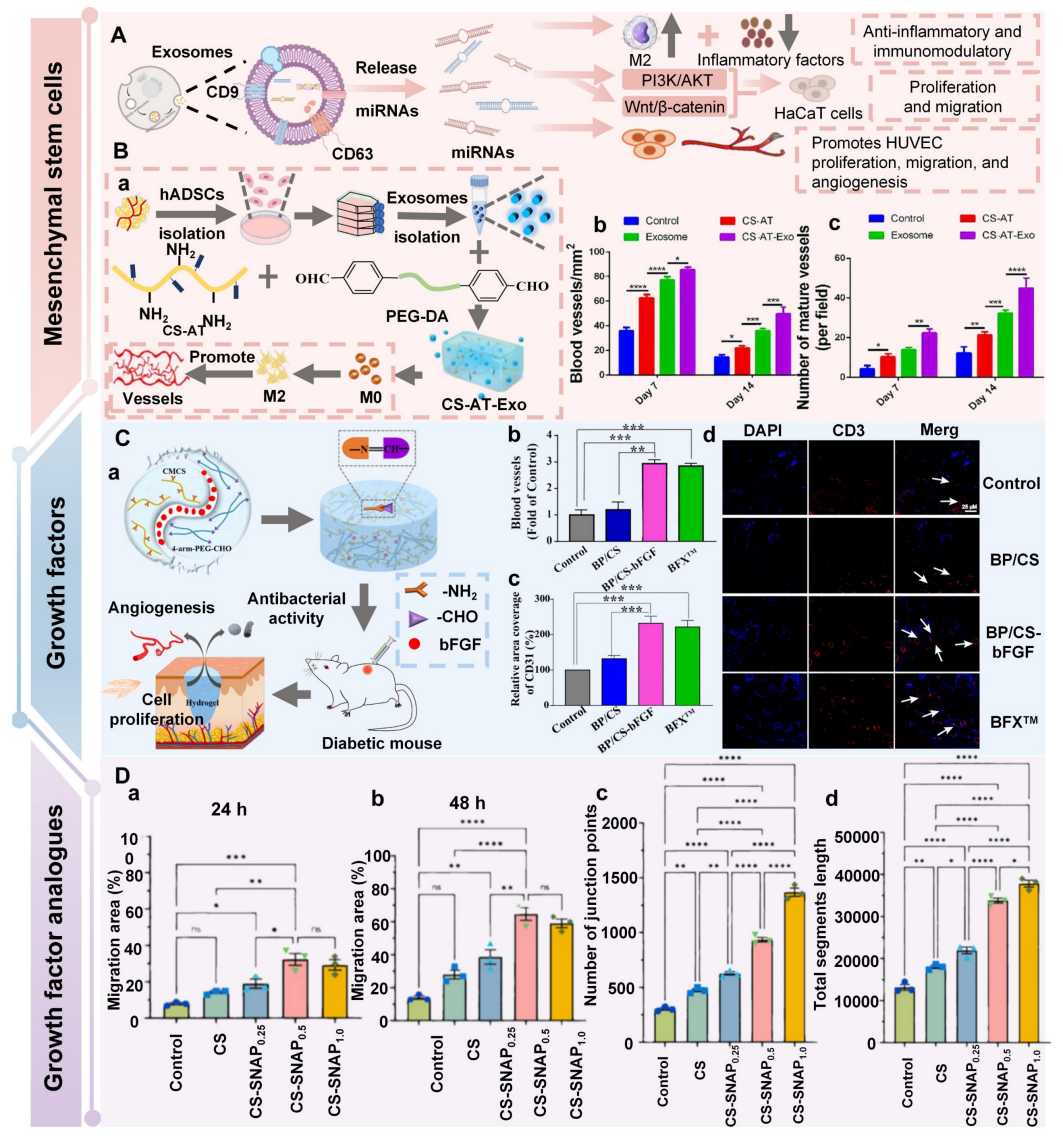
Antiangiogenic activity of PSHs. (A) Diagram of the mechanism of exosomes promoting angiogenesis. Adapted with permission from 290, copyright 2023 Wiley. (B): (a) Diagram of the preparation process of CA-AT-Exo hydrogel loaded with exosomes and its mechanism of promoting angiogenesis. (b) The total number of blood vessels at the wound site on the 7th and 14th days. (c) The number of mature blood vessels in the wound on the 7th and 14th days. Adapted with permission from 294, copyright 2023 Elsevier. (C): (a) The preparation process of BP/CS-bFGF hydrogel and its application in wound healing of diabetic mice. (b) The number of new blood vessels in different groups. (c) Histochemical staining results of CD31 on the 14th day after treatment in different groups. (d) Immunofluorescence staining results of CD34 on the 14th day after treatment in different groups (the white arrow points to the blood vessels). Adapted with permission from 100, copyright 2022 Elsevier. (D): (a) and (b) represent the relative migration rates of human skin fibroblasts at 24 h and 48 h. (c) and (d) represent the quantification of angiogenesis in different groups, including the number of connection points and the total length. Adapted with permission from 297, copyright 2026 Elsevier.

Mesenchymal stem cells (MSCs) have been proven to play a crucial role in tissue regeneration and wound repair 289. A relevant study has exhibited that MSCs could promote angiogenesis in damaged tissues, regulate ECM remodeling, and accelerate the re-epithelialization process of wounds (Figure 11A) 290. In addition, MSCs could secrete immunomodulatory factors, remodeling molecules, and extracellular vesicles (EVs), thus promoting the formation of granulation tissue in wounds 291. The latest research highlights that exosomes (Exos) secreted by MSCs, as the main bioactive components, can deliver bioactive molecules (functional miRNAs, proteins, and lipids), thereby playing a key role in tissue repair 292. For instance, Exos are rich in miRNA, which can promote the proliferation and migration of epidermal cells through the PI3K/Akt pathway and the Wnt/β-catenin pathway. Moreover, miR-128-3p, miR-125a-3p, and miR-126-3p carried by Exos can increase the viability of vascular endothelial cells and promote the migration of vascular endothelial cells and lumen formation 290. Nonetheless, direct application of Exos to diabetic wounds can lead to their rapid inactivation and degradation. PSHs loaded with Exos exhibit favorable stability in a high-glucose microenvironment and achieve continuous and controllable release, which can significantly enhance the therapeutic effect of Exos 293. Wang *et al*. 294 prepared multifunctional CS-based self-healing hydrogels (CS-AT-EXO) using chitosan-graft-aniline tetramer (CS-AT) and dibenzaldehyde-terminated polyethylene glycol (PEG-DA) as raw materials. The hydrogels could exert sustained-release effects by loading Exo and promote the wound healing process in diabetes. Compared with the treatment with Exo and CS-AT hydrogels alone, the expression levels of CD31 were the highest in the CS-AT-EXO group. Additionally, the CS-AT-Exo group showed a greater number of mature blood vessels than other groups, and the vascular bed was denser, and the lumen was more obvious (Figure 11B). Furthermore, the co-culture of CS-AT-Exo hydrogels with macrophages could significantly enhance the proliferation, migration, and lumen formation ability of human umbilical vein endothelial cells (HUVEC). Therefore, PSHs with continuous Exos release function have extremely high research value in promoting angiogenesis in diabetic wounds.

Vascular growth factors (VEGF, bFGF, EGF, etc.) can directly stimulate endothelial migration and angiogenesis. Unfortunately, vascular growth factors have obvious limitations, such as high production costs and poor *in vivo* stability (short half-life), which restrict their clinical application 295. To address this issue, we can adopt PSHs as carriers for sustained-release regulation or use alternative solutions (such as growth factor mimics). For example, Hao *et al*. 100 prepared CMCS-based composite hydrogels (BP/CS-bFGF) using benzaldehyde-terminated four-arm polyethylene glycol (4-arm-PEG-CHO) and carboxymethyl chitosan (CMCS) as raw materials, and loaded bFGF. The hydrogels loaded with bFGF could up-regulate the expression levels of CD31 and CD34, and enhance the formation of new blood vessels (Figure 11C). In addition, BP/CS-bFGF hydrogels could up-regulate the production of Ki-67 and significantly promote the generation of collagen fibers and epithelial metaplasia, thereby accelerating full-thickness wound repair in diabetic mice. Similarly, Yang *et al*. 296 investigated the angiogenic activity of hydrogels loaded with panaxoside (PNS) and insulin-like growth factor 1 (IGF-1). Compared with the control group, the hydrogel group loaded with PNS and IGF-1 exhibited stronger angiogenic ability. Moreover, the hydrogels could increase the survival rate of endothelial cells, migration ability, and the formation of tubular structures, further suggesting that the hydrogels have the ability to restore vascular function. This was mainly attributed to the fact that PNS and IGF-1 could regulate vascular repair through signaling pathways, such as NF-κB, PI3K-Akt, and TNF. Consequently, hydrogels loaded with vascular growth factors provide a promising treatment option for diabetic wound management. Nevertheless, there are many challenges in the application of growth factor delivery strategies for promoting angiogenesis in diabetic wounds as follows: 1) The high expression of proteases (MMPs) in the wound can rapidly degrade growth factors, which leads to the loss of biological activity; 2) The burst release effect of growth factors cannot match the long-term requirements of vascular regeneration; 3) A single growth factor is difficult to simulate the synergistic effects of multiple factors in the physiological process of angiogenesis; 4) High-dose growth factors may induce side effects, such as hemangiomas, which increases safety risks; 5) The maintenance of growth factor activity requires strict conditions for production, storage, and transportation, which increases the production cost and quality control difficulty.

In addition to vascular growth factors, angiogenesis-related active substances (such as plant extracts, metal ions, and NO donors) have demonstrated significant value in the treatment of diabetic wounds due to their advantages of high stability, low cost, and low immunogenicity. For instance, previous study has designed environment-activated hydrogels based on CS 297. The hydrogels could achieve spatiotemporal controlled release of NO using environmental light and temperature (over 300 min). NO could promote vasodilation and angiogenesis, control cell proliferation and migration, and regulate cytokine secretion (Figure 11D). Compared to the control group, the hydrogels group showed stronger angiogenic activity and a higher endothelial cell proliferation rate. Additionally, CS and NO exerted synergistic antibacterial activity, which was beneficial for controlling wound infections at the early stage. Therefore, angiogenesis-related active substances have become promising candidate materials for diabetes wound management and skin regeneration. In the future, we can focus on the following strategies: 1) Develop new plant polyphenols and functional short peptides with vasodilatory effects, reducing immunogenicity and cost; 2) Construct an oxygen-deficient simulated microenvironment to activate endogenous VEGF signaling; 3) Deeply explore the structure-activity relationship of polysaccharides in promoting angiogenesis; 4) Develop intelligent responsive hydrogels to achieve on-demand release of angiogenic signals.

### 6.5. Hypoglycemic

Hyperglycemia can lead to abnormal accumulation of AGEs, which is the core mechanism that triggers tissue damage. AGEs, as signaling molecules, directly activate immune cells and initiate downstream pro-inflammatory signaling pathways via binding to the RAGE receptor, thereby inducing oxidative stress responses 298. In addition, AGEs can capture and cross-link ECM proteins, which leads to thickening of the vascular basement membrane and microcirculation disorders. This directly hinders tissue perfusion and creates a hypoxic microenvironment conducive to the accumulation of AGEs 299. Therefore, the key therapeutic measure is to effectively control glucose levels in the treatment of diabetic wounds. Traditional treatment methods (oral hypoglycemic drugs, blood glucose monitoring, etc.) can generate problems, such as insulin resistance and increased risk of infection, which seriously affect the therapeutic effect of diabetic wounds 259. Besides, most wound dressings neglect the effective regulation of local glucose levels in the lesion 300. Accordingly, the preparation of hydrogel dressings that can regulate the high-glucose microenvironment has broad application prospects in the field of treating diabetic wounds 301. Currently, there are roughly two types of treatment strategies for the hyperglycemic microenvironment of diabetic wounds as follows: 1) Loading hypoglycemic active substances (metformin, insulin, glucose oxidase (GOx), etc.) for local blood glucose regulation, thereby promoting wound healing; 2) Adopting local-systemic coordinated treatment strategies to achieve more comprehensive blood sugar control and wound healing.

**Figure 12:**
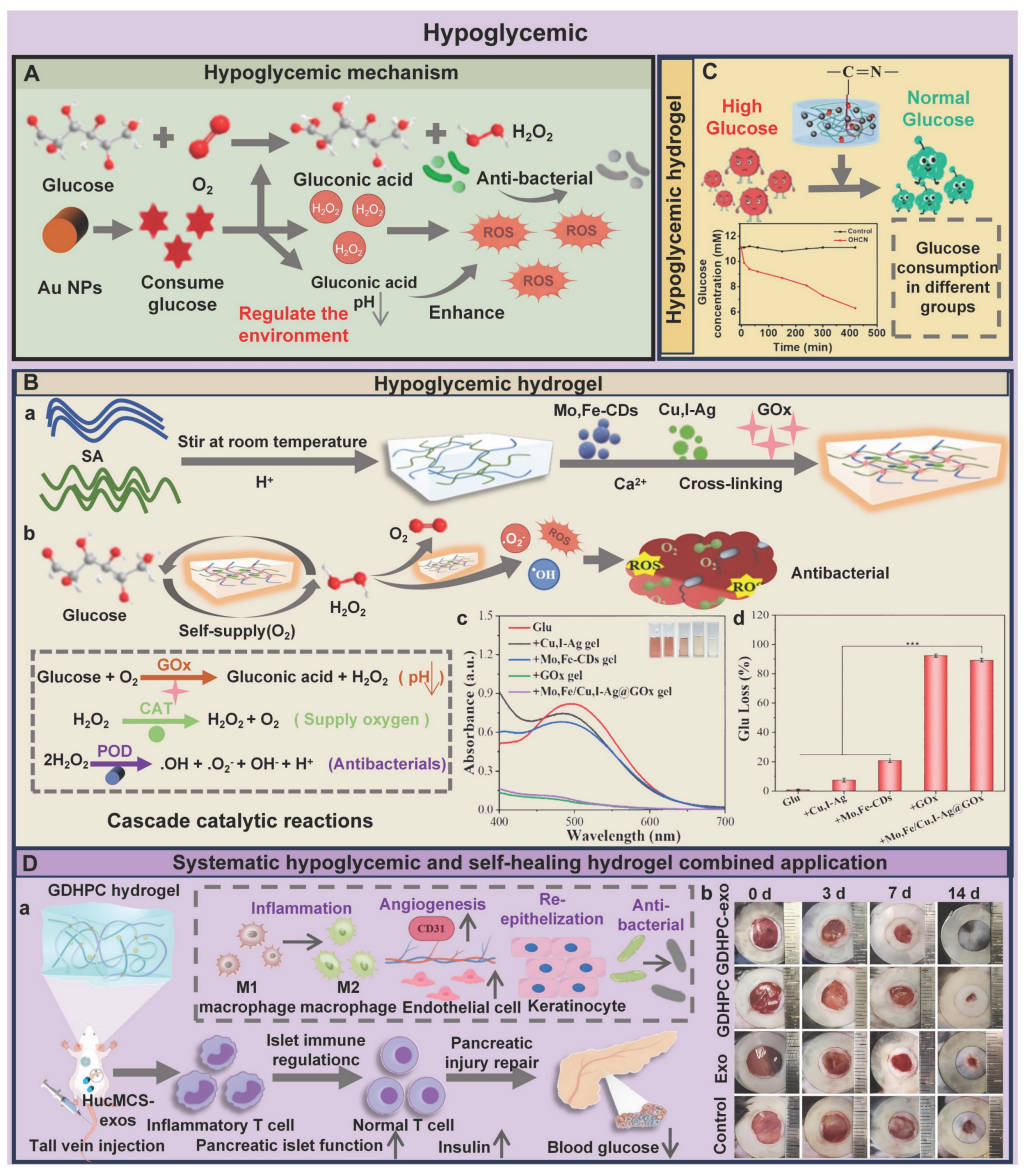
The hypoglycemic mechanisms and effects of PSHs. (A) Hypoglycemic mechanism of Gox-like nanoenzymes. Adapted with permission from 303, copyright 2025 Elsevier. (B): (a) Preparation process of Mo, Fe/Cu, I-Ag nanoenzyme hydrogels. (b) The nanoenzyme hydrogel catalyzes the degradation of glucose through a cascade reaction. (c) and (d) represent the glucose loss rates of different groups. Adapted with permission from 305, copyright 2024 Elsevier. (C) Schematic diagram of glucose consumption of OHCN hydrogel. Adapted with permission from 307, copyright 2022 Elsevier. (D): (a) Schematic diagram of GDHPC hydrogel combined with hucMSC-exos for repairing diabetic wounds from the inside out. (b) Wound healing process of diabetic mice in different treatment groups. Adapted with permission from 309, copyright 2025 Elsevier.

Among the numerous hypoglycemic active substances, GOx and GOx-like nanozymes can specifically bind to glucose, and catalyze its conversion into gluconic acid and H_2_O_2_ (Figure 12A) 302,303. Therefore, GOx and GOx-like nanozymes are regarded as ideal candidates for regulating the hyperglycemic microenvironment of diabetic wounds. GOx can effectively consume glucose on the surface of wounds and reduce the adverse effects of a high glucose microenvironment on wound healing. Gluconic acid produced by the GOx reaction helps to maintain the acidic environment of wounds, which can promote cell migration and proliferation, enhance the activity of enzymes related to wound healing, thereby accelerating the healing process of diabetic wounds 8. Moreover, H_2_O_2_ produced by the reaction can be further converted into highly active ·OH and generate a powerful antibacterial ability, which can effectively inhibit the growth of bacteria on the wound surface and reduce the risk of infection 304. In another study, nanoenzyme gels were prepared through multi-enzyme-like active nanocomposites (Mo, Fe/Cu, I-Ag@GOx), SA, and CS 305. Compared with other groups (without GOx), the nanoenzyme hydrogel group showed a faster rate of glucose consumption, further suggesting that GOx has outstanding hypoglycemic performance (Figure 12B). Additionally, the hydrogels also exhibited excellent antibacterial and antioxidant activities through cascade catalytic reactions initiated by GOx. Nevertheless, the natural enzyme GOx has limitations, such as poor stability and sensitivity to external environments (pH and O_2_). Compared with GOx, GOx-like nanozymes have lower costs, higher stability, and more functional catalytic activities 306. Zhang *et al*. 307 developed multifunctional hydrogels (OHCN) loaded with Au-Pt alloy nanoparticles (GOx-like nanozyme). OHCN could significantly reduce glucose concentration (11 m/M-6.5 mM), further suggesting that OHCN has excellent hypoglycemic activity (Figure 12C). In addition, Au-Pt alloy nanoparticles could simulate catalase (CAT), thereby endowing OHCN with multiple functions, including antioxidation and oxygen release. GOx-like nanozyme possessed CAT-like activity, which could effectively decompose the additional H_2_O_2_ produced during the reaction process, thus reducing the oxidative stress response at the wound site. Therefore, the cascade catalytic reaction of nanoenzymes can exert multiple functions, such as hypoglycemic, antibacterial, and oxygenation in the treatment of diabetic wounds 308. All in all, GOx and GOx-like nanozymes offer novel therapeutic strategies for wound management in diabetes. In the future, pivotal issues, including how to maintain the activity of GOx-like nanozymes, enhance the biological safety of multifunctional hydrogels, and achieve their clinical transformation, still need to be urgently addressed. Diabetic patients are often in a state of systemic hyperglycemia for a long time. Relying solely on local glucose consumption is difficult to fundamentally solve the problem of sugar metabolism imbalance in the wound microenvironment, which limits the long-term effectiveness of hypoglycemic hydrogels in the treatment of diabetic wounds. To address the limitations, we can combine the local application of PSHs with systemic hypoglycemia, thereby achieving comprehensive blood sugar control and promoting wound healing. Tang *et al*. 309 adopted a synergistic therapeutic strategy of systemic administration of human umbilical cord mesenchymal stem cells (hucMSC-exos) and self-healing hydrogels. This strategy could effectively maintain glucose homeostasis and improve the microenvironment of wounds, thereby achieving comprehensive repair of diabetic wounds from the inside out. More importantly, hucMSC-exos possessed the functions of restoring pancreatic islet function and lowering blood glucose levels, thereby reducing the induction of persistent hyperglycemia at the wound site. Compared with the application of self-healing hydrogels or hucMSC-exos alone, this synergistic treatment strategy achieved better and faster full-thickness wound healing in type Ⅰ diabetic mice, which provided a targeted solution for regulating blood sugar and promoting the comprehensive repair of diabetic wounds (Figure 12D). In the future, we should adopt combined treatment strategies of systemic hypoglycemia and local application of self-repairing hydrogels, which can better achieve the management and repair of diabetic wounds.

In summary, the high-glucose microenvironment of the wound can be effectively regulated by adopting local hydrogel dressings and local-systemic synergistic treatment methods, thus promoting the healing of diabetic wounds. Presently, PSHs loaded with GOx-like nanozymes have become a research hotspot, which can achieve multi-functional synergistic treatment, including local hypoglycemia, antibacterial, and antioxidant effects through cascade catalytic reactions. Unfortunately, the metal components in nanozymes pose potential toxicity risks, which may have adverse effects on tissues and cells. In the future, we should focus on optimizing the safety and biocompatibility of nanozymes. Additionally, we should develop intelligent responsive hydrogel dressings to improve the microenvironment of wounds and combine them with systemic hypoglycemia, which provides a more effective solution for promoting the healing of diabetic wounds.

### 6.6. Relieve hypoxia

The microvascular lesions in diabetic wounds and the decreased oxygen-carrying capacity of hemoglobin are important causes of tissue hypoxia 310. Chronic hypoxia reduces ATP synthesis through inhibiting mitochondrial oxidative phosphorylation and induces cellular energy metabolism disorders, thus inhibiting fibroblast activation, reducing collagen synthesis, and hindering ECM remodeling 311,312. Additionally, energy metabolism disorders can weaken the immune response mediated by neutrophils, which exacerbates wound inflammation 313. Consequently, improving the hypoxic environment of wounds is an urgent issue that needs to be addressed at present. However, traditional treatment methods are difficult to maintain and deliver sufficient oxygen to the wound. For example, hyperbaric oxygen (HBO) therapy is unable to sustain elevated oxygen concentrations at the wound site for extended periods and incurs significant costs. Moreover, the efficacy of local gaseous oxygen (TGO) therapy is limited, which is attributed to the difficulty of oxygen fully penetrating deep tissues 314. In recent years, oxygen-releasing hydrogel dressings have been regarded as an effective method for achieving long-term oxygen supply and promoting wound healing. Currently, the therapeutic strategies of PSHs for improving hypoxic environments mainly include 253,315: 1) Loading oxygen-releasing materials (H_2_O_2_, calcium peroxide, perfluorocarbon) on PSHs, which continuously supply oxygen to the local area of the wound; 2) PSHs loaded with enzymes (natural enzymes or nanoenzymes), thus catalyzing the generation of oxygen at the wound site; 3) PSHs combine with autonomous photosynthetic organisms to achieve sustainable and controllable oxygen supply.

**Figure 13:**
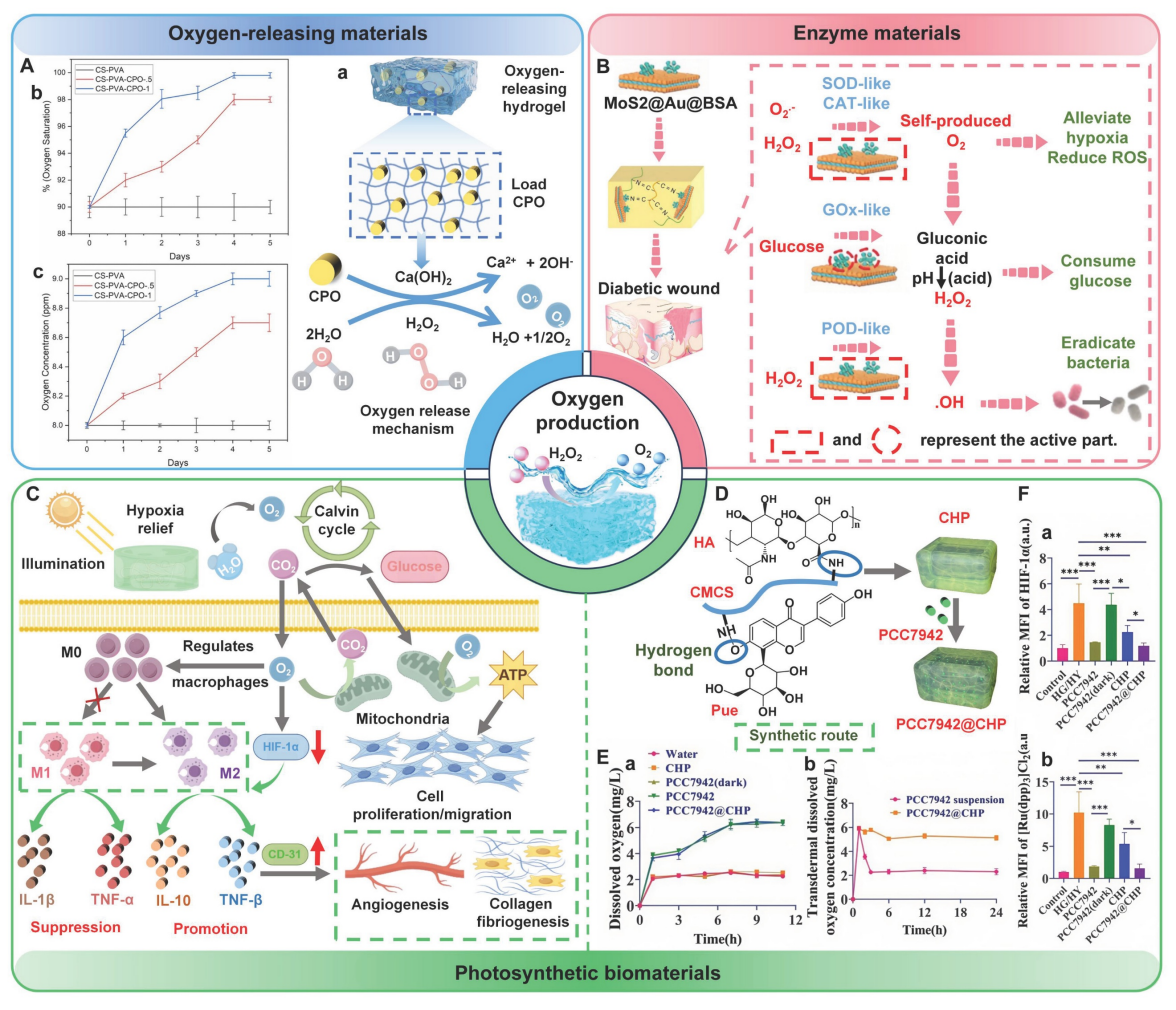
Mechanisms and effects of PSHs in alleviating hypoxia. (A): (a) Oxygen release mechanism of hydrogels loaded with CPO. (b) and (c) represent the changes in dissolved oxygen content and oxygen saturation caused by CS-PAVa-CPO hydrogel. Adapted with permission from 319, copyright 2023 Elsevier. (B): Cascade catalytic reaction initiated by oxygen self-supply of MoS2@Au@BSA hydrogel. Adapted with permission from 321, copyright 2022 Wiley. (C): (a) Schematic diagram of the oxygen release and wound healing promotion mechanism of microalgae hydrogel. Adapted with permission from 315, copyright 2024 Elsevier. (D): PCC7942@CHP hydrogel preparation process. (E): (a) Changes in the concentrations of CPH, PCC7942 (dark), PCC7942, and PCC7942@CHP dissolved oxygen within 11 h. (b) The content of dissolved oxygen penetrating the skin produced by PCC7942 suspension and PCC7942@CHP hydrogel at 37 °C. (F): (a) Relative fluorescence intensity of [Ru(dpp)3]Cl2 in HG/HY-HUVECs cells (human umbilical vein endothelial cells induced by hyperglycemia and hypoxia) treated with PCC7942, PCC7942 (dark), CHP, and PCC7942@CHP. (b) Relative fluorescence intensity of HIF-1α in HG/HY-HUVECs cells treated with PCC7942, PCC7942 (dark), CHP, and PCC7942@CHP. Adapted with permission from 325, copyright 2024 Elsevier.

Commonly used oxygen-releasing materials include calcium peroxide (CPO), H_2_O_2_, and perfluorocarbons 316. Among them, perfluorocarbons have problems, such as potential toxicity and difficulty in degradation. Moreover, the decomposition rate of H_2_O_2_ is excessively rapid, which poses challenges for the continuous and stable release of oxygen 317. CPO possesses excellent degradability and biocompatibility. In addition, the oxygen release rate of CPO is controllable, which can prolong the oxygen release time, thereby effectively reducing tissue hypoxia and promoting tissue repair, which is conducive to the wide application of CPO in the biomedical field 318. Relevant research has shown that loading CPO into the chitosan-polyvinyl alcohol (CS-PVA) hydrogel matrix could prepare hydrogels with a continuous oxygen release function (CS-PVA-CPO) 319. Compared with other groups, the CS-PAV-CPO group exhibited a higher percentage of dissolved oxygen and oxygen saturation (Figure 13A). Additionally, the hydrogels could provide at least five days of slow and continuous oxygen delivery. Furthermore, the CS-PVA-CPO group could contract the wound better than the control group, thereby accelerating the wound healing process. Accordingly, this study provides an effective solution for promoting wound healing in diabetes. Nonetheless, the H_2_O_2_ produced during the oxygen release process of CPO is not easily decomposed, thereby causing oxidative stress damage to cells. To address this limitation, we can remove H_2_O_2_ via adding catalase to the hydrogels 320. In addition to loading oxygen-releasing materials, inducing O_2_ production through enzymatic catalysis is also a crucial therapeutic strategy for alleviating hypoxia in wounds. The hydrogels loaded with gold nanoparticles modified with bovine serum albumin (MoS2@Au@BSA) had cascade catalytic activity 321. MoS2@Au@BSA possessed the SOD and CAT activity (Figure 13B). Superoxide anions could be converted into H_2_O_2_ and O_2_ by simulating SOD. Additionally, CAT activity could decompose the generated endogenous and exogenous H_2_O_2_ into O_2_, which not only alleviated the hypoxia problem at the wound site, but also reduced oxidative stress damage, thereby promoting the healing of diabetic wounds. Consequently, the preparation of cascade reaction hydrogels can simulate the activities of multiple enzymes, which provides an innovative solution for the treatment of diabetic wounds.

At present, photosynthetic biomaterials, as a green oxygen delivery method, have attracted much attention in the fields of biomedicine and tissue engineering. Algae (microalgae, cyanobacteria) or photosynthetic bacteria (*Clostridium rheinalis*, *Streptococcus prostrata*, *Streptococcus passivum*, etc.) have been successfully integrated into the hydrogel matrix, which could provide continuous oxygen supply to wounds or tissues through their photosynthesis 312,322. For instance, microalgae have been proposed to be used as natural oxygen generators. Microalgae have the capability to produce oxygen in a continuous and controllable manner, thus effectively enhancing the hypoxic conditions of wounds (Figure 13C) 315. Moreover, microalgae also possess potential functions including anti-inflammation, antioxidation, and promoting angiogenesis 323. Hydrogels, as the “ideal medium” for algae, possess unique porous microstructures and significant water contents. Hence, hydrogels can provide long-term support for the survival and biological activities of algae 324. Su *et al*. 325 integrated microalgae PCC7942, HA, and puerarin (Pue) into CMCS-based hydrogels to prepare multifunctional composite hydrogels (PCC7942@CHP) (Figure 13D). Microalgae PCC7942 endowed PCC7942@CHP hydrogels with photosynthetic oxygen release function. PCC7942@CHP hydrogels could not only continuously and stably release O_2_, but also effectively penetrate the generated O_2_ into the skin, thereby enhancing intracellular oxygenation and alleviating tissue hypoxia (Figure 13E). Besides, long-term oxygen supply could obviously reduce the expression of HIF-1α at the wound site and alleviate hypoxia to the greatest extent (Figure 13F). Zhu *et al*. 326 prepared oxygen-producing bilayer hydrogels using sodium alginate oxide (OSA) and CMCS as the inner layer through Schiff base reaction, and cyanobacteria, agarose, and CMCS as the outer layer. The bilayer hydrogels loaded with cyanobacteria exhibited stable oxygen production performance within 21 d. In addition, O_2_ produced by cyanobacteria could rapidly diffuse into cells and tissues through inner hydrogels, thereby alleviating hypoxia in wounds. Furthermore, continuous oxygen supply could effectively promote cell proliferation and migration, reduce inflammatory responses, and promote angiogenesis, further suggesting that continuous and adequate oxygen supply plays a decisive role in the repair of diabetic wounds. All in all, PSHs can integrate oxygen-releasing materials, enzyme catalytic systems, and photosynthetic microorganisms, which provide multiple oxygen supply strategies for diabetic wounds. Future research should focus on developing intelligent responsive oxygen release systems (such as light/enzyme/pH response) to achieve on-demand oxygen supply. In addition, we can combine genetically engineered photosynthetic microorganisms or develop new biomimetic nanoenzymes, which are expected to further enhance oxygen supply efficiency and therapeutic effects.

### 6.7. Synergistic therapy

Diabetic wound repair is a complex process, usually accompanied by bacterial infection, chronic inflammation, excessive oxidative stress, vascular damage, abnormal remodeling, etc. 327. Additionally, the healing of diabetic wounds requires precise treatment at different stages of hemostasis, inflammation, proliferation, and remodeling 328. The infiltration of numerous inflammatory cells can cause excessive inflammation and oxidative stress in the inflammatory stage, which requires timely anti-inflammatory and antioxidant treatment. In the proliferation stage, fibroblast migration and proliferation are impaired, and collagen synthesis diminishes, and angiogenesis is inadequate, and epidermal regeneration is delayed. This requires promoting cell migration and proliferation to form appropriate granulation tissue. The wound site needs to be provided with sufficient oxygen to meet the metabolic requirements of cells throughout the healing stage 329,330. Therefore, single-function hydrogels usually cannot meet the conditions required for wound healing. To address this issue, researchers, based on the mechanisms of difficult wound healing in diabetes, are dedicated to developing multifunctional hydrogels with synergistic effects. Multifunctional hydrogels possess various effects, such as alleviating excessive inflammation and oxidative stress, antibacterial, and promoting angiogenesis, which are of great significance for wound management in diabetes 331. Diabetic wounds are accompanied by damage to the skin and the immune barrier, which can promote bacterial proliferation. Hence, it is crucial to kill and inhibit bacteria in the management of diabetic wounds. Moreover, the inflammatory period after wound infection in diabetes is very fragile. Early anti-inflammatory treatment can effectively increase the wound closure rate 332. Accordingly, researchers often combine antibacterial, anti-inflammatory, and antioxidant activities to prepare multifunctional hydrogels, thus exerting a synergistic effect to promote wound healing 266,333. The hydrogels loaded with deferoxamine (DFO) and luteolin (LUT) could exert excellent synergistic therapeutic effects 334. The hydrogels not only possessed outstanding antibacterial, anti-inflammatory, and antioxidant properties, but also promoted cell proliferation and migration. The hydrogels released LUT and DFO at the wound site. DFO promoted angiogenesis and enhanced tissue regeneration by regulating HIF-1α and VEGF. Additionally, DFO could bind to excessive iron ions to eliminate ROS in chronic wounds. LUT possessed outstanding antioxidant, antibacterial, and anti-inflammatory functions. Therefore, the hydrogels could accelerate the wound healing process through multi-functional synergistic treatment. This comprehensive treatment breaks through the limitations of the single function of hydrogels and has greater prospects for clinical transformation. In recent years, nanomaterials have become the core components for constructing multifunctional hydrogels, which is attributed to their unique physicochemical properties and multiple biological activities (antibacterial, antioxidant, oxygen-releasing, pro-angiogenic, etc.) 335. For instance, AgNPs possess powerful antibacterial and anti-inflammatory properties. AgNPs can effectively combat various microorganisms (bacteria, fungi, and even antibiotic-resistant bacteria) and regulate inflammatory responses. In addition, gold nanoparticles (AuNPs) have anti-inflammatory and antioxidant properties. AuNPs can reduce oxidative stress and inhibit excessive inflammation, which creates a more favorable environment for wound healing 336. Notably, organic nanomaterials (EVs) can simulate natural biological processes and structures, thus attracting much attention in the field of diabetic wound healing 337. The microenvironment of diabetic wounds can be improved at all levels by encapsulating EVs in hydrogels. Consequently, multifunctional hydrogels with their synergistic therapeutic advantages can comprehensively regulate and improve the microenvironment of wounds, which is conducive to making them an ideal choice in the treatment of diabetic wounds. Notably, there are significant pathological differences between small animal models (mice and rats) and human wounds, which may pose potential challenges for the clinical application of multifunctional hydrogels. For instance, the skin structure of rats is loose, rich in hair follicles, and lacking sweat glands, and wound healing in rats mainly involves contraction. However, human skin is dense, and wound healing in humans relies on the filling of granulation tissue and re-epithelialization. Consequently, the wound healing mechanisms of rat and human skin are obviously different. Additionally, the methods used to induce diabetes in animal models (such as injecting streptozotocin) differ from the chronic metabolic disorders of human type Ⅱ diabetes, which can affect the efficacy evaluation of multifunctional hydrogels. Furthermore, the immune background of animal models is different from that of humans, which can cause deviations between the inflammatory response, degradation rate, and angiogenic activity of materials in the body and the actual clinical results 338. In the future, we should further verify the healing-promoting effect of the multifunctional hydrogels in large animals (pigs)/humanized models, and accurately assess the clinical application and transformation potential.

## 7. The application of intelligent responsive PSHs in diabetic wounds

Diabetic wound healing is a highly dynamic and multi-factorial complex biological process. Intelligent responsive PSHs can promptly sense the dynamic changes in the wound microenvironment and achieve on-demand drug release, thereby meeting various therapeutic needs (antibacterial, antioxidant, and anti-inflammatory). According to the source of the triggering signal, the action mechanisms of intelligent stimulus-responsive PSHs can be mainly divided into two categories as follows 339: 1) Responding to internal microenvironment signals of the wound: pH, glucose concentration, ROS, and enzymes; 2) Responding to external physical stimuli: temperature, light, electrical signals, and ultrasound. The* in vivo* stimulation responses mainly utilize the pathological properties of diabetic wounds. The pH of diabetic wounds changes according to the condition of the wound. The properties can be utilized to trigger specific response behaviors of pH-sensitive hydrogels, such as swelling, degradation, and network dissociation, thus enabling the on-demand and controlled release of loaded active substances. Relevant study has shown that the molecular network of pH-sensitive hydrogels contained pH-sensitive groups (carboxyl and amino groups)/dynamic covalent bonds 340. Glucose-responsive hydrogels usually incorporate phenylboronic acid groups/glucose oxidase to achieve glucose sensing functionality 341. These hydrogels recognize the high glucose environment through competitive binding/enzymatic reactions, which enable on-demand drug delivery and local blood glucose regulation. For instance, Ren *et al*. 342 developed novel pH/glucose dual-responsive hydrogel dressings. The low pH and high glucose levels in diabetic infected wounds could cause the breakage of borate bonds, release hydroxytyrosol (a polyphenolic compound), and eliminate intracellular ROS, thereby alleviating oxidative stress damage. In addition to pH and glucose, ROS-responsive PSHs have also attracted much attention. These hydrogels usually incorporate ROS-sensitive groups (thioethers, thioketones, and oxalates). The hydrogels can trigger the controlled release of encapsulated drugs through structural transformation in a high concentration of ROS environment, regulate the local ROS concentration, exert biological activity, and promote wound healing. For example, Sun *et al*. 343 prepared a ROS-responsive “exploding hydrogel” through disulfide bond cross-linking. The hydrogel could achieve sequential release of functional molecules through a size-dependent sequential release effect, thus enabling temporal control of the wound healing process (antibacterial, inflammatory, and regenerative). Notably, MMPs are usually overexpressed in the microenvironment of diabetic wounds, which severely hinders the wound from entering the stage of tissue remodeling. Therefore, MMPs are regarded as a new target for treating chronic diabetic wounds. Previous study has shown that MMP-responsive hydrogels could incorporate gelatin as an enzyme-sensitive cross-linking point. Gelatin degraded in the microenvironment enriched with MMPs to regulate drug release 344. Lin *et al*. 345 prepared MMP-9-responsive hydrogels based on oxidized hyaluronic acid and gelatin. The gelatin degraded and continuously released cinnamaldehyde under the action of MMP-9, which inhibited ferroptosis of traumatic endothelial cells and accelerated wound healing in diabetic patients. Consequently, the *in vivo* stimulation signals (pH, glucose, ROS, and MMPs) provide key pathological targets for PSHs to achieve autonomous response, precise regulation, and on-demand treatment. Equally important, external physical stimuli (temperature, light, electrical signals, and ultrasound) provide remote, controllable, and non-invasive control means for PSHs, which are conducive to achieving precise spatiotemporal intervention in the treatment process. For instance, ultrasound is non-invasive, highly tissue-penetrating, and space-time controllable. Low-frequency (20-100 kHz)/high-frequency (20.7 MHz) ultrasound waves, as the stimulation source, can induce controlled drug release. Previous study has designed ultrasound-responsive hydrogels (MCF@CA) that could target the delivery of nanoparticles 346. MCF@CA triggered the decomposition of calcium cross-linking networks through ultrasound to achieve controllable drug release rates, thereby enabling future adjustments according to different clinical needs. Compared to the control group, the MCF@CA + ultrasound group had a higher wound healing rate, further suggesting that the ultrasound-responsive hydrogels show broad application prospects in diabetic wounds. In recent years, light-responsive PSHs have provided a new approach for non-contact/remote control in biomedical operations, which was attributed to their advantages of non-invasiveness, low allergenicity, and high spatiotemporal resolution 347. Huang *et al*. 348 investigated the therapeutic effect of the near-infrared light-responsive multifunctional hydrogel in diabetic wounds. The hydrogel showed outstanding on-demand release behavior. The early release of AuPC (PC-functionalized gold nanorods) could exert broad-spectrum antibacterial activity. Subsequently, the release of AuPR (PR-functionalized gold nanorods) demonstrated excellent angiogenic promotion effects. More importantly, the local heat generated by near-infrared light stimulation could accelerate blood flow, eliminate bacteria, and stimulate fibroblast proliferation, which provided a promising strategy for the management of diabetic wounds. Therefore, the internal stimulation signals and external physical stimuli jointly enable PSHs to have the precise response capability to the microenvironment of diabetic wounds. Notably, there are several challenges in the application of intelligent stimulus-responsive PSHs as follows: 1) A single response mode is difficult to adapt to the dynamic evolution process of diabetic wounds; 2) The matching mechanism between response speed and drug release kinetics is not yet clear; 3) The biocompatibility and long-term* in vivo* stability of the response materials need to be systematically verified; 4) The reversibility and reusability of the response behavior have not been deeply studied. Consequently, future research should focus on the following aspects: 1) Developing intelligent PSHs with multi-stimulus cooperative responses to simulate the sequential evolution of wound healing; 2) Exploring the precise matching mechanism between response kinetics and therapeutic requirements; 3) Constructing reversible response systems to enable the multiple recycling of materials; 4) Verifying the biocompatibility and metabolic pathways of the response materials.

## 8. Clinical application of PSHs

PSHs have demonstrated remarkable versatility and therapeutic potential in the management of chronic wounds in diabetes. Nevertheless, the number of PSHs products that have actually entered clinical trials/clinical applications is still very limited 349. Several hydrogel products have entered clinical application in the field of diabetes wound management. For instance, REGRANEX® (Becaplermin gel) was approved by the FDA in 1997. REGRANEX® was a recombinant human platelet-derived growth factor (rhPDGF-BB) gel. This hydrogel was mainly used for the treatment of diabetic neuropathic foot ulcers via stimulating cell proliferation and angiogenesis. Nevertheless, a recent study has shown that long-term use of REGRANEX® may increase the risk of cancer 95. Another commercial product was PromogranTM hydrogel (composed of oxidized regenerated cellulose and collagen) 350. This hydrogel showed good antibacterial and hemostatic effects. Moreover, this hydrogel also had the advantages of easy application and time-saving. Currently, there are no public reports available regarding the adverse effects of PromogranTM hydrogel. Clegg *et al*. 351 summarized the latest progress in clinical trials of hydrogels. ALLO-ACS-DFU was a hydrogel composite product loaded with allogeneic mesenchymal stem cells. This product was in the phase II clinical trial stage. In addition, TTAX01, as a hydrogel based on decellularized human placental umbilical cord tissue, was mainly used for the treatment of difficult-to-heal diabetic foot ulcers. This hydrogel has entered the phase III clinical trial. Notably, the aforementioned hydrogel products lack good self-healing capabilities. Currently, self-healing hydrogel products that have entered clinical trials/clinical applications are still rarely reported. A breakthrough clinical trial with the identifier NCT04995354 (launched in June 2022) initially explored the potential application of self-healing hydrogels in cancer supportive treatment 352. This hydrogel was mainly used to promote mucosal tissue repair and regeneration. Although this study did not focus on the field of diabetic wounds, the experimental results provided important references for the clinical application of PSHs in the treatment of complex wounds. Hence, the clinical application of PSHs is still in the initial stage, and the majority of the research remains at the preclinical stage. A relevant study has shown that the clinical application of PSHs is closely related to biological safety, efficacy verification, and scalability. PSHs with the following advantages have greater potential for clinical translation 353: 1) Biocompatibility and multi-functionality: PSHs that have multiple therapeutic effects and biocompatibility are expected to promote wound healing in clinical research; 2) Small molecule structure: Small molecule PSHs can reduce production costs and are easy to scale up, which presents a broad application prospect in home healthcare environments; 3) Intelligent responsiveness: Intelligent responsive PSHs have high pathological specificity and abundant preclinical evidence, which is conducive to PSHs becoming ideal choices for preliminary clinical trials. In the future, we should continue to promote the standardization and normalization of the preclinical evaluation of PSHs, thereby accelerating the transformation of PSHs from the laboratory to practical clinical application.

## 9. Challenges and prospects

### 9.1. Large-scale production and manufacturing costs

The scalability of PSHs remains one of the key challenges in clinical translation. PSHs, as wound healing materials, have demonstrated significant efficacy in preclinical models. However, preparations that meet the standards for clinical-scale production are still relatively scarce. Laboratory preparations typically rely on small-scale manual operations, which are difficult to meet the requirements of industrial-scale production for repeatability, cost-effectiveness, and regulatory compliance 354. From a commercial perspective, the heterogeneity of raw materials and the complex multi-step manufacturing processes can significantly affect the development cost, production efficiency, and performance of PSHs, thereby limiting the large-scale production of PSHs. For instance, the batch-to-batch variations in natural polysaccharide raw materials (molecular weight and purity) can directly influence the cross-linking density, mechanical properties, and degradation behavior of hydrogels, which lead to inconsistent product quality 15. Additionally, natural polysaccharides often achieve self-healing properties and multi-functional integration through chemical modifications (oxidation, carboxymethylation, quaternization, etc.). These modification processes involve complex reaction steps, the utilization of organic solvents, and purification procedures, thus increasing the preparation cost and technical threshold 355. Furthermore, the synthesis cost of nanomaterials/bioactive factors is high, and their stability is limited, which further increases the overall production cost. Consequently, we are committed to developing low-cost and high-purity extraction processes for natural polysaccharides and exploring green and efficient strategies for synthesizing active substrates. Previous study has shown that alginate could be combined with green solvents (natural deep eutectic solvents) or integrated with complementary techniques (microwave-assisted extraction method, hot water extraction method, and ultrasound-assisted extraction method) 356. These strategies could increase the yield of algal polysaccharides by 2% to 6% and improve the biological activities and functional properties of polysaccharides. Similarly, engineering the microbial strains (recombinant *Bacillus subtilis* and *E. coli*) could achieve efficient production of HA (28.7 g/L), which effectively overcame the disadvantages of low yield, high cost, and immunogenicity in animal tissue extraction 357. Notably, the multi-component synergy systems of PSHs face the challenge of difficult, precise control of the interactions between components. Minor fluctuations in reaction conditions (temperature, pH, and ionic strength) may cause deviations in cross-linking kinetics, thereby affecting the network homogeneity and self-healing efficiency of hydrogels. Future research should focus on the following aspects: 1) Establish predictive models for inter-component interactions to achieve precise design and regulation; 2) Develop continuous flow microreaction technologies to ensure highly consistent reaction conditions; 3) Explore one-pot synthesis strategies and simplify multi-step modification processes; 4) Utilize synthetic biology methods to achieve low-cost large-scale production of active factors; 5) Promote modular formula design and reduce the complexity of components.

### 9.2. Disinfection and long-term storage

Sterility is a fundamental requirement for biological materials used for implantation and contact with living tissues (organs, bone defects, and wounds). Consequently, PSHs, as medical materials directly applied to wounds, must have reliable sterility assurance and long-term storage stability 358. Notably, developing efficient and functionally reliable sterilization solutions is a significant challenge in advancing hydrogels for specific applications. Traditional sterilization techniques have many limitations. For example, wet heat sterilization (high temperature and high pressure) can cause the collapse of the three-dimensional network of PSHs, the rupture of cross-linking bonds, and the inactivation of active factors. Additionally, radiation sterilization (gamma rays and electron beams) may lead to the degradation of polysaccharide chains and the disruption of dynamic covalent bonds, which can weaken the self-healing performance and mechanical strength of the hydrogels 359. Therefore, improper sterilization techniques can damage the stability and functional characteristics of PSHs and affect the therapeutic effect, thereby limiting their application potential in the field of diabetic wound treatment. Fortunately, the emergence of new technologies (such as ozone and supercritical CO_2_) has provided ideas for the aseptic guarantee of PSHs. For instance, ozone can effectively inactivate microorganisms at low temperatures due to its strong oxidizing ability, which is applicable to heat-sensitive PSHs materials. Moreover, ozone has the advantages of strong penetration power and no toxic residue. Supercritical CO_2_ can achieve deep sterilization of porous hydrogels under mild conditions (40 °C). Previous study has confirmed that supercritical CO_2_ treatment had very little impact on the mechanical properties, swelling behavior, and biocompatibility of various hydrogels (SA, polyurethane, and polyacrylamide), which was superior to traditional wet heat sterilization and gamma radiation 360. Apart from sterilization technology, long-term storage stability is another important challenge for clinical translation of PSHs. The three-dimensional networks of PSHs are prone to dehydration contraction, network collapse, and microbial growth in the hydrated state. Additionally, the active factors (growth factors and drugs) loaded in PSHs may undergo hydrolysis/oxidation and become inactive, thus affecting the clinical efficacy of the products 361. Therefore, we need to develop effective long-term storage strategies. For instance, freeze-drying combined with sealed packaging can obviously extend the storage period of PSHs, as well as maintain their structural and functional stability. Wu *et al*. 362 prepared a hemostatic and antibacterial chitosan/N-hydroxyethyl acrylamide (NHEMAA)/Ti3C2Tx composite freeze-dried gel (CSNT). The CSNT gel could maintain good appearance and functional properties for a long time (> 2 years), which indicated that the CSNT gel had excellent storage stability, further suggesting that freeze-drying combined with sealed packaging is feasible in extending the storage period of PSHs. In the future, we should further explore the protective mechanisms of new drying technologies (spray freeze-drying and supercritical drying) on the network structure and functional activity of PSHs. Additionally, we should actively develop intelligent responsive packaging materials (O_2_ scavengers and humidity indicators) to monitor the storage status of PSHs in real time.

### 9.3. Biological safety

Biological safety is the core prerequisite for the clinical transformation of PSHs. Notably, the current systematic assessment of the biological safety of PSHs still has significant deficiencies. Natural polysaccharides have excellent biocompatibility. Nevertheless, PSHs usually require the addition of exogenous components (inorganic materials, photothermal agents, and ultrasound sensitizers) to enhance the therapeutic effect on wounds, which increases the complexity of the biological safety assessment. Current studies mostly focus on the short-term assessment of the cytotoxicity and acute inflammatory response of PSHs. The potential long-term and biological effects of PSHs in the body remain unclear 363. For instance, most studies merely evaluate the cytotoxicity of materials through simple CCK-8 or Live/Dead staining, while lacking the potential impact of material degradation products/leakage of functional factors on cell behavior (migration, differentiation, and senescence). Moreover, the risks of hemolysis, coagulation, and complement activation after direct contact of PSHs with blood have not been fully verified, which is particularly crucial in the context of diabetic vascular disease. The absorption, distribution, metabolism, excretion, and accumulation behaviors of the functional components (active drugs, nanoparticles, and synthetic polymers) in the body are still unclear. Chronic wounds in diabetes patients require long-term treatment. Discrepancy between the rate of material degradation and metabolic clearance may lead to an increase in the concentration of inorganic ions/drugs in vital organs (liver and kidney), thus triggering potential long-term toxicity and abnormal immune responses 364. More importantly, the current safety evaluation systems have not adequately considered the effects of the high sugar, weakly alkaline, and high oxidative stress microenvironment of diabetic wounds on the degradation of materials, the release of components, and the oxidative stability. Consequently, future research should focus on the following aspects: 1) Establish full-cycle safety evaluation systems covering the entire behavior of materials within the body (absorption, distribution, metabolism, excretion, and accumulation); 2) Construct *in vitro* assessment models that simulate the pathological microenvironment of diabetes, revealing the degradation behavior of materials and the kinetics of component release; 3) Explore the organ accumulation risks of functional components and the interaction between the body's immune network; 4) Develop traceable and trackable imaging techniques to achieve real-time monitoring and safety assessment of PSHs in the body.

### 9.4. Clinical operational convenience

The convenience of clinical operations is a crucial guarantee for the clinical application of PSHs in treating diabetic wounds. Excellent operational convenience can shorten the dressing change time, alleviate the patient's pain, improve the use compliance of medical staff, thereby enhancing the overall treatment outcome 365. In recent years, numerous studies have been dedicated to developing various forms of PSHs to accommodate different wound types and clinical scenarios. For example, injectable hydrogels have gained attention due to their minimally invasive nature, ability to fill irregular wounds, and capacity to carry therapeutic factors. The injectable hydrogels can achieve in-situ cross-linking without external triggering, which effectively simplifies the operation process and reduces the biological safety risks. Moreover, the outstanding pre-injection fluidity ensures the close adhesion of PSHs to irregular wounds and tissue attachment 366. Spray-type hydrogels can quickly cover large areas of wounds and achieve uniform film formation, which is suitable for wounds with significant exudation or irregular shapes. Additionally, spray-type hydrogels have the advantages of high cell loading efficiency, convenient application, rapid and immediate effect, and good spatial controllability 367. Chao *et al*. 368 developed novel sprayable hydrogels based on carboxymethyl chitosan (CMCS) and oxidized hyaluronic acid (OHA). The hydrogels could rapidly form and cover the wound site after spraying. Moreover, the hydrogels continuously release AuNPs-C₃N₄ nanoenzymes through gradual degradation, thereby exerting excellent antibacterial and antioxidant activities. Stimulus-responsive hydrogels can gelate in situ under the stimulation of body temperature/wound microenvironment, which further enhances the convenience and adaptability of operation 369. Nevertheless, the above-mentioned forms still encounter several limitations in clinical application as follows: 1) The cross-linking speed of injectable hydrogels needs to be precisely controlled. Too fast injection speed can easily to cause needle blockage, while too slow injection speed is difficult to effectively cover the wound; 2) The shear force during the spraying/injecting process may lead to the inactivation/degradation of bioactive factors; 3) The differences in usage among different operators can affect the gelation effect and treatment consistency of the hydrogels. In the future, we should develop intelligent injection systems with controllable cross-linking speed and delivery carriers that protect active factors from shear damage. Additionally, we should establish standardized clinical operation guidelines and training programs, which are crucial for ensuring the full performance of the materials and improving the treatment effect.

### 9.5. Regulatory approval

Regulatory approval is a fundamental factor for the successful transformation of PSHs into clinical products. Currently, numerous studies have focused on the synthesis design and performance optimization of PSHs. Nevertheless, the number of PSHs products that have actually obtained regulatory approval and entered clinical application is still very limited. Cautious and strict regulatory processes can effectively ensure the safety of patients. Unfortunately, the high costs and lengthy approval cycle (7-12 years) have become the main obstacles restricting PSHs from laboratory research to clinical translation 370. The key regulatory approval elements mainly include 371: 1) Material safety and biocompatibility: PSHs must have low irritation and toxicity, and long-term stability; 2) Good manufacturing practice (GMP) compliance requirements: PSHs should maintain sterility and consistency simultaneously; 3) Regulatory classification: PSHs should have a clear management classification (medical devices, combination products, or advanced therapeutic drugs) and approval requirements under different regulatory systems. As a composite system composed of a polysaccharide matrix, a dynamic cross-linking network, and functional components, the regulatory classification of PSHs is still unclear, which directly affects the selection of the approval path and development cost. The FDA classified pure hydrogel dressings without active ingredients as a Class I medical device in 1999. However, the introduction of a drug/biological agent can change the regulatory classification of hydrogels. In particular, the biological activities of the type of hydrogels are positively correlated with the degree of strictness in approval 313. The EU (EMA) MDR regulations impose stricter dual compliance standards on PSHs containing pharmacologically active components (simultaneously complying with the MDR and the Medicines Directive 2001/83/EC), which increase the complexity and difficulty of the approval process 372. Material safety and biocompatibility are the core pillars of regulatory approval. Regulatory agencies, such as the FDA and EMA, attach great importance to conducting comprehensive and long-term studies on the biocompatibility, degradation characteristics, and product stability of PSHs. Additionally, batch-to-batch variability can delay the clinical translation process of PSHs and increase development costs. The differences in natural polysaccharide raw materials are not conducive to the precise reproduction of cross-linking density and mechanical properties 373. Currently, many preclinical studies lack large animal validation/scalable GMP protocols, which hinder clinical preparations. Consequently, a relevant study call for the establishment of a comprehensive translational research process as follows 374: 1) Accurately replicate humanized animal models of the diabetic microenvironment; 2) Scalable and reproducible synthesis protocols; 3) Adhere to ISO/USP standards for biological materials; 4) Generate robust pharmacokinetic-pharmacodynamic (PK-PD) data to support the investigational new drug (IND) application. In the future, we need to promote early collaboration among regulatory agencies, enterprises, and the academic community. Additionally, we also need to establish standardized evaluation systems for PSHs, which can accelerate the transformation of PSHs from the laboratory to clinical products.

## 10. Conclusion

PSHs show some advantages, including abundant sources, high safety, few side effects, and significant biological activities (antibacterial, anti-inflammatory, antioxidant, biocompatibility, etc.), which are expected to become potential candidate wound dressings for the treatment of diabetic wounds. Additionally, PSHs, as carriers, can also integrate a variety of excellent properties, which lay a solid foundation for their application in the field of diabetic wounds.

Firstly, this article summarizes the basic processes and characteristics of normal wound healing and diabetic wound healing. Compared with normal wounds, diabetic wounds have more complex pathological features (high glucose levels, hypoxia, infection, abnormal pH value, ROS accumulation, persistent inflammation, and abnormal MMPs levels). Accordingly, we urgently need to take effective measures to promote the healing of diabetic wounds. Then, this article introduces different types of PSHs. We have summarized that polysaccharides from different sources (CS, SA, cellulose, HA, chondroitin sulfate, etc.) possess various outstanding physicochemical properties and biological activities through extensive research, thereby effectively demonstrating their applicability in the field of diabetic wounds. In addition, this article also introduces the design strategies of PSHs, including physical non-covalent bonds and chemical covalent bonds. Numerous studies have demonstrated that hydrogels exhibited excellent self-healing properties through physical non-covalent bonds (hydrogen bonds, hydrophobic interactions, host-guest interactions, electrostatic interactions, and metal coordination) or chemical covalent bonds (imine bonds, acylhydrazone bonds, disulfide bonds, borate ester bonds, and DA reactions). Consequently, hydrogels can restore their structural and functional integrity after mechanical damage, which helps them adapt to dynamic changes in the wound surface and extend their service life. Notably, current studies mostly adopt physical/chemical multiple cross-linking strategies. The hydrogels prepared by these strategies can not only achieve self-repair, but also meet the complex wound requirements of diabetes in terms of mechanical strength, biological functionality, and environmental adaptability. Additionally, we focused on summarizing the therapeutic strategies and research progress of PSHs for diabetic wounds. PSHs possess numerous biological functions (antibacterial, antioxidant, anti-inflammatory, immunomodulatory, angiogenic, hypoglycemic, and oxygen-supplying), which can provide an effective solution for diabetic wound management. More importantly, PSHs prepared by combining two or more activities can exert synergistic effects, which significantly improve the complex pathological environment of diabetic wounds. Finally, this article discusses the challenges related to the clinical application of PSHs. In summary, PSHs have demonstrated significant value and promising prospects in the field of diabetes wound treatment. In the future, we should focus on building multi-functional intelligent responsive PSHs systems. Additionally, we need to continuously explore the dynamic interaction mechanism between PSHs and the microenvironment, promote the coordinated development of standardized evaluation and large-scale production, and accelerate the clinical transformation process of PSHs.

## Figures and Tables

**Table 1:** Comparison of normal wounds and diabetic wounds at different healing stages.

Wound healing stage	Normal wound	Diabetic wound	References
Hemostasis stage	Transient ●The blood vessels constrict to prevent further blood loss. ●Platelets adhere and aggregate to form thrombi. ●Endogenous and exogenous coagulation pathways are activated, thus forming stable blood clots.	Chronic ●Insufficient oxygen can inhibit platelet function and impede the clotting pathway. ●High blood sugar leads to microcirculation disorders and increases the risk of bleeding.	25, 28, 46, 55
Inflammatory stage	Transient ●Mast cells release enzymes, histamine, and 5-HT, thus promoting vasodilation. ●Neutrophils clear bacteria and cell debris. ●Macrophages undergo phenotypic transformation, secrete growth factors, and promote tissue repair.	Chronic ●Hyperglycemia leads to the accumulation of ROS and induces oxidative stress. ●Hyperglycemia promotes the transformation of macrophages into M1 type, thereby secreting a large amount of pro-inflammatory factors (IL-1β, IL-6, and TNF-α). ●Bacterial infections exacerbate and trigger severe inflammatory responses.	30, 31, 33, 50, 51, 65
Proliferation stage	Easy ●Angiogenesis can provide more oxygen and nutrients, thus promoting tissue repair. ●Fibroblasts activate and contract the wound. ●Granulation tissue forms and fills the wound. ●Keratinocytes proliferate and migrate to complete wound coverage.	Difficult ●Impaired function of endothelial cells and fibroblasts leads to tissue regeneration disorders. ●Impaired keratinocyte function inhibits the formation of the epidermal barrier. ●Chronic hypoxia causes angiogenesis disorders and tissue ischemia, which hinders the wound healing process.	36, 37, 40, 53, 59, 60
Reshaping stage	Easy ●Collagen fiber remodeling enhances the tensile strength of the regenerated skin. ●MMPs degrade the excess ECM components. ●Most cells die in a programmed manner.	Difficult ●Elevated MMPs levels lead to an imbalance in ECM degradation, which can impede the wound healing process. ●Collagen breakdown. ●The migration of epidermal cells is blocked, thereby delaying re-epithelialization and wound closure.	42, 44, 68, 84, 87

**Table 2:** Chemical structures, sources, and wound healing promoting mechanisms of different polysaccharides.

Polysaccharides	Chemical structure	Source	Wound healing mechanism	References
CS (D-glucosamine is linked to N-acetyl-D-glucosamine through a β-1, 4-glycosidic bond.)	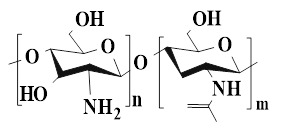	Chitin (Deacetylation)	●CS has excellent biocompatibility, antibacterial properties, and hemostasis. ● CS can promote the proliferation of vascular endothelial cells and fibroblasts, collagen deposition, and granulation tissue formation. ●CS can achieve rapid hemostasis through multiple pathways.	91, 93, 94
SA (β-(1,4)-D-manuronic acid and α-L-glucuronic acid)	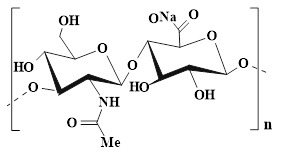	Brown algae and bacteria	●SA has high water absorption, which can effectively absorb wound exudate. ●SA can activate monocytes and promote the secretion of cytokines, thereby facilitating wound healing.	103, 106
Cellulose (D-glucose units are linked via β-(1,4)-glycosidic bonds)	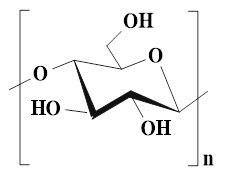	Wood pulp, cotton fluff, and microorganisms (bacteria)	●Cellulose has excellent biocompatibility, degradability, and high water absorption, thus exhibiting significant value in the field of wound healing. ●Cellulose-based hydrogels can retain a large amount of water, provide a moist environment, and promote cell migration and regeneration.	118, 119, 122, 123
HA (D-glucuronic acid, N-acetyl-D-glucosamine, β-(1,3) and β-(1,4) glycosidic bonds)	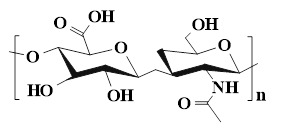	Animal connective tissue (Bird clams, cow's eyes, pigskin)	●HA, as a major component of ECM, has multiple functions, such as supporting angiogenesis, cell proliferation and migration, immune regulation, and tissue remodeling. ●HA can promote cell adhesion, stop bleeding, and alleviate inflammatory responses.	111, 134, 136
Chondroitin sulfate (Glucuronic acid and N-acetylgalactosamindisaccharide)	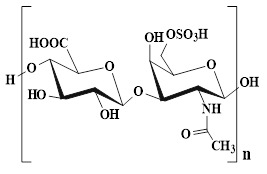	Animal cartilage	●Chondroitin sulfate has multiple biological functions (antibacterial, anti-inflammatory, and antioxidant). ●Chondroitin sulfate can promote the proliferation of fibroblasts and tissue regeneration. ●chondroitin sulfate regulates the wound healing cascade by interacting with multiple growth factors.	144, 145, 147
GG (β-D-pyranose and α-D-pyrangalactol)	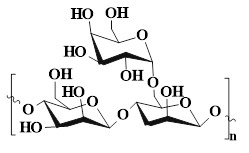	Guar bean	●GG has good biocompatibility and degradability. ●GG can induce phenotypic transformation of macrophages, alleviate inflammatory responses, and promote wound healing.	148, 149, 150
Dex (Glucose units are linked by α-1, 6-glycosidic bonds, and a few of their branches are connected by α-1,4, α-1,3, and α-1,2 bonds.)	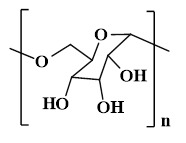	Lactic acid bacteria	●Dex can remove exudate from wounds, promote angiogenesis and granulation tissue formation, and stimulate collagen deposition and influence tissue remodeling.	129, 153, 154
ST	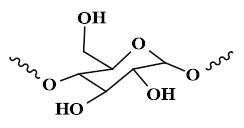	Potatoes, wheat, beans, corn, rice, and cassava flour	●ST has high water absorption and adhesion, which can achieve rapid hemostasis of wounds. ●ST can provide favorable conditions for cell proliferation and metabolism.	13, 156

**Table 3:** Examples of PSHs and their self-healing properties.

Name of hydrogel	Constituent materials	Self-healing mechanism	Healing condition	Healing efficiency	Mechanical properties	References
CG-Cu	CG and CuCl	Hydrogen bond	Room temperature, 1 h	-	The compressive modulus and compressive strength of CG-Cu1 hydrogel were 1.3 kPa and 89 kPa, respectively.	152
CMCS/PAM	CMCS and PAM	Hydrogen bond	Room temperature, 10 min	> 50%	The tensile strength of CMCS/PAM had increased by 300%, which was attributed to the introduction of a dual network structure.	166
BSP-U/DAHA	Ureido-pyrimidinone (UPy), bletilla striata polysaccharide (BSP), dopamine-conjugated di-aldehyde-hyaluronic acid (DAHA)	Hydrogen bond, imine bond, and metal coordination	Room temperature, 5 min	-	The compressive stress of BSP-U/DAHA-1 was (63.57 ± 3.38) %.	167
SA-RSF-C18M-30 HA	SA, C18M, RSF	Hydrophobic interaction	37 °C, 12 h	-	The compressive strength of SA-RFS-C18M-30 HA reached 17.1 kPa, and the Young's modulus reached 0.37 kPa.	175
DAMC/CHI-O	MC and CHI-O	Hydrophobic interaction and imine bond	37 °C, 20 min	-	The compressive stress of DAMC/CHI-O was 6.0 ± 0.42 kPa.	178
HPAAm/alginate-Ca^2+^ DN hydrogel	Alginate and HPAAm	Hydrophobic interaction and ion cross-linking	Room temperature	The recovery rates of elastic modulus and tensile stress were 3.47% and 93.34% within 30 min, respectively.	The fracture stress of the hydrogel was 1160.60 kPa, the fracture strain was 2604%, the elastic modulus was 71.79 kPa, and the toughness was 14.20 MJ m^-3^.	179
QCA	QCS, CD-SF, and AD-SF	Host-guest interaction	Room temperature	-	The strain value of the QCA20 hydrogel network reached 6823%.	188
AMDBA	β-CDsD, ALG-DOP, and silver nanoparticles	Host-guest interaction	Room temperature, 5 min	-	The G' value of the AMDBA-4 hydrogel reached 3380 and 12,100 Pa by applying angular frequencies from 126 to 500 Hz.	190
Gel-COS	GOx, HRP, COS, and phenolic alginate	Electrostatic interaction	37 °C	-	Gel-COS could restore its initial shape and thickness within 10 min under the pressure of a 200g heavy object.	202
CMCh-Zn	CMCh and Zn^2+^	Ionic bond	Within a few seconds	-	The G' value of CMCh-Zn4 was approximately 11,000 Pa.	204
PAAc/HACC	AAc and HACC	Ionic bond	70 °C, 48 h	63.1%	The fracture stress and Young modulus of PAAc/HACC were 3.31 MPa and 2.53 MPa, respectively.	205
TOCNF-Ca^2+^-T	TOCNF, Ca^2+^, and tannin	Metal coordination	Room temperature, 1 min	-	-	207
CS-PAA-Fe(III)	CS, Fe^3+^, and acrylic acid (AA)	Metal coordination and hydrogen bond	Room temperature, 12 h	93.8%	The tensile strength and elongation at break of the hydrogel were 20-264 kPa and 650%-2390% under different Fe^3+^ contents (0-0.5m), respectively.	209
CA-ECS/OP/Zn²⁺	CA-ECS, OP, and Zn^2+^	Metal coordination and imine bond	1 min	-	The mechanical properties of CA-ECS/OP/Zn²⁺ were 33.66 ± 0.59 kPa.	210
DCMC/CS-DA	CS-DA and DCMC	Imine bond	Room temperature, 24 min	-	The mechanical strength of DCMC/CS-DA could reach 1007.65 Pa.	222
OHPB	CS, flexible polymer PVA, and sodium tetraborate	Imine bond and borate ester bond	0.7 s	99.8%	The tensile rate and maximum compressive strength of OHPB were 700% and 25 kPa, respectively.	223
PLA/OSA/CMCS	CMCS, OSA, and, polylactic acid nanofibers (PLA)	Imine bond and hydrogen bond	10 min	-	The compressive strength and tensile strength of the hydrogel were 120 kpa and 19.22 kPa after incorporating 0.5% PLA nanofibers.	227
CMC-ADH/PEG-FBA	CMC-ADH and PEG-FBA	Acylhydrazone bond	Room temperature, 0.5 h	-	The G' value of the hydrogel increased from 580 Pa to 1580 Pa when the ratio of CMC-ADH/PEG-FBA increased from 4:1 to 1:1.	232
PBDS	SA-ADH, DCNF, PVA, and borax	Acylhydrazone bond and borate ester bond	Room temperature, 60 min	92.7%	The tensile strain and Young's modulus of PBDS were 1440% and 40.1 kPa, respectively.	233
CEC-l-OSA-l-ADH	CEC, ADH, and OSA	Imine bond and acylhydrazone bond	37 °C, 48 h	95%	The storage modulus (G') of the hydrogel increased from 717 ± 113 Pa to 5814 ± 166 Pa due to the increase of high-molecular-weight CEC (R varies within the range of 0 to 0.5).	237
NGel-ODex-ADH	NGel, ODex, and ADH	Imine bond and acylhydrazone bond	Room temperature, 1 h	-	The G' value increased from 600 to 1800 Pa as the NGel concentration increased from 50 to 100 mg/mL.	238
CPH	PHMB, Borax, HPMC, and COL/PVA	Borate ester bond and hydrogen bond	15 min	83.02%	The CPH-3 hydrogel exhibited highest tensile of 362.77% strain and maximum tensile stress of 25.6 kPa.	241
PGOP	PVA-GMA and OSA-PBA	Borate ester bond and hydrogen bond	30 min	(94.4 ± 2.5)%	The mechanical strength of PGOP was 39.5 ± 2.3 kPa.	242
CBT	PBA, CMCS, and TA	Borate ester bond and hydrogen bond	Room temperature, 1 h	The healing efficiencies of CBT-1, CBT-2, and CBT-3 hydrogels were (91.30 ± 3.56)%, (94.25 ± 2.68)%, and (80.64 ± 2.87)%, respectively.	The compressive strength of CBT-1, CBT-2, and CBT-3 gel was 0.31 Mpa, 0.21 MPa, and 0.067 MPa, respectively.	243

**Table 4:** Therapeutic strategies of PSHs in diabetic wounds.

Name of hydrogel	Biomaterials	Functional element	Hydrogel functions/therapeutic strategies	References
FA-Ag NPs/CPH	Pectin	FA-Ag NPs	●FA-Ag NPs/CPH had excellent antibacterial activity (the antibacterial rate against *MRSA* was 97.66% ± 1.10%). ●FA-Ag NPs/CPH could effectively eliminate ROS. ●FA-Ag NPs/CPH significantly down-regulated the expression of pro-inflammatory TNF-α and up-regulated the expression of anti-inflammatory IL-10.	261
DFO/CuS-ECM gel	HA	Cus and DFO	●The combination of DFO/CuS-ECM gel and photothermal exhibited outstanding antibacterial properties (with an antibacterial rate of over 99% against *E.coli* and *MRSA*). ●DFO/CuS-ECM gel could promote angiogenesis by inducing high expression of HIF-1α and VEGF.	262
CCG	CS	Cys and GA	●CCG could damage the cell membrane and cell wall of bacteria, thus affecting their normal metabolism. ●GA could provide hydrogen atoms and neutralize free radicals. ●CCG could form a protective barrier at the bleeding site and achieve rapid hemostasis.	271
QCT@HZ-CT	CMC	Quercetin and tannic acid	●QCT@HZ-CT could alleviate oxidative stress and protect mitochondrial and cellular functions. lQCT@HZ-CT had angiogenic-promoting, anti-inflammatory, and antibacterial activities.	273
TMP-HA	HA	TMP	●TMP-HA inhibited the function of M1 macrophages and improved the function of M2 macrophages. ●TMP-HA promoted wound angiogenesis by regulating macrophages, improved re-epithelialization, and enhanced collagen deposition.	284
CS-AT-Exo	CS-AT and PEG-DA	Exo	●CS-AT-Exo could promote angiogenesis and maturation, epithelial regeneration, and collagen deposition. ●CS-AT-Exo had anti-inflammatory and immunomodulatory functions.	294
BP/CS-bFGF	CMCS	bFGF	●BP/CS-bFGF could alter the permeability of bacterial cell membranes, inhibit DNA replication, and cause bacterial death. ●BP/CS-bFGF had excellent biological adhesion and hemostatic properties. ●BP/CS-bFGF promoted angiogenesis by up-regulating the expression of CD31 and CD34. ●BP/CS-bFGF promoted full-thickness wound repair through up-regulating Ki67.	100
CS-SNAP	CS	NO	●CS-SNAP exhibited excellent antibacterial and angiogenic promoting activities. ●CS-SNAP could promote fibroblast proliferation and migration, and enhance collagen deposition.	297
Mo,Fe/Cu,I-Ag@GOx gel	SA and CS	Mo, Fe/Cu, and I-Ag@GOx	●The hydrogel improved the high sugar environment of wounds by consuming glucose. ●The hydrogel could decompose endogenous and exogenous H_2_O_2_ into O_2_, thereby alleviating oxidative stress and hypoxia. ●The hydrogel possessed excellent antibacterial activity and angiogenic properties.	305
OHCN	OHA and CMCS	Au-Pt alloy NPs	●OHCN could improve intracellular oxidative stress and alleviate hypoxia. ●OHCN also exhibited excellent antibacterial, hypoglycemic, and angiogenic properties.	307
CS-PVA-CPO	CS-PVA	CPO	●CS-PVA-CPO had the characteristics of slow and continuous oxygen release. ●CS-PVA-CPO could increase the migration of endothelial cells and fibroblasts, which was beneficial for wound contraction.	319
ODex/gC/MoS2@Au@BSA	ODex and GC	MoS2@Au@BSA	●The hydrogel could destroy bacterial membranes and inhibit bacterial proliferation. ●The hydrogel could alleviate oxidative stress and provide oxygen. ●The hydrogel could promote re-epithelialization of wounds, collagen deposition, and angiogenesis.	321
PCC7942@CHP	HA and CMCS	Microalgae PCC7942 and Pue	●PCC7942@CHP could stably release oxygen and promote the penetration of dissolved oxygen into the skin. ●PCC7942@CHP could improve mitochondrial function and activate the antioxidant defense system, thereby effectively alleviating oxidative stress. ●PCC7942@CHP also had excellent angiogenic, anti-inflammatory, and antibacterial properties.	325
Gel1(Cyan)/Gel2(PCN)	OSA, CMCS, and agarose	Cyanobacteria	●The hydrogel demonstrated stable oxygen production performance within 21 d. ●The hydrogel could damage the cell membranes and walls of bacteria, thereby causing their death. ●Hydrogels could further reduce inflammatory responses, promote angiogenesis, cell migration, and fibrous tissue deposition by generating oxygen.	326
C/DFL	CMCS and 2-formylphenylboronic acid (2-FPBA)	DFO and LUT	● C/DFL had excellent bactericidal and anti-biofilm formation properties. ●The combined effect of DFO and LUT promoted wound healing and cell migration by up-regulating the expression of VEGF and HIF-1α. ●C/DFL exhibited excellent anti-inflammatory, antioxidant, and pro-angiogenic properties.	334

## Abbreviations

PSHs: polysaccharide-based self-healing hydrogels
CS: chitosan
HA: hyaluronic acid
vWF: von willebrand factor
ADP: adenosine diphosphate
TXA2: thromboxane A2
5-HT: 5-hydroxytryptamine
IGF-1: insulin-like growth factor 1
VEGF: vascular endothelial growth factor
PDGF: platelet-derived growth factor
EGF: epidermal growth factor
ECM: extracellular matrix
TGF-β: transforming growth factor β
MMPs: matrix metalloproteinases
TNF-α: tumor necrosis factor alpha
IL-1β: Interleukin-1β
IL-6: Interleukin-6
AGEs: advanced glycation end products
HIF-1α: hypoxia-inducible factor-1α
MMP-9: matrix metalloproteinase-9
TIMP: tissue inhibitors of metalloproteinases
SA: sodium alginate
ST: starch
Dex: dextran
bFGF: basic fibroblast growth factor
β-CD: β-cyclodextrin
AD: adamandane
OSA: oxidized sodium alginate
CMC: carboxymethyl cellulose
CNF: cellulose nanofibers
CNC: cellulose nanocrystals
BCN: bacterial nanocellulose
GG: guar gum
CMCS: carboxymethyl chitosan
DN: dual-network
PAM: polyacrylamide
DA: diels-alder
SOD: superoxide dismutase
CAT: catalase
TSG-6: TNF-stimulated gene 6
HIF-1α: hypoxia-inducible factor 1
MSCs: Mesenchymal stem cells
EVs: extracellular vesicles
Exos: exosomes
GOx: glucose oxidase
CPO: calcium peroxide
FDA: food and drug administration
EMA: European medicines agency
